# Supplementary material for: Essential Principles of Preoperative Assessment in Internal Medicine: A Case-Based Teaching Session
Source: MedEdPORTAL. 2021 Aug 5;17:11178. doi: 10.15766/mep_2374-8265.11178 (PMC8339074; doi:10.15766/mep_2374-8265.11178)
Supplement: Supplementary file 1 — Preop Assessment and Management Slideshow.pptxCases 1-3.docxCases 4-6.docxPre- and Postassessment.docx [file mep_2374-8265.11178-s001.zip › A. Preop Assessment and Management Slideshow.pptx]

## Slide 1
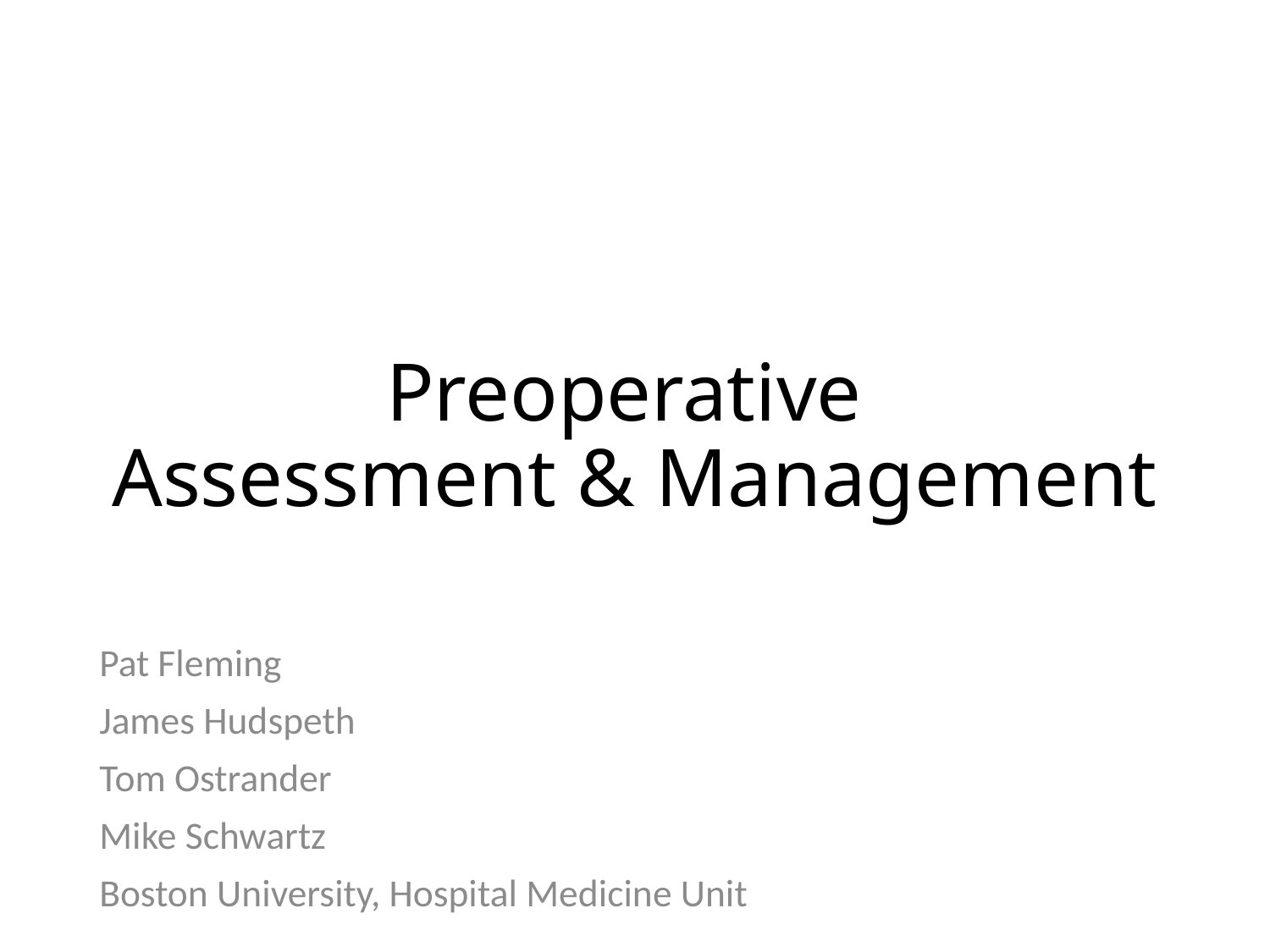

# Preoperative Assessment & Management
Pat Fleming
James Hudspeth
Tom Ostrander
Mike Schwartz
Boston University, Hospital Medicine Unit

## Slide 2
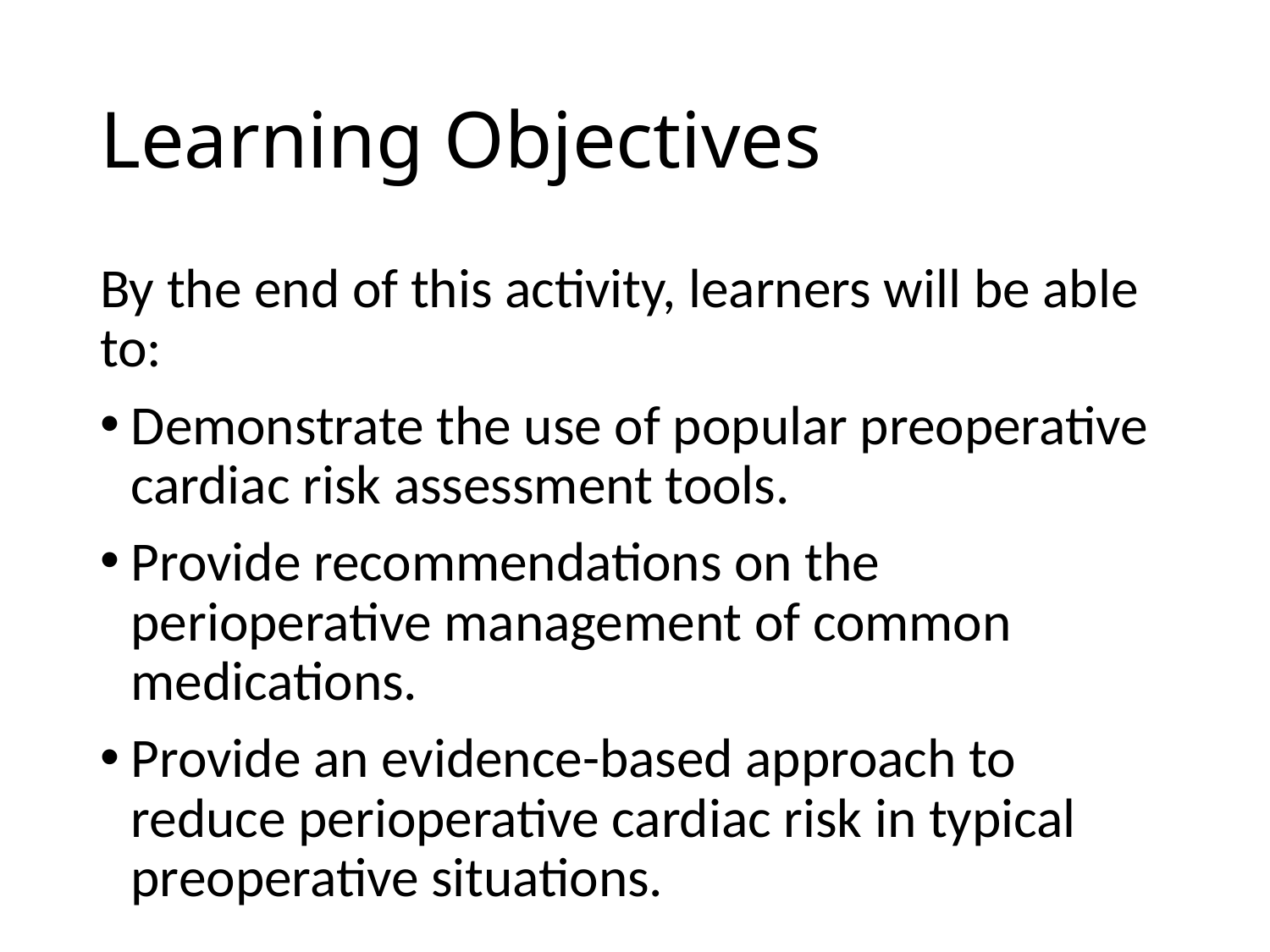

# Learning Objectives
By the end of this activity, learners will be able to:
Demonstrate the use of popular preoperative cardiac risk assessment tools.
Provide recommendations on the perioperative management of common medications.
Provide an evidence-based approach to reduce perioperative cardiac risk in typical preoperative situations.

## Slide 3
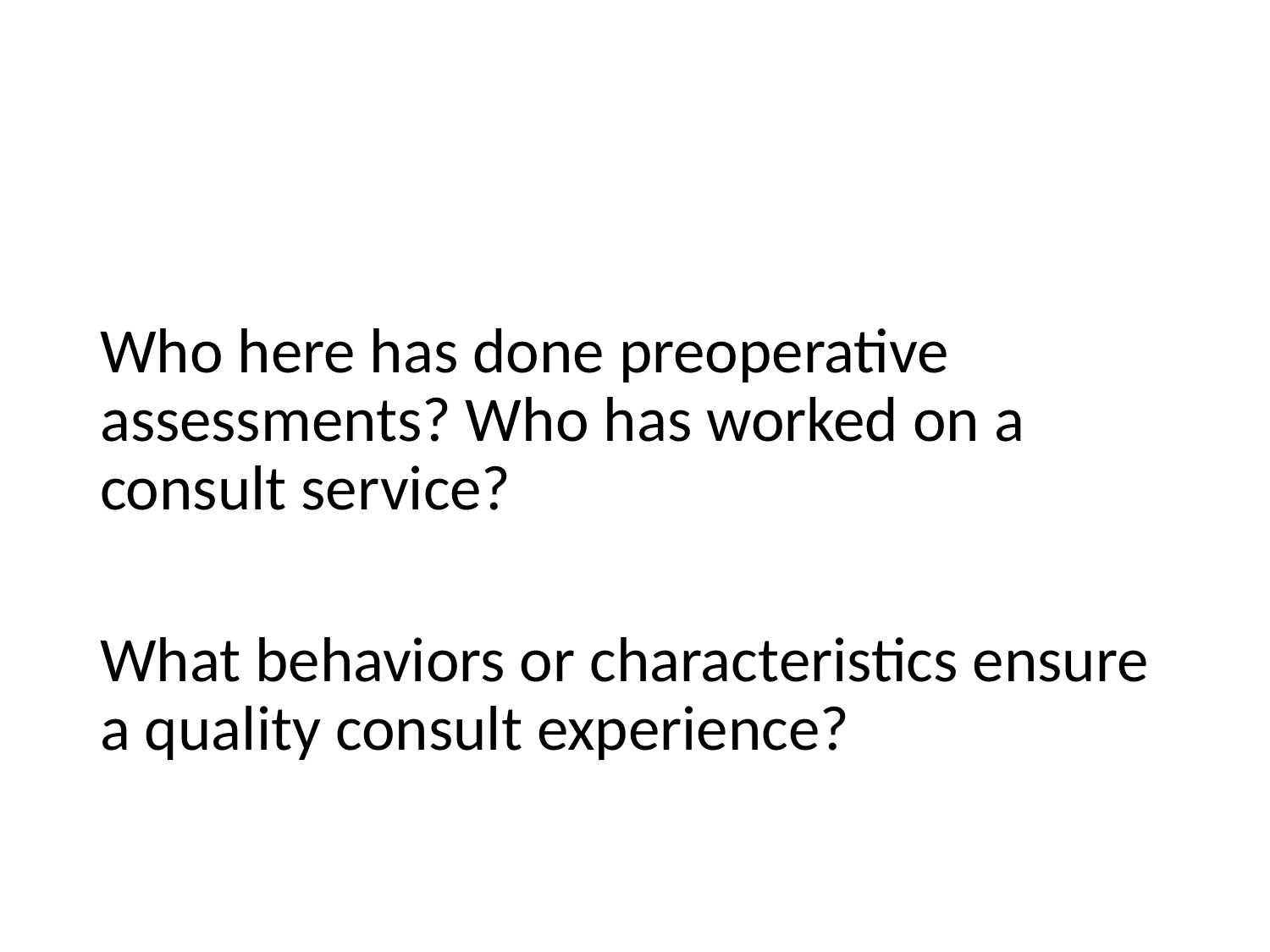

#
Who here has done preoperative assessments? Who has worked on a consult service?
What behaviors or characteristics ensure a quality consult experience?

## Slide 4
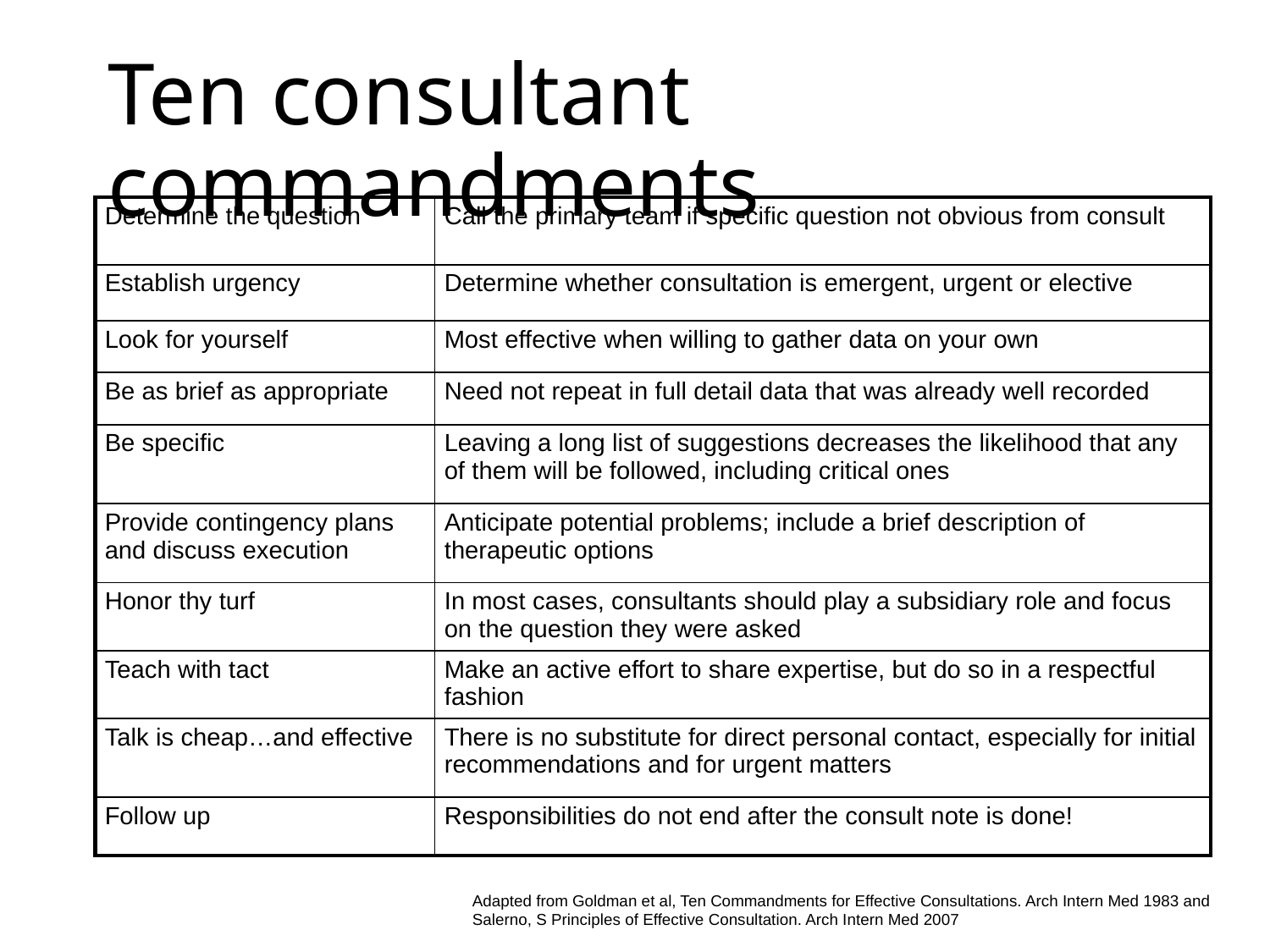

# Ten consultant commandments
| Determine the question | Call the primary team if specific question not obvious from consult |
| --- | --- |
| Establish urgency | Determine whether consultation is emergent, urgent or elective |
| Look for yourself | Most effective when willing to gather data on your own |
| Be as brief as appropriate | Need not repeat in full detail data that was already well recorded |
| Be specific | Leaving a long list of suggestions decreases the likelihood that any of them will be followed, including critical ones |
| Provide contingency plans and discuss execution | Anticipate potential problems; include a brief description of therapeutic options |
| Honor thy turf | In most cases, consultants should play a subsidiary role and focus on the question they were asked |
| Teach with tact | Make an active effort to share expertise, but do so in a respectful fashion |
| Talk is cheap…and effective | There is no substitute for direct personal contact, especially for initial recommendations and for urgent matters |
| Follow up | Responsibilities do not end after the consult note is done! |
Adapted from Goldman et al, Ten Commandments for Effective Consultations. Arch Intern Med 1983 and Salerno, S Principles of Effective Consultation. Arch Intern Med 2007

## Slide 5
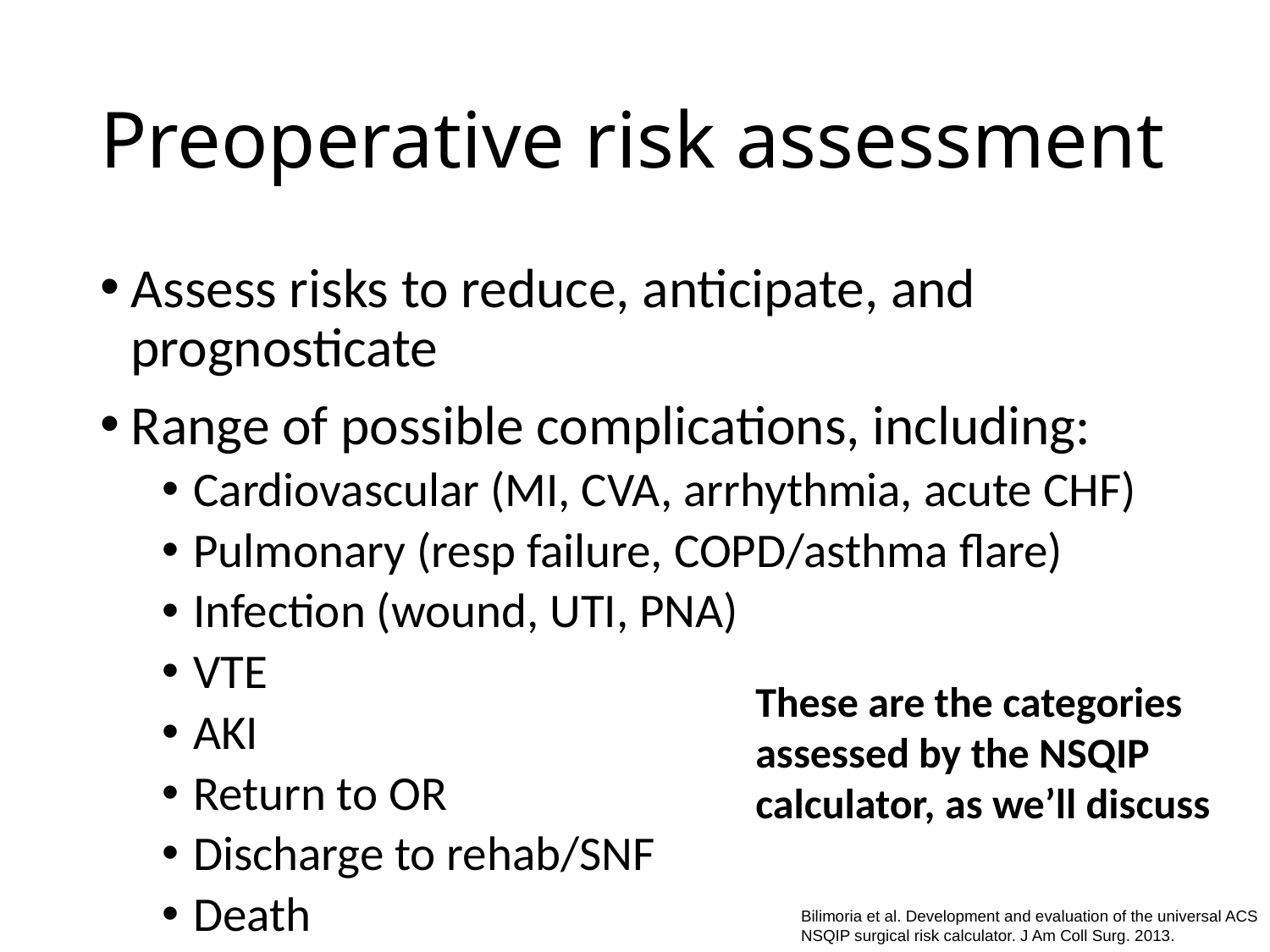

# Preoperative risk assessment
Assess risks to reduce, anticipate, and prognosticate
Range of possible complications, including:
Cardiovascular (MI, CVA, arrhythmia, acute CHF)
Pulmonary (resp failure, COPD/asthma flare)
Infection (wound, UTI, PNA)
VTE
AKI
Return to OR
Discharge to rehab/SNF
Death
These are the categories assessed by the NSQIP calculator, as we’ll discuss
Bilimoria et al. Development and evaluation of the universal ACS NSQIP surgical risk calculator. J Am Coll Surg. 2013.

## Slide 6
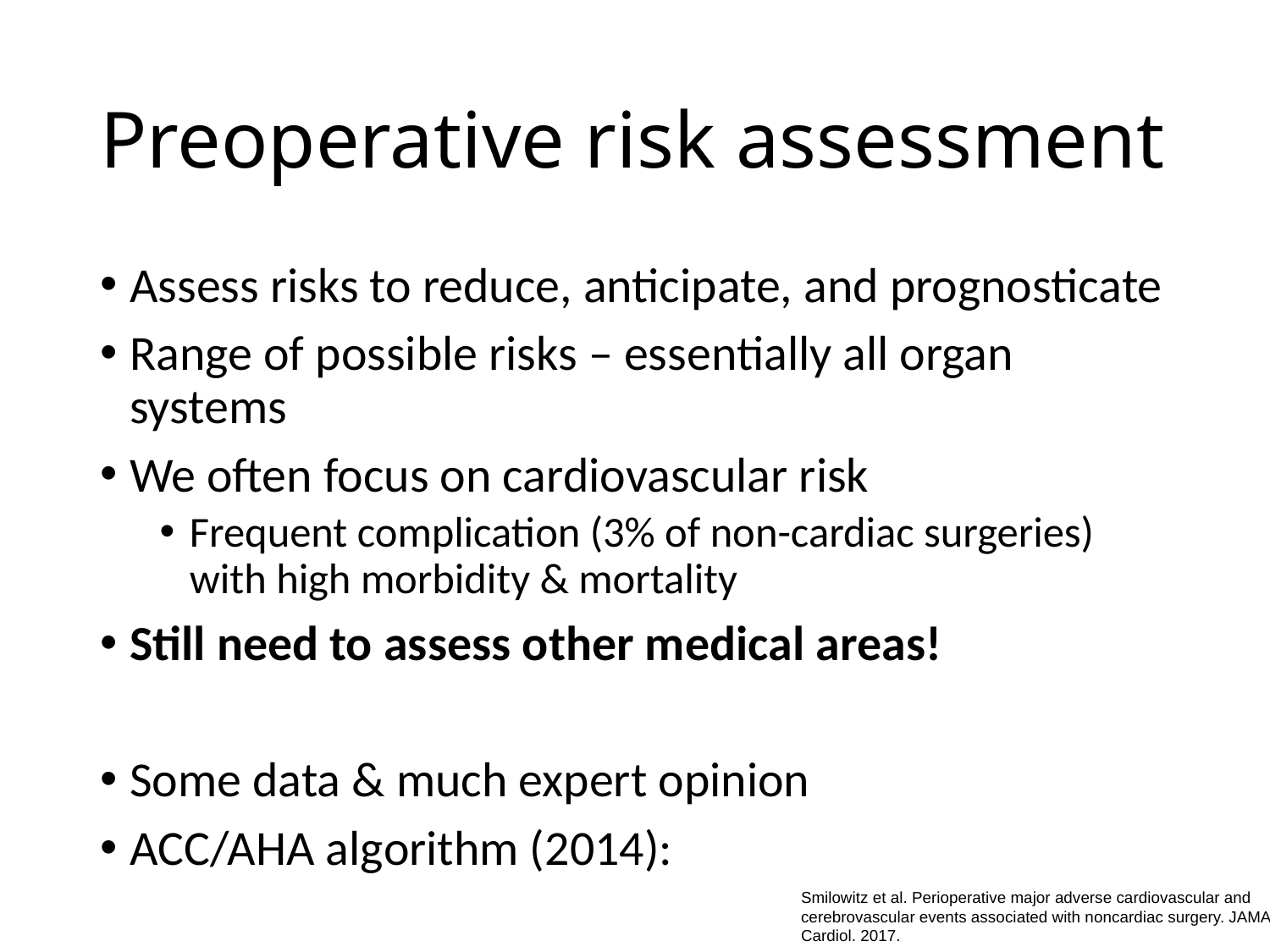

# Preoperative risk assessment
Assess risks to reduce, anticipate, and prognosticate
Range of possible risks – essentially all organ systems
We often focus on cardiovascular risk
Frequent complication (3% of non-cardiac surgeries) with high morbidity & mortality
Still need to assess other medical areas!
Some data & much expert opinion
ACC/AHA algorithm (2014):
Smilowitz et al. Perioperative major adverse cardiovascular and cerebrovascular events associated with noncardiac surgery. JAMA Cardiol. 2017.

## Slide 7
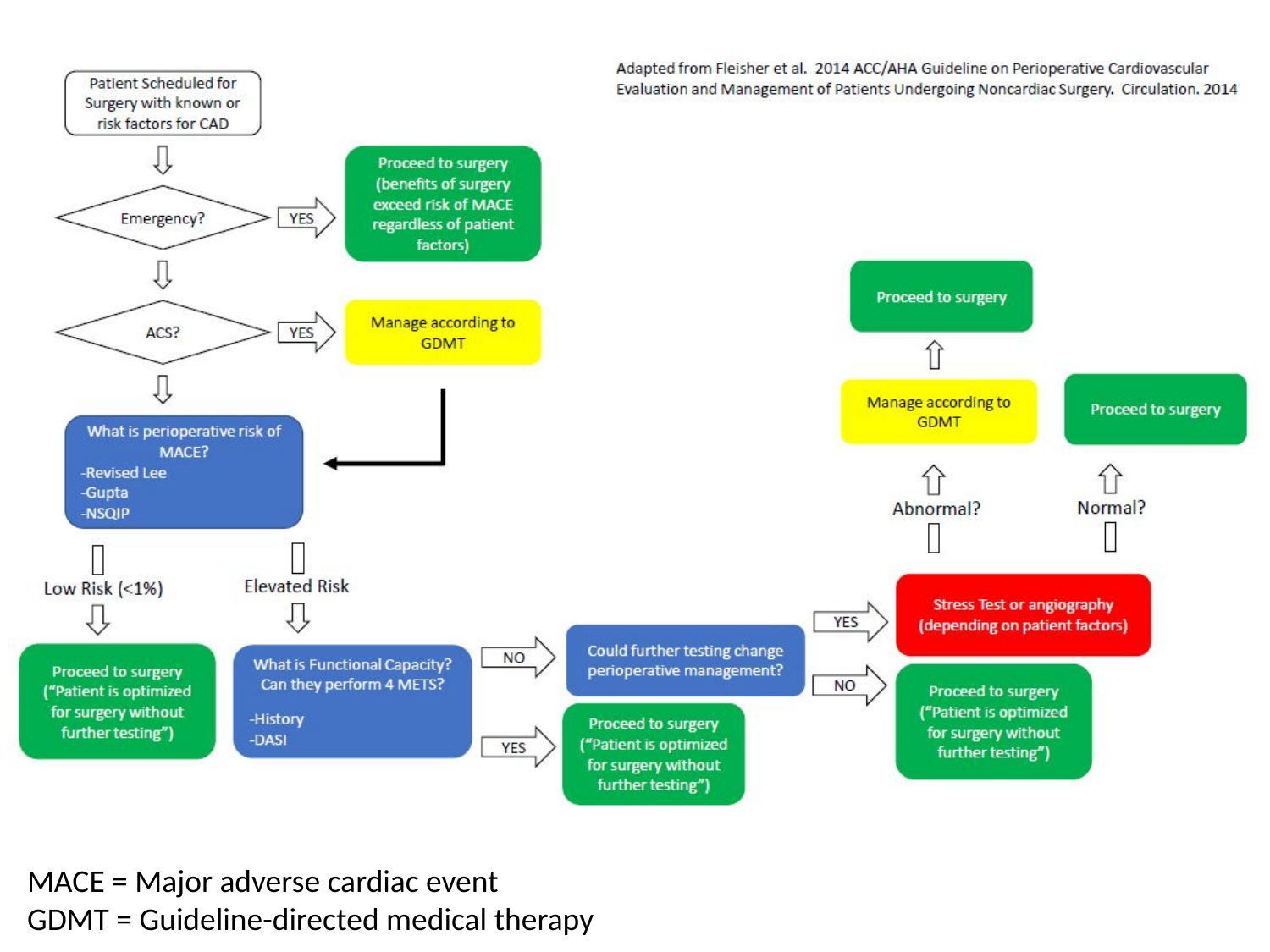

#
MACE = Major adverse cardiac event
GDMT = Guideline-directed medical therapy

## Slide 8
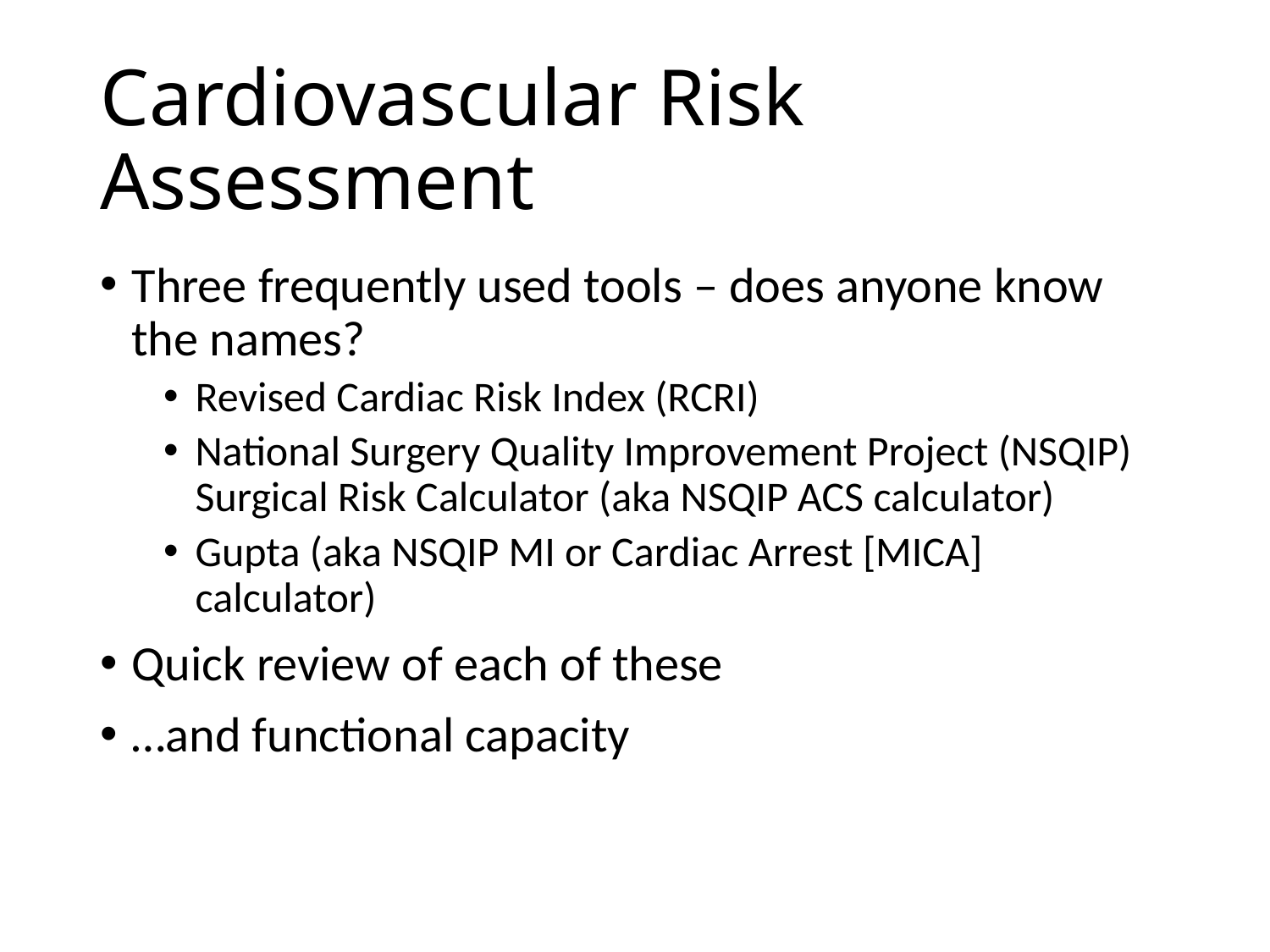

# Cardiovascular Risk Assessment
Three frequently used tools – does anyone know the names?
Revised Cardiac Risk Index (RCRI)
National Surgery Quality Improvement Project (NSQIP) Surgical Risk Calculator (aka NSQIP ACS calculator)
Gupta (aka NSQIP MI or Cardiac Arrest [MICA] calculator)
Quick review of each of these
…and functional capacity

## Slide 9
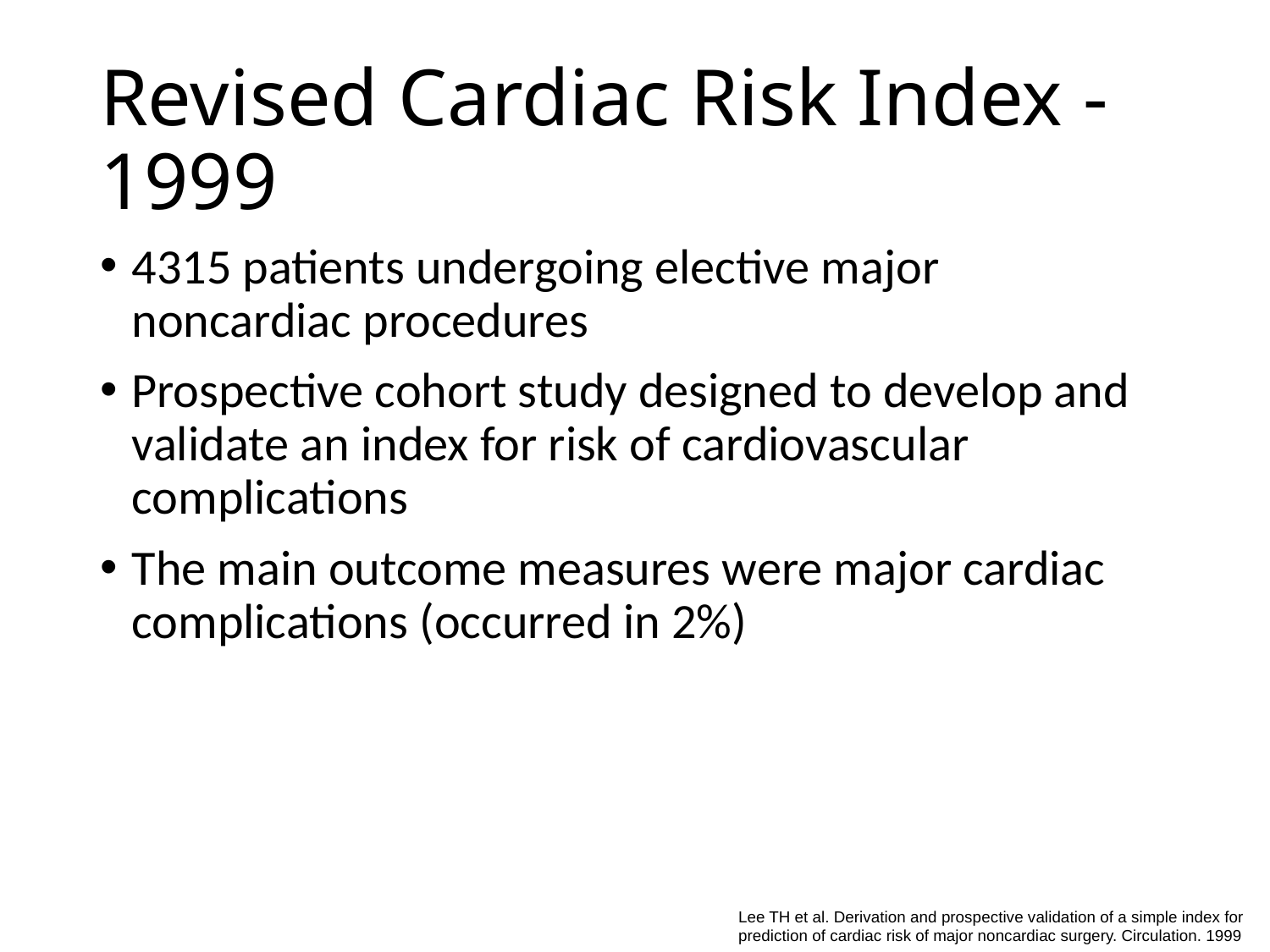

# Revised Cardiac Risk Index - 1999
4315 patients undergoing elective major noncardiac procedures
Prospective cohort study designed to develop and validate an index for risk of cardiovascular complications
The main outcome measures were major cardiac complications (occurred in 2%)
Lee TH et al. Derivation and prospective validation of a simple index for prediction of cardiac risk of major noncardiac surgery. Circulation. 1999

## Slide 10
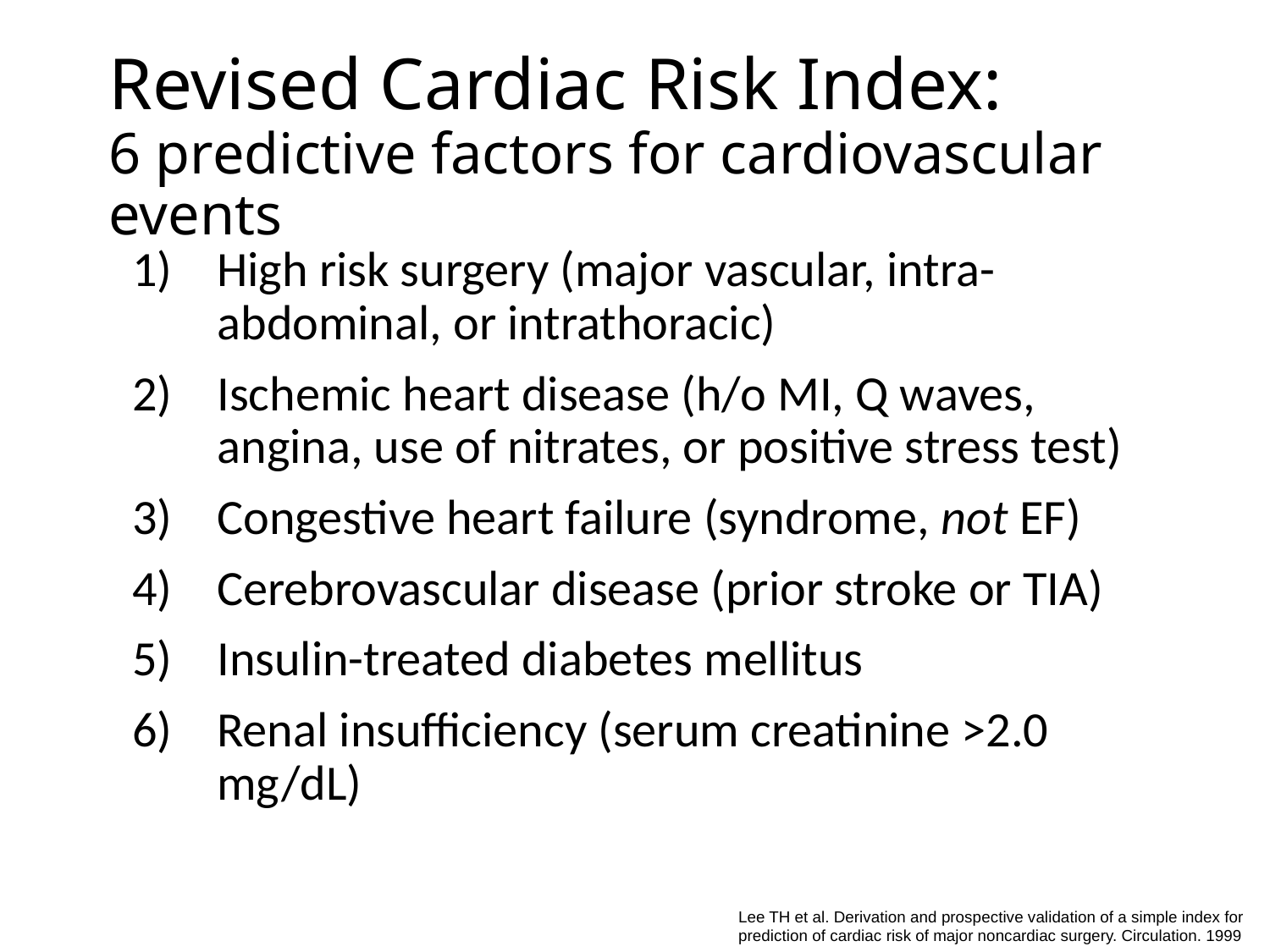

# Revised Cardiac Risk Index: 6 predictive factors for cardiovascular events
High risk surgery (major vascular, intra-abdominal, or intrathoracic)
Ischemic heart disease (h/o MI, Q waves, angina, use of nitrates, or positive stress test)
Congestive heart failure (syndrome, not EF)
Cerebrovascular disease (prior stroke or TIA)
Insulin-treated diabetes mellitus
Renal insufficiency (serum creatinine >2.0 mg/dL)
Lee TH et al. Derivation and prospective validation of a simple index for prediction of cardiac risk of major noncardiac surgery. Circulation. 1999

## Slide 11
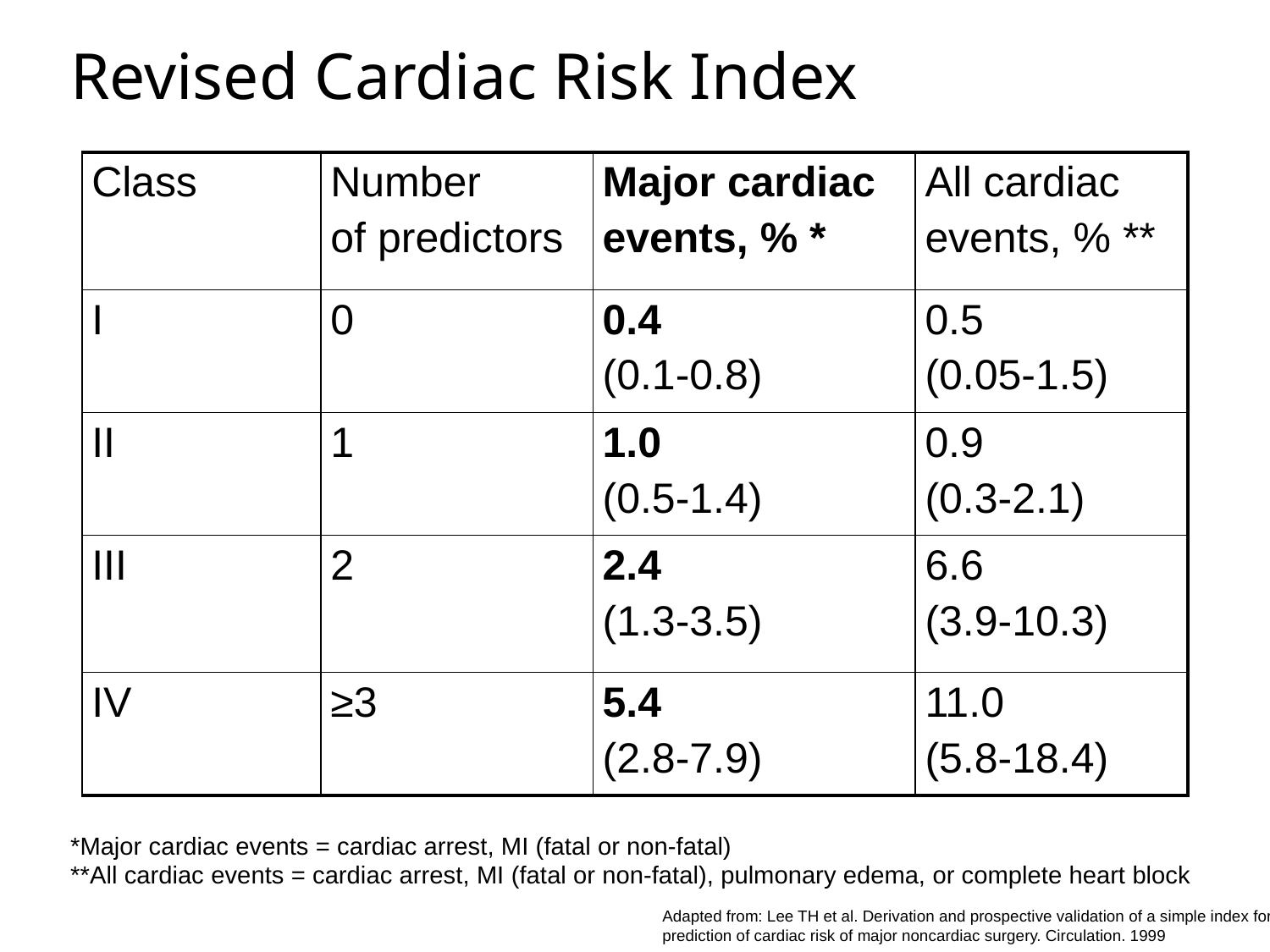

# Revised Cardiac Risk Index
| Class | Number of predictors | Major cardiac events, % \* | All cardiac events, % \*\* |
| --- | --- | --- | --- |
| I | 0 | 0.4 (0.1-0.8) | 0.5 (0.05-1.5) |
| II | 1 | 1.0 (0.5-1.4) | 0.9 (0.3-2.1) |
| III | 2 | 2.4 (1.3-3.5) | 6.6 (3.9-10.3) |
| IV | ≥3 | 5.4 (2.8-7.9) | 11.0 (5.8-18.4) |
*Major cardiac events = cardiac arrest, MI (fatal or non-fatal)
**All cardiac events = cardiac arrest, MI (fatal or non-fatal), pulmonary edema, or complete heart block
Adapted from: Lee TH et al. Derivation and prospective validation of a simple index for prediction of cardiac risk of major noncardiac surgery. Circulation. 1999

## Slide 12
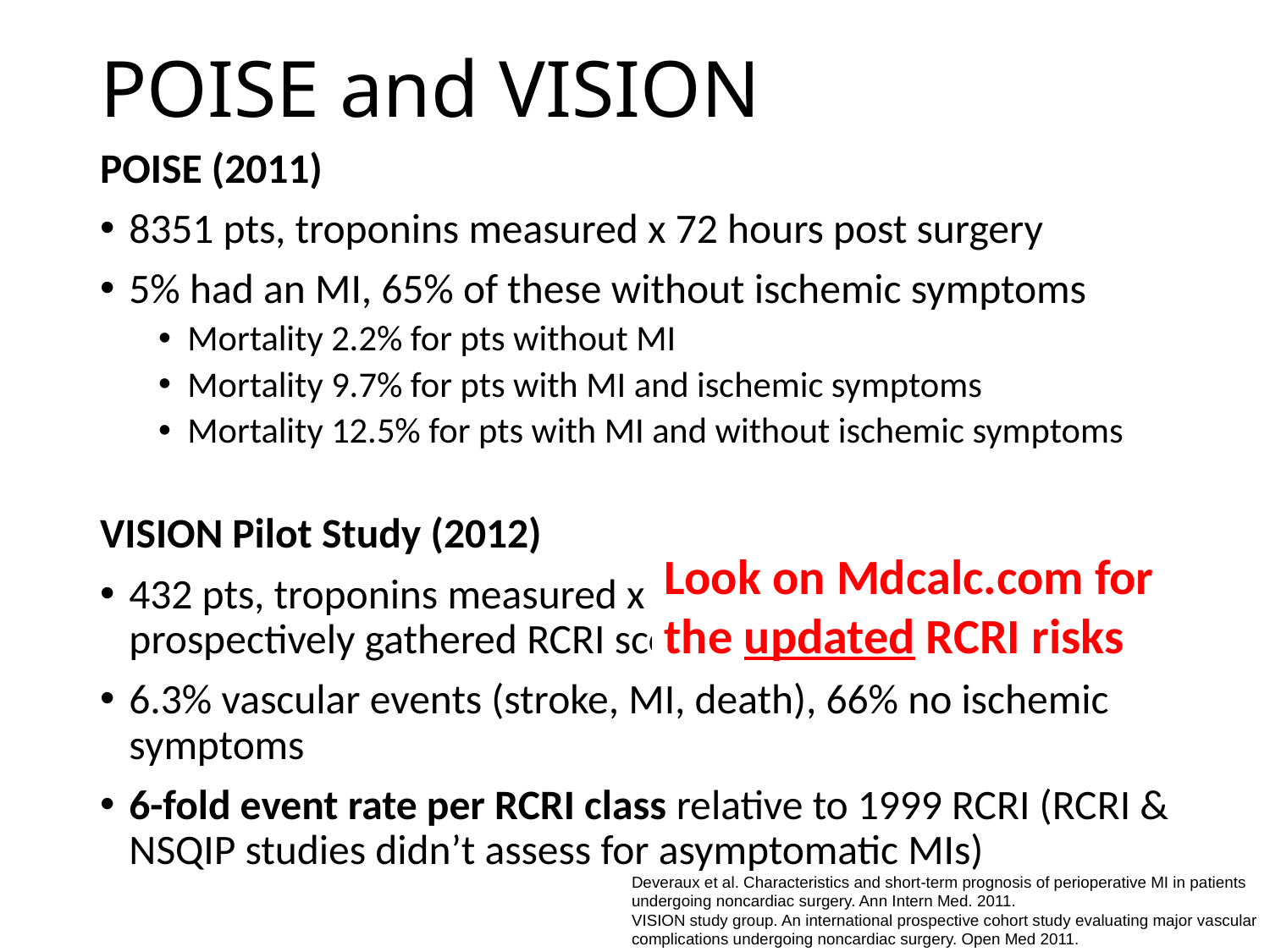

# POISE and VISION
POISE (2011)
8351 pts, troponins measured x 72 hours post surgery
5% had an MI, 65% of these without ischemic symptoms
Mortality 2.2% for pts without MI
Mortality 9.7% for pts with MI and ischemic symptoms
Mortality 12.5% for pts with MI and without ischemic symptoms
VISION Pilot Study (2012)
432 pts, troponins measured x 72 hours post surgery, prospectively gathered RCRI scores
6.3% vascular events (stroke, MI, death), 66% no ischemic symptoms
6-fold event rate per RCRI class relative to 1999 RCRI (RCRI & NSQIP studies didn’t assess for asymptomatic MIs)
Look on Mdcalc.com for the updated RCRI risks
Deveraux et al. Characteristics and short-term prognosis of perioperative MI in patients undergoing noncardiac surgery. Ann Intern Med. 2011.
VISION study group. An international prospective cohort study evaluating major vascular complications undergoing noncardiac surgery. Open Med 2011.

## Slide 13
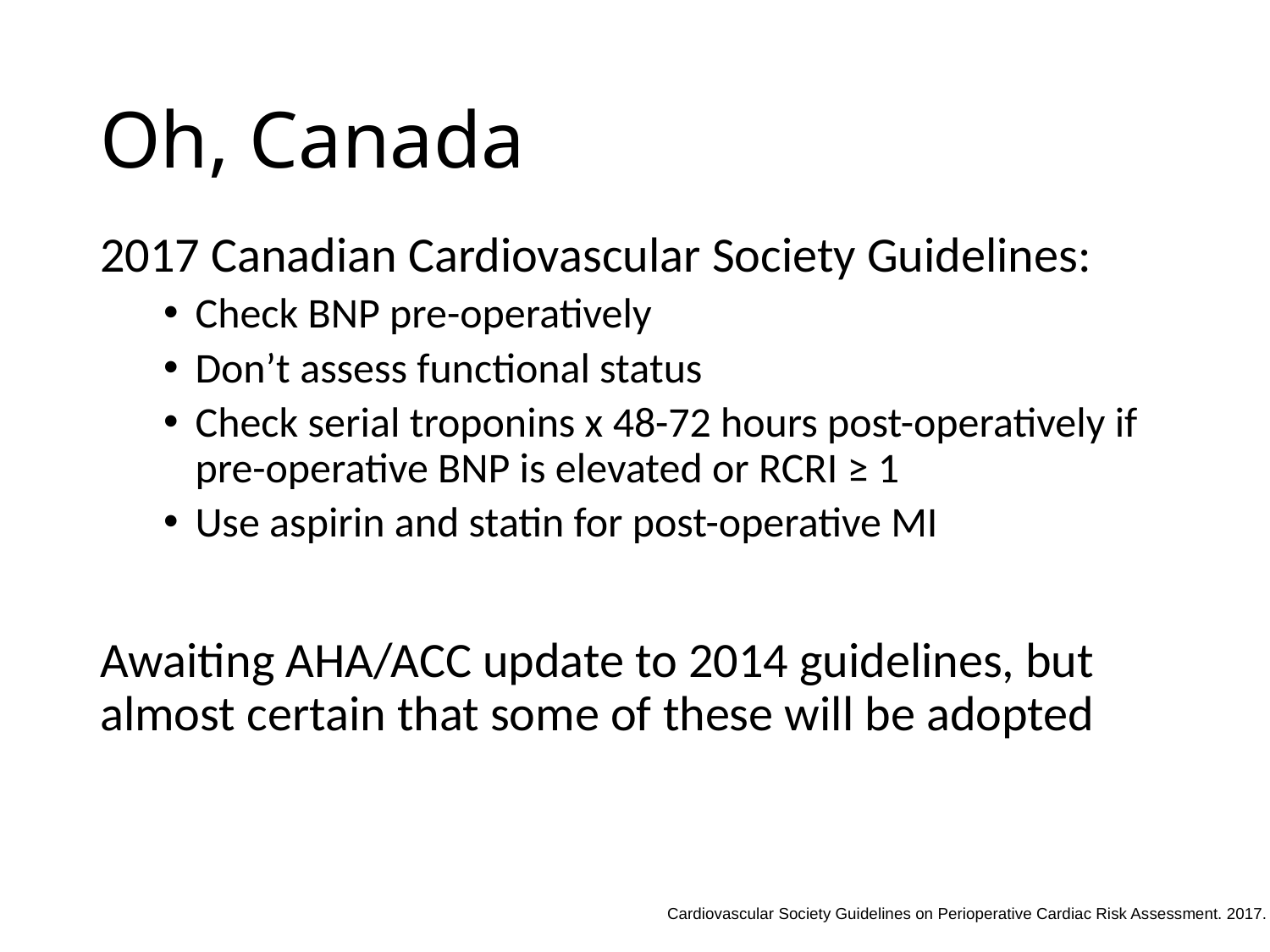

# Oh, Canada
2017 Canadian Cardiovascular Society Guidelines:
Check BNP pre-operatively
Don’t assess functional status
Check serial troponins x 48-72 hours post-operatively if pre-operative BNP is elevated or RCRI ≥ 1
Use aspirin and statin for post-operative MI
Awaiting AHA/ACC update to 2014 guidelines, but almost certain that some of these will be adopted
Cardiovascular Society Guidelines on Perioperative Cardiac Risk Assessment. 2017.

## Slide 14
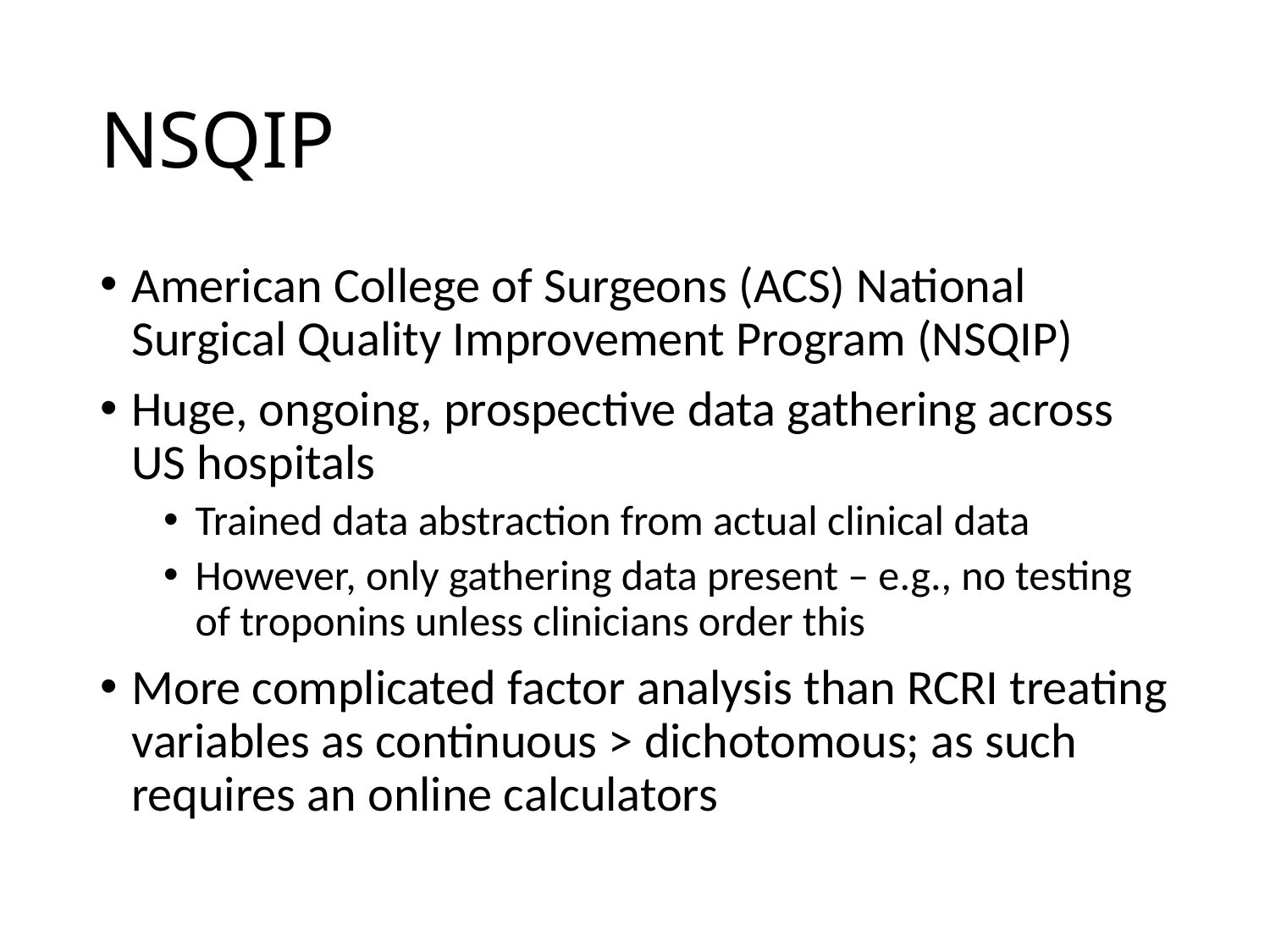

# NSQIP
American College of Surgeons (ACS) National Surgical Quality Improvement Program (NSQIP)
Huge, ongoing, prospective data gathering across US hospitals
Trained data abstraction from actual clinical data
However, only gathering data present – e.g., no testing of troponins unless clinicians order this
More complicated factor analysis than RCRI treating variables as continuous > dichotomous; as such requires an online calculators

## Slide 15
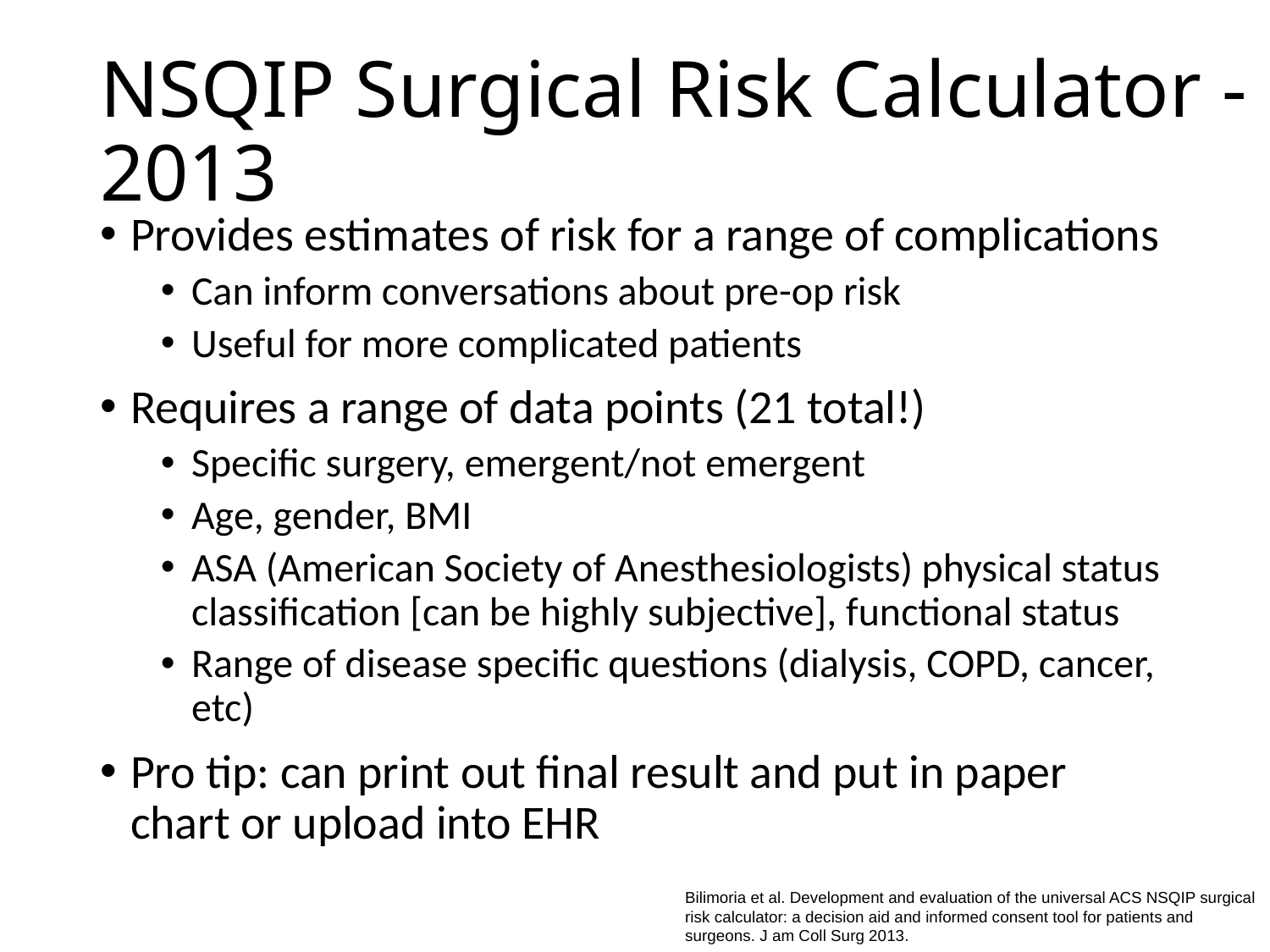

# NSQIP Surgical Risk Calculator - 2013
Provides estimates of risk for a range of complications
Can inform conversations about pre-op risk
Useful for more complicated patients
Requires a range of data points (21 total!)
Specific surgery, emergent/not emergent
Age, gender, BMI
ASA (American Society of Anesthesiologists) physical status classification [can be highly subjective], functional status
Range of disease specific questions (dialysis, COPD, cancer, etc)
Pro tip: can print out final result and put in paper chart or upload into EHR
Bilimoria et al. Development and evaluation of the universal ACS NSQIP surgical risk calculator: a decision aid and informed consent tool for patients and surgeons. J am Coll Surg 2013.

## Slide 16
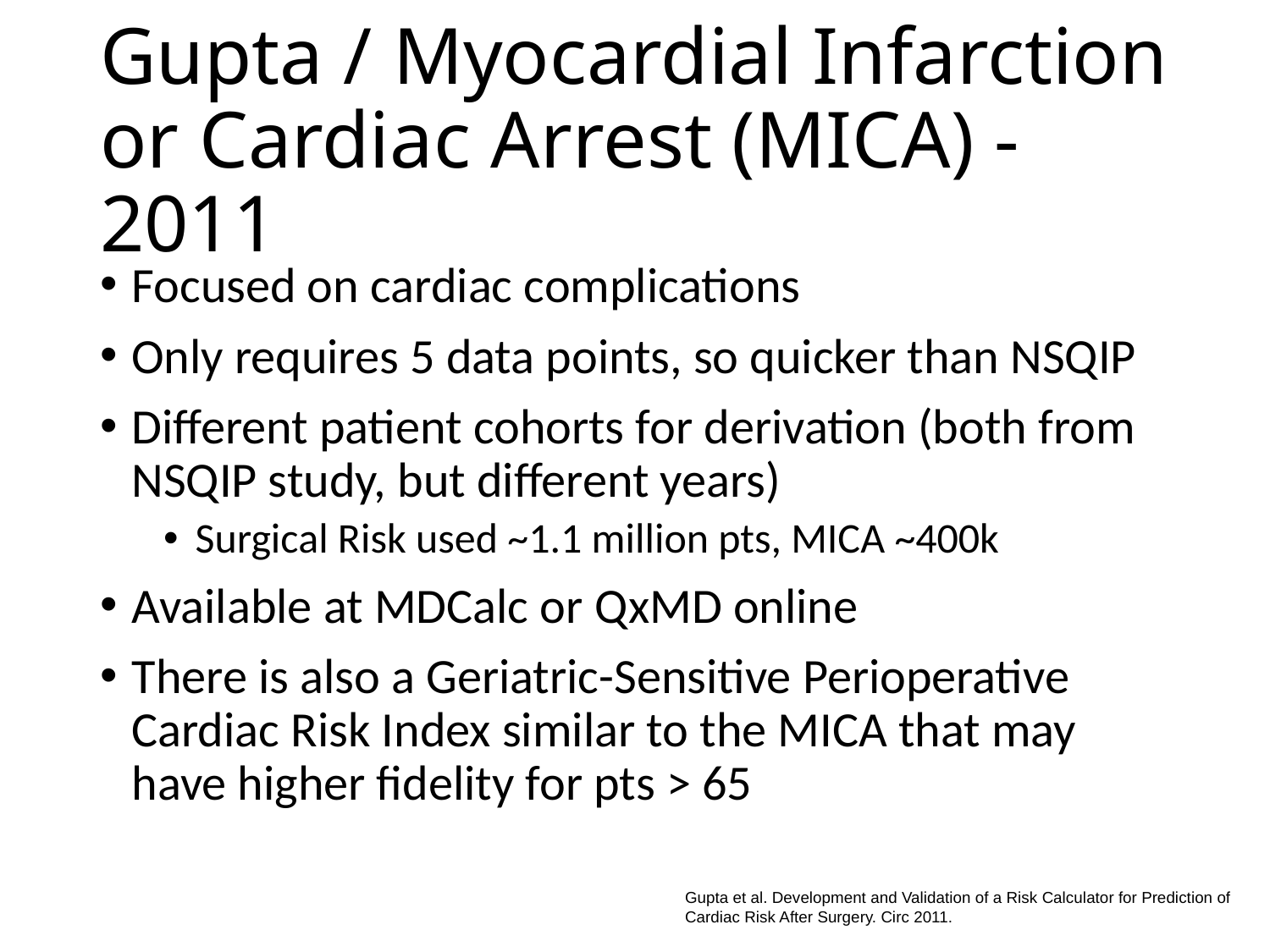

# Gupta / Myocardial Infarction or Cardiac Arrest (MICA) - 2011
Focused on cardiac complications
Only requires 5 data points, so quicker than NSQIP
Different patient cohorts for derivation (both from NSQIP study, but different years)
Surgical Risk used ~1.1 million pts, MICA ~400k
Available at MDCalc or QxMD online
There is also a Geriatric-Sensitive Perioperative Cardiac Risk Index similar to the MICA that may have higher fidelity for pts > 65
Gupta et al. Development and Validation of a Risk Calculator for Prediction of Cardiac Risk After Surgery. Circ 2011.

## Slide 17
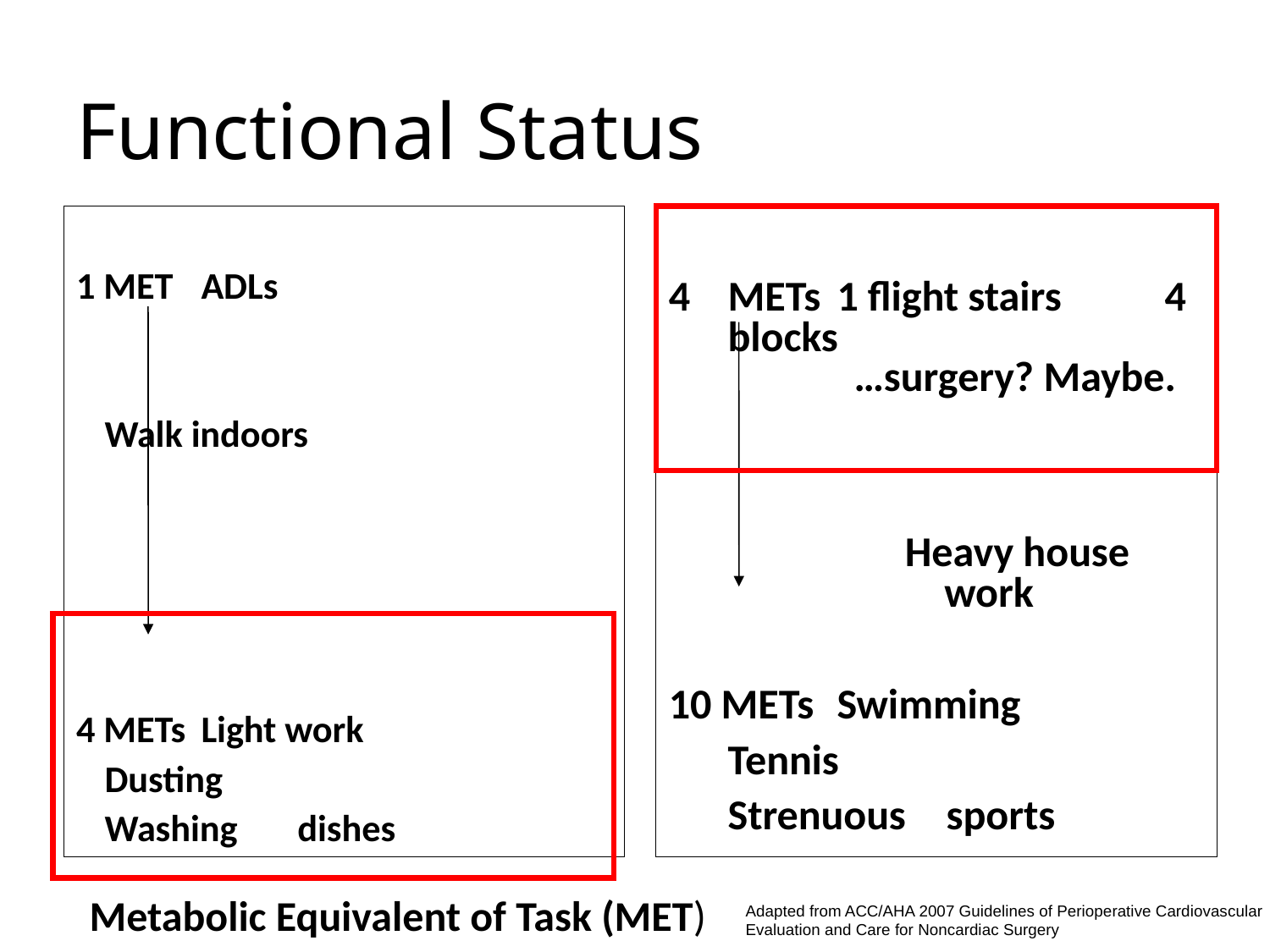

# Functional Status
METs	1 flight stairs 		4 blocks
Heavy house work
10 METs	Swimming
			Tennis
			Strenuous 			sports
1 MET		ADLs
			Walk indoors
4 METs		Light work
			Dusting
			Washing 			dishes
…surgery? Maybe.
Metabolic Equivalent of Task (MET)
Adapted from ACC/AHA 2007 Guidelines of Perioperative Cardiovascular Evaluation and Care for Noncardiac Surgery

## Slide 18
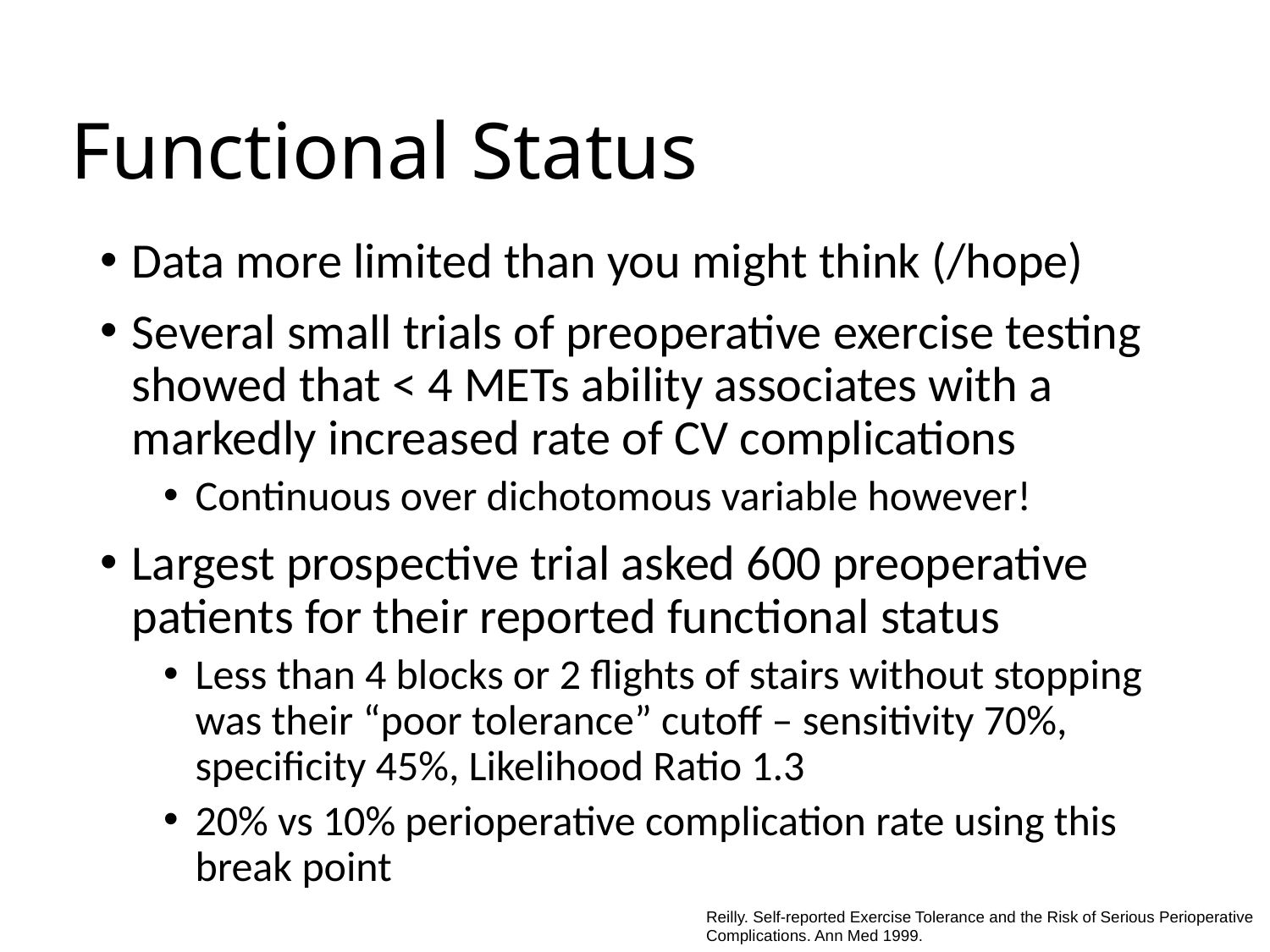

# Functional Status
Data more limited than you might think (/hope)
Several small trials of preoperative exercise testing showed that < 4 METs ability associates with a markedly increased rate of CV complications
Continuous over dichotomous variable however!
Largest prospective trial asked 600 preoperative patients for their reported functional status
Less than 4 blocks or 2 flights of stairs without stopping was their “poor tolerance” cutoff – sensitivity 70%, specificity 45%, Likelihood Ratio 1.3
20% vs 10% perioperative complication rate using this break point
Reilly. Self-reported Exercise Tolerance and the Risk of Serious Perioperative Complications. Ann Med 1999.

## Slide 19
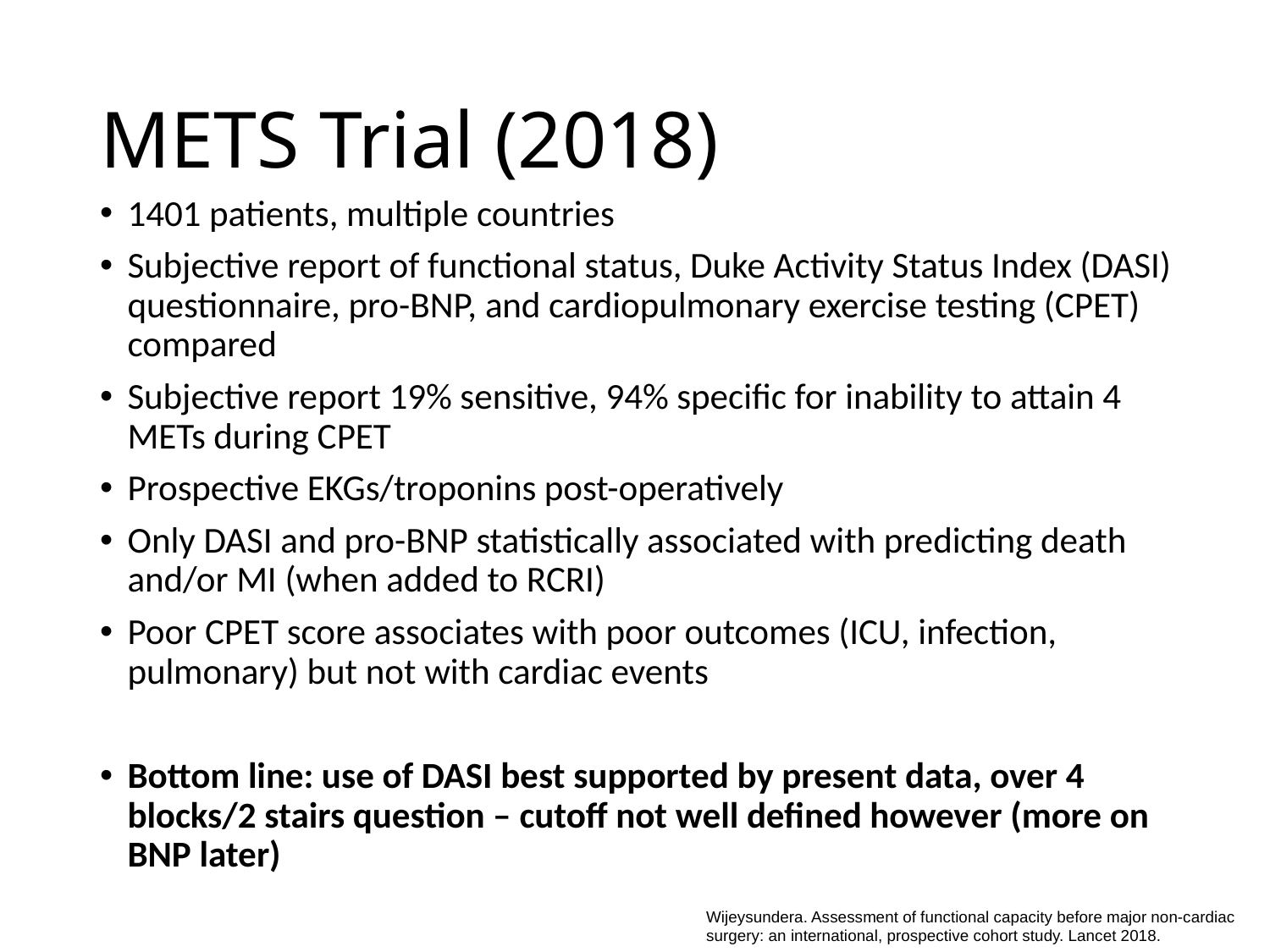

# METS Trial (2018)
1401 patients, multiple countries
Subjective report of functional status, Duke Activity Status Index (DASI) questionnaire, pro-BNP, and cardiopulmonary exercise testing (CPET) compared
Subjective report 19% sensitive, 94% specific for inability to attain 4 METs during CPET
Prospective EKGs/troponins post-operatively
Only DASI and pro-BNP statistically associated with predicting death and/or MI (when added to RCRI)
Poor CPET score associates with poor outcomes (ICU, infection, pulmonary) but not with cardiac events
Bottom line: use of DASI best supported by present data, over 4 blocks/2 stairs question – cutoff not well defined however (more on BNP later)
Wijeysundera. Assessment of functional capacity before major non-cardiac surgery: an international, prospective cohort study. Lancet 2018.

## Slide 20
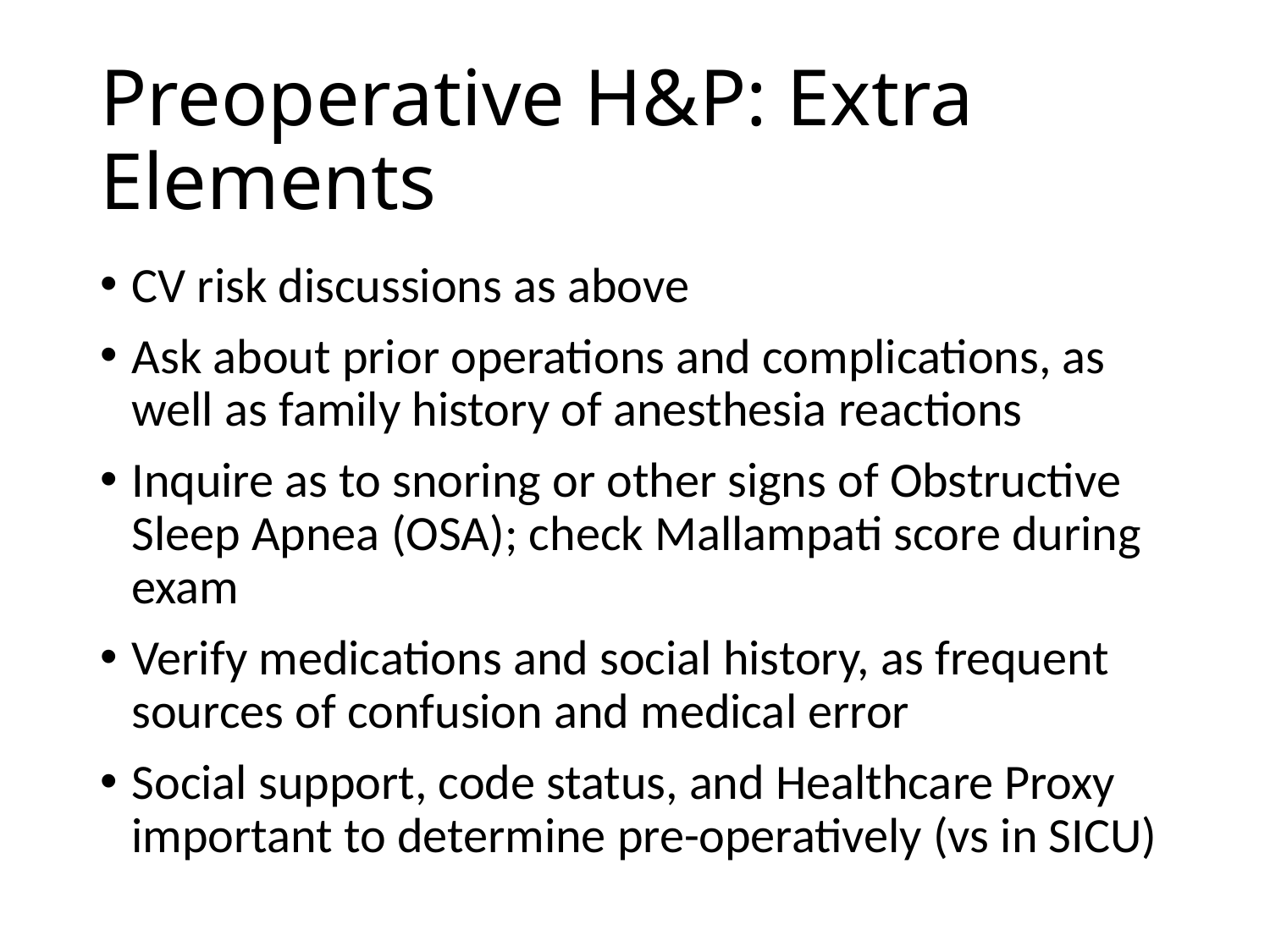

# Preoperative H&P: Extra Elements
CV risk discussions as above
Ask about prior operations and complications, as well as family history of anesthesia reactions
Inquire as to snoring or other signs of Obstructive Sleep Apnea (OSA); check Mallampati score during exam
Verify medications and social history, as frequent sources of confusion and medical error
Social support, code status, and Healthcare Proxy important to determine pre-operatively (vs in SICU)

## Slide 21
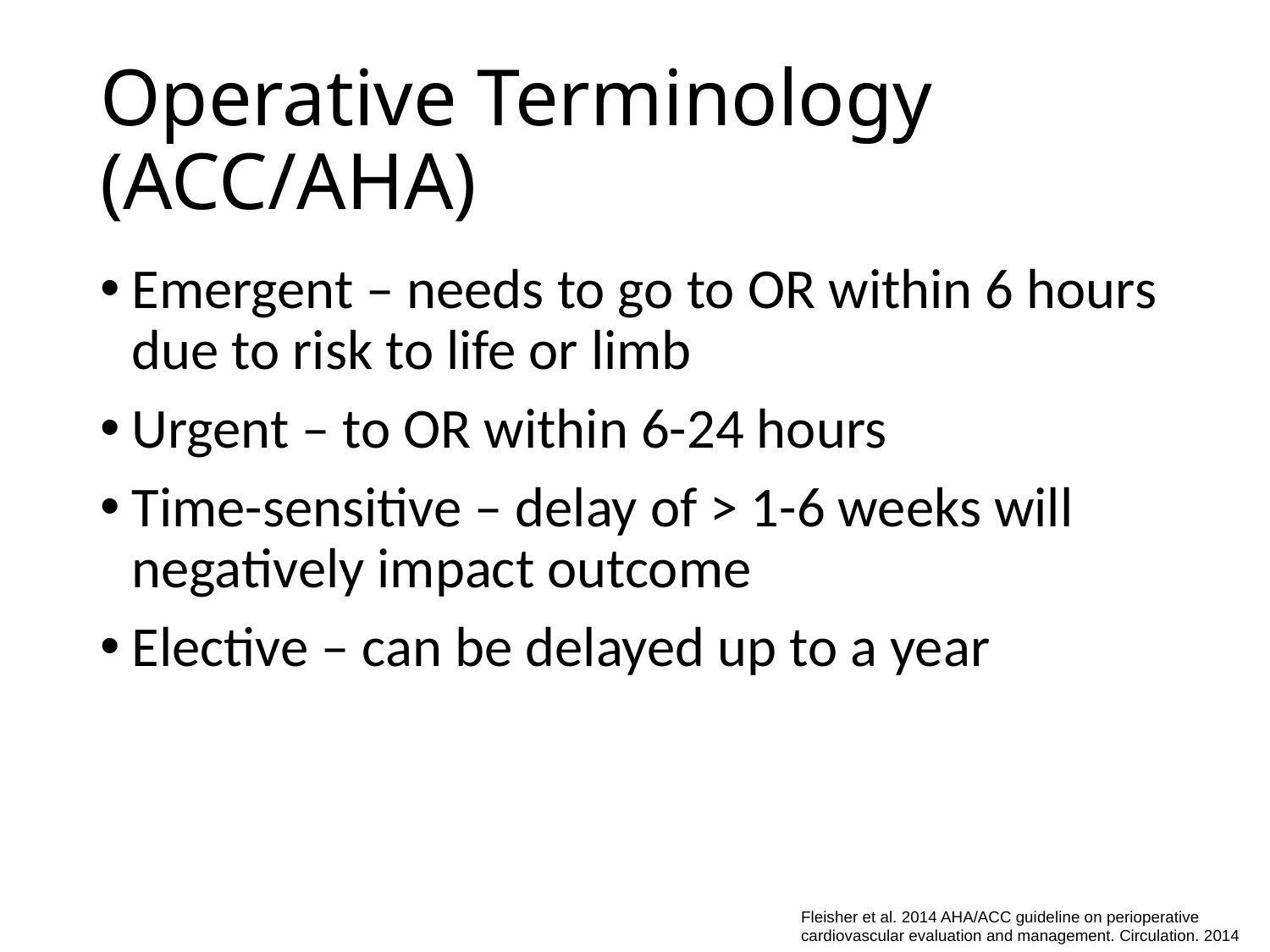

# Operative Terminology (ACC/AHA)
Emergent – needs to go to OR within 6 hours due to risk to life or limb
Urgent – to OR within 6-24 hours
Time-sensitive – delay of > 1-6 weeks will negatively impact outcome
Elective – can be delayed up to a year
Fleisher et al. 2014 AHA/ACC guideline on perioperative cardiovascular evaluation and management. Circulation. 2014

## Slide 22
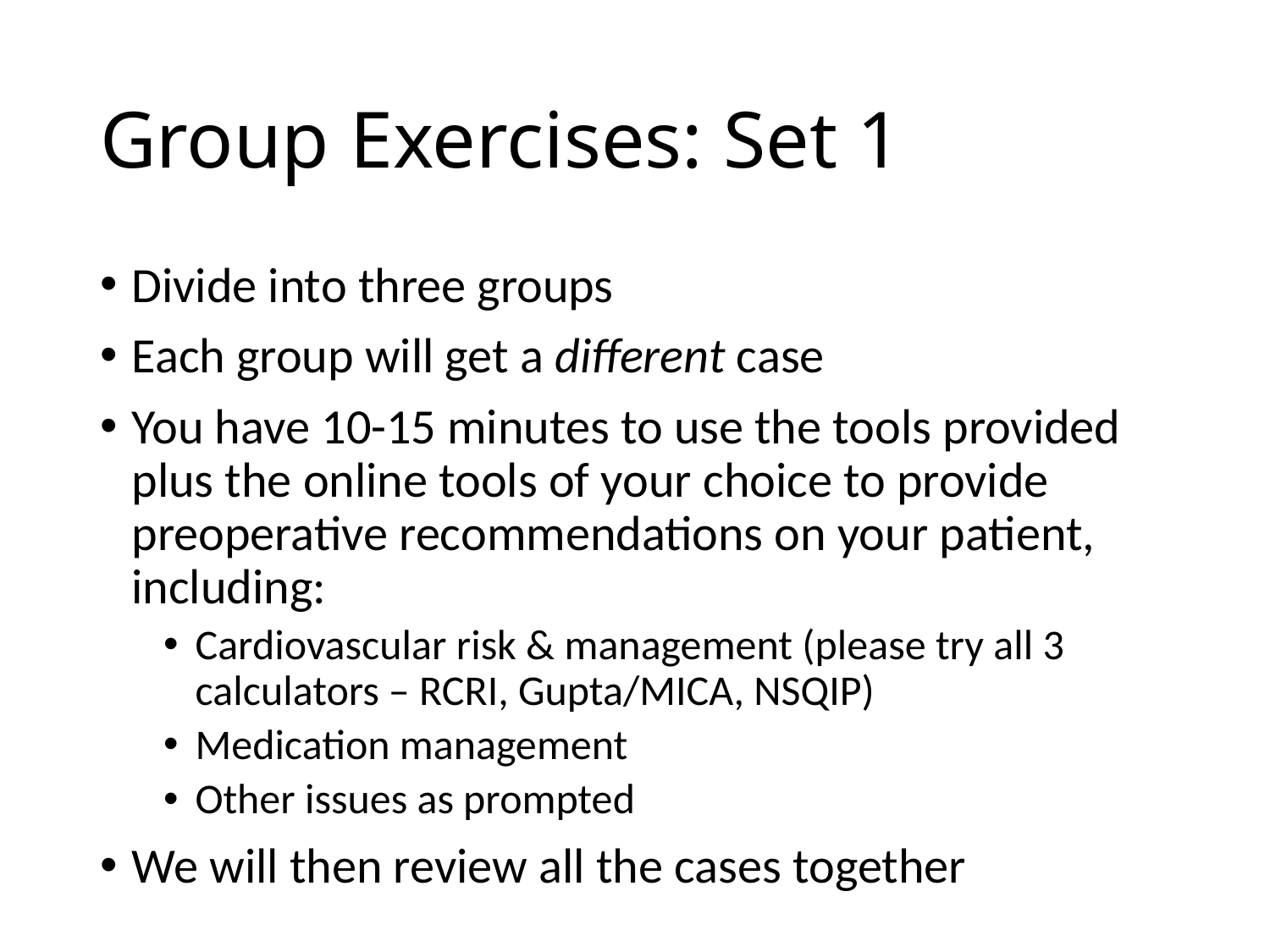

# Group Exercises: Set 1
Divide into three groups
Each group will get a different case
You have 10-15 minutes to use the tools provided plus the online tools of your choice to provide preoperative recommendations on your patient, including:
Cardiovascular risk & management (please try all 3 calculators – RCRI, Gupta/MICA, NSQIP)
Medication management
Other issues as prompted
We will then review all the cases together

## Slide 23
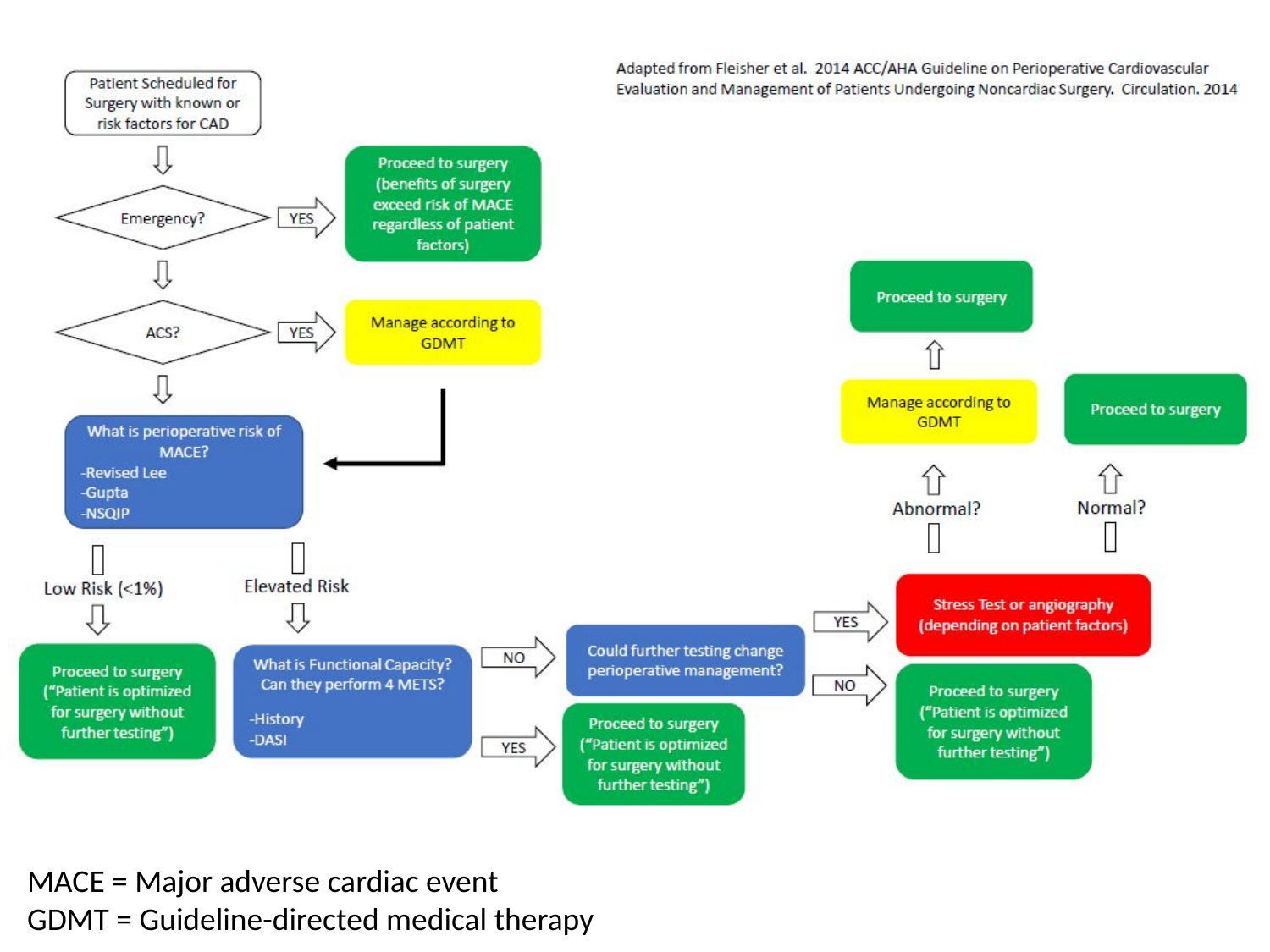

#
MACE = Major adverse cardiac event
GDMT = Guideline-directed medical therapy

## Slide 24
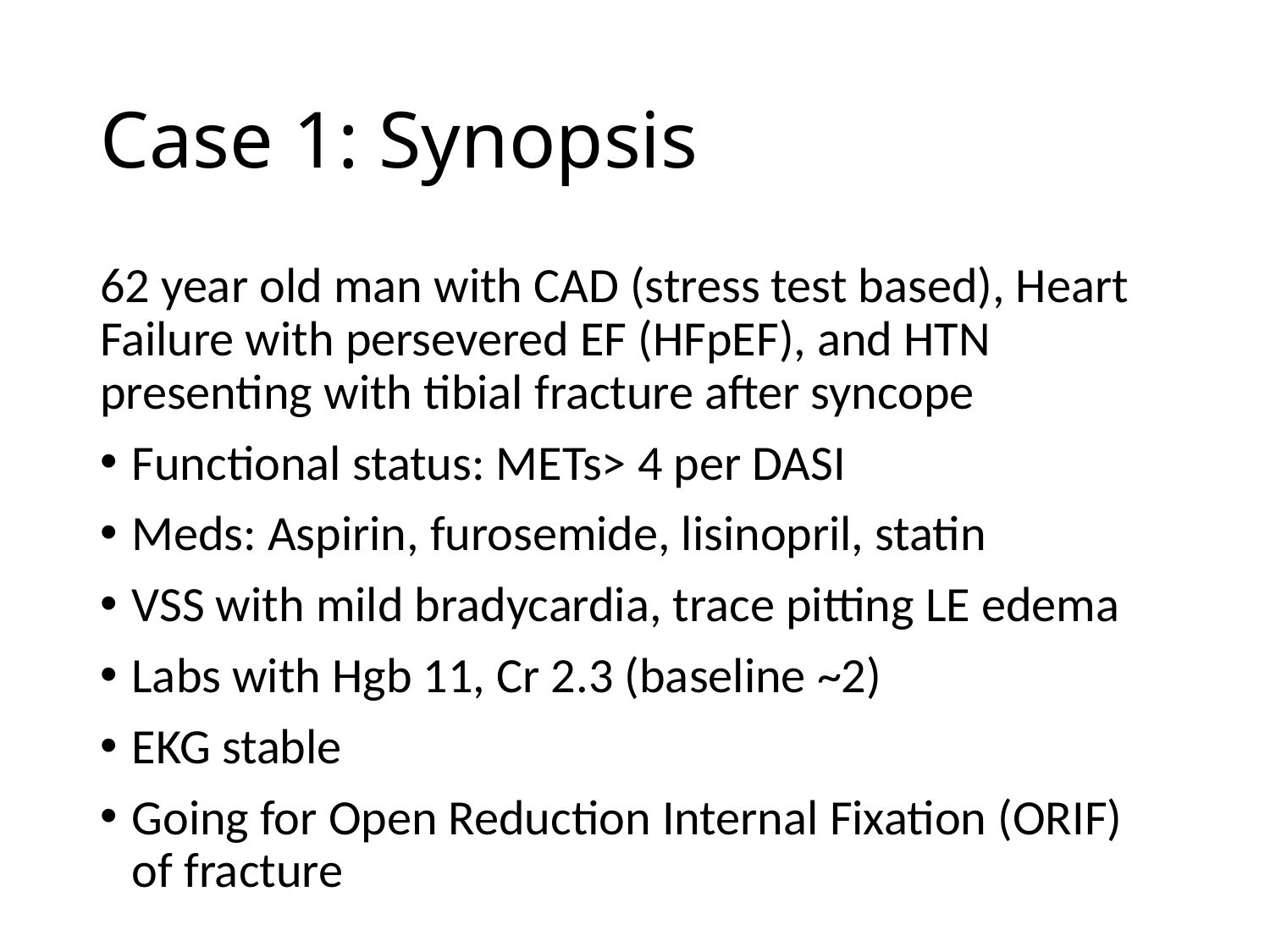

# Case 1: Synopsis
62 year old man with CAD (stress test based), Heart Failure with persevered EF (HFpEF), and HTN presenting with tibial fracture after syncope
Functional status: METs> 4 per DASI
Meds: Aspirin, furosemide, lisinopril, statin
VSS with mild bradycardia, trace pitting LE edema
Labs with Hgb 11, Cr 2.3 (baseline ~2)
EKG stable
Going for Open Reduction Internal Fixation (ORIF) of fracture

## Slide 25
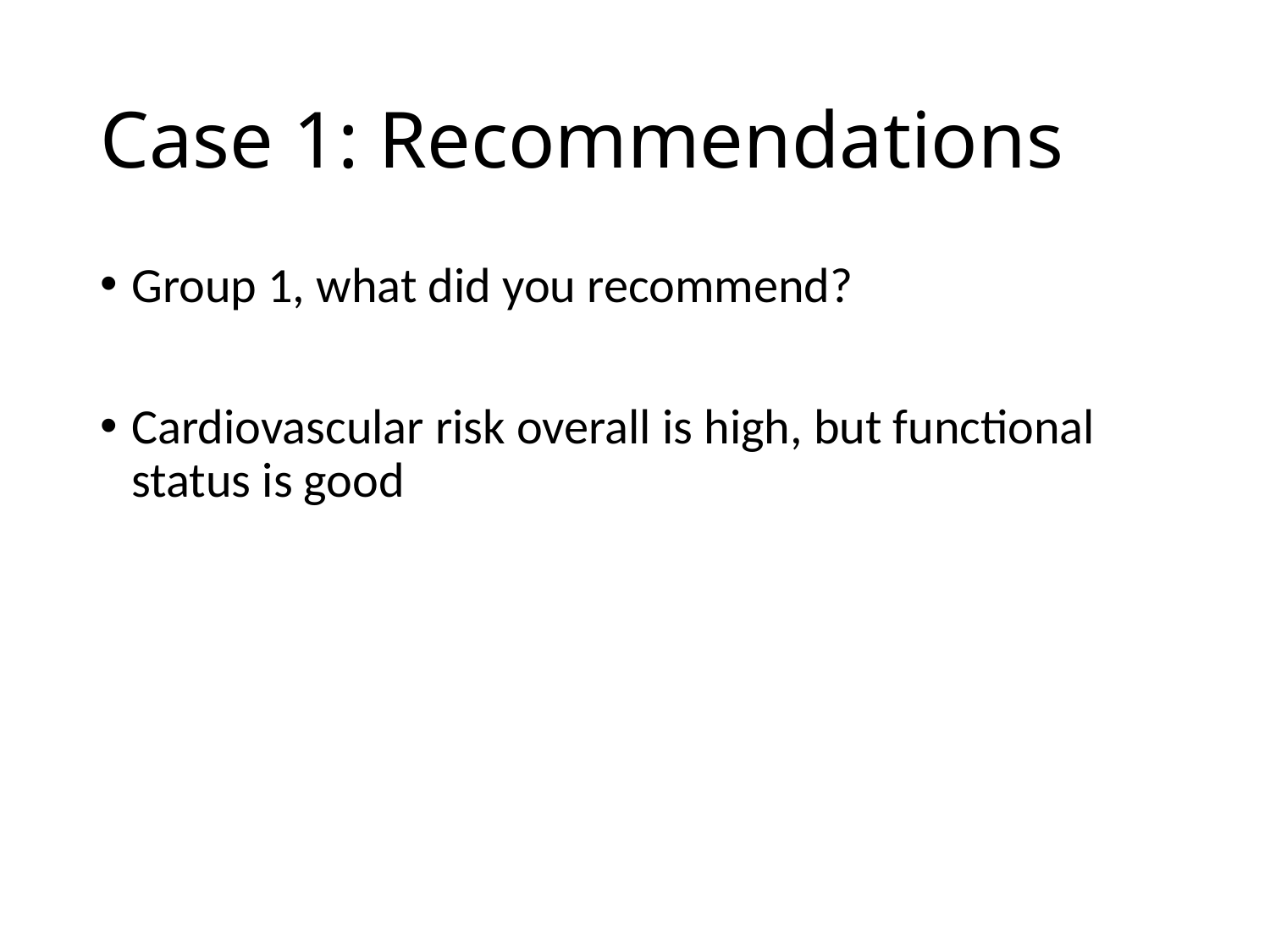

# Case 1: Recommendations
Group 1, what did you recommend?
Cardiovascular risk overall is high, but functional status is good

## Slide 26
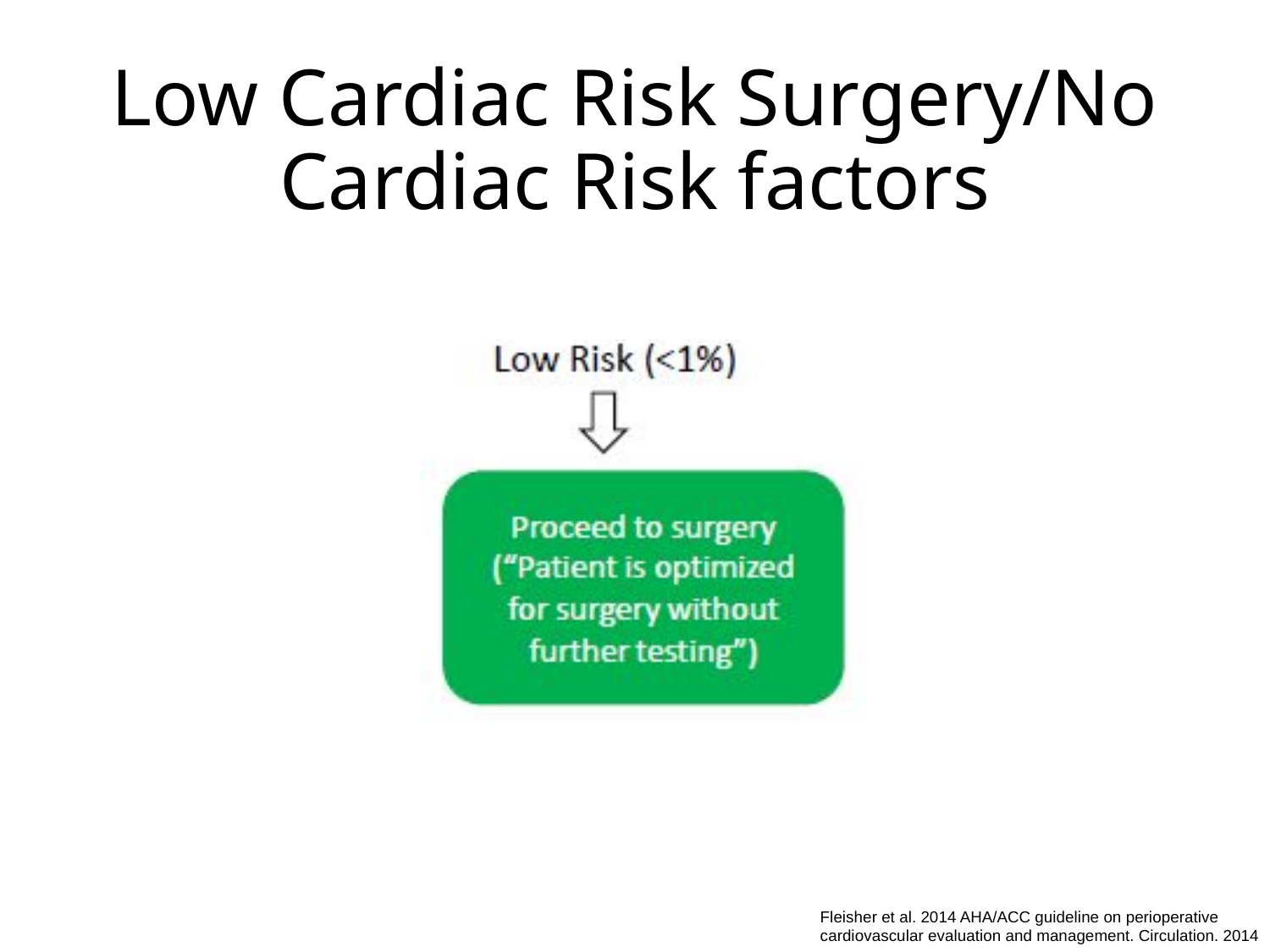

# Low Cardiac Risk Surgery/No Cardiac Risk factors
Fleisher et al. 2014 AHA/ACC guideline on perioperative cardiovascular evaluation and management. Circulation. 2014

## Slide 27
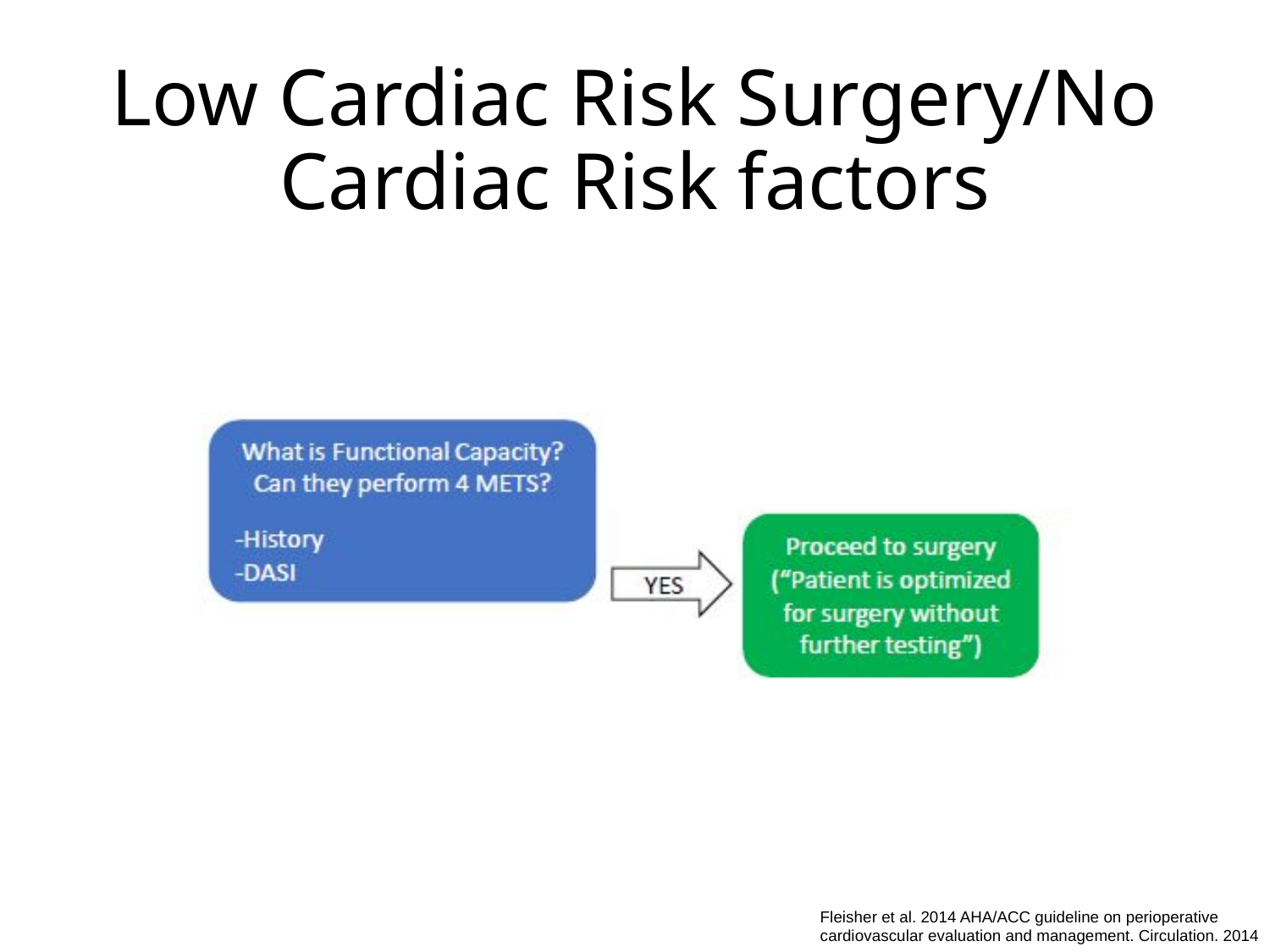

# Low Cardiac Risk Surgery/No Cardiac Risk factors
Fleisher et al. 2014 AHA/ACC guideline on perioperative cardiovascular evaluation and management. Circulation. 2014

## Slide 28
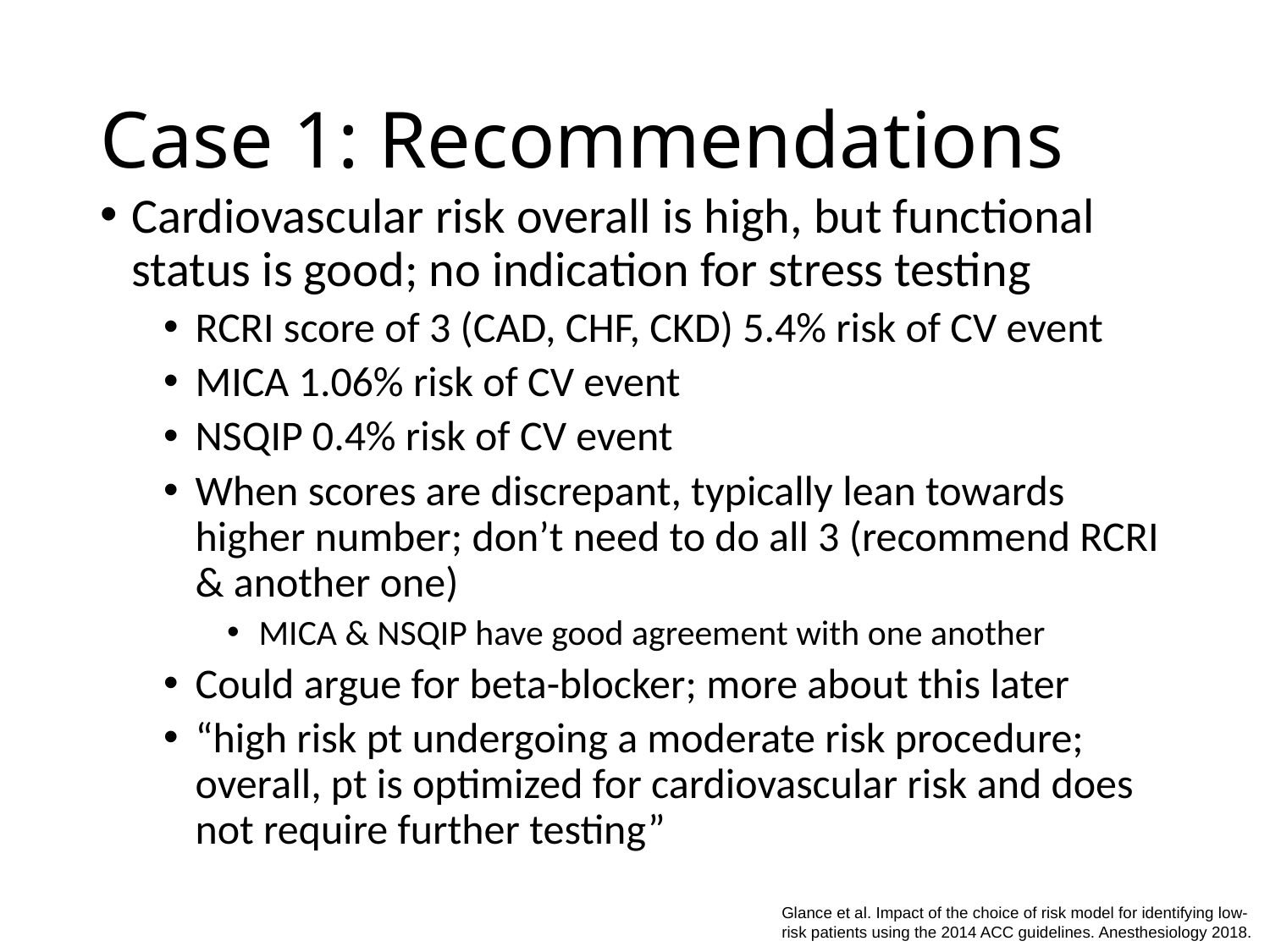

# Case 1: Recommendations
Cardiovascular risk overall is high, but functional status is good; no indication for stress testing
RCRI score of 3 (CAD, CHF, CKD) 5.4% risk of CV event
MICA 1.06% risk of CV event
NSQIP 0.4% risk of CV event
When scores are discrepant, typically lean towards higher number; don’t need to do all 3 (recommend RCRI & another one)
MICA & NSQIP have good agreement with one another
Could argue for beta-blocker; more about this later
“high risk pt undergoing a moderate risk procedure; overall, pt is optimized for cardiovascular risk and does not require further testing”
Glance et al. Impact of the choice of risk model for identifying low-risk patients using the 2014 ACC guidelines. Anesthesiology 2018.

## Slide 29
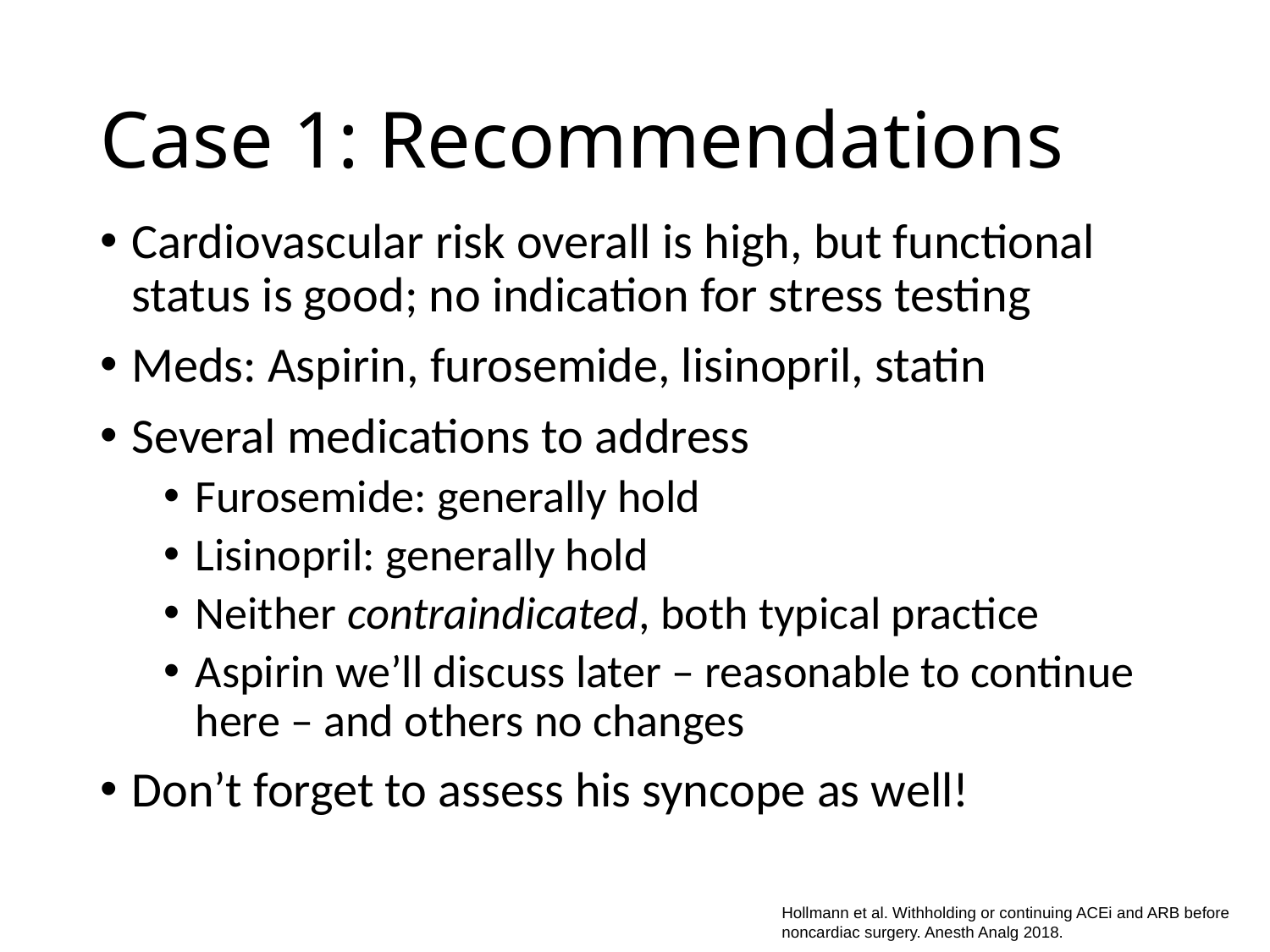

# Case 1: Recommendations
Cardiovascular risk overall is high, but functional status is good; no indication for stress testing
Meds: Aspirin, furosemide, lisinopril, statin
Several medications to address
Furosemide: generally hold
Lisinopril: generally hold
Neither contraindicated, both typical practice
Aspirin we’ll discuss later – reasonable to continue here – and others no changes
Don’t forget to assess his syncope as well!
Hollmann et al. Withholding or continuing ACEi and ARB before noncardiac surgery. Anesth Analg 2018.

## Slide 30
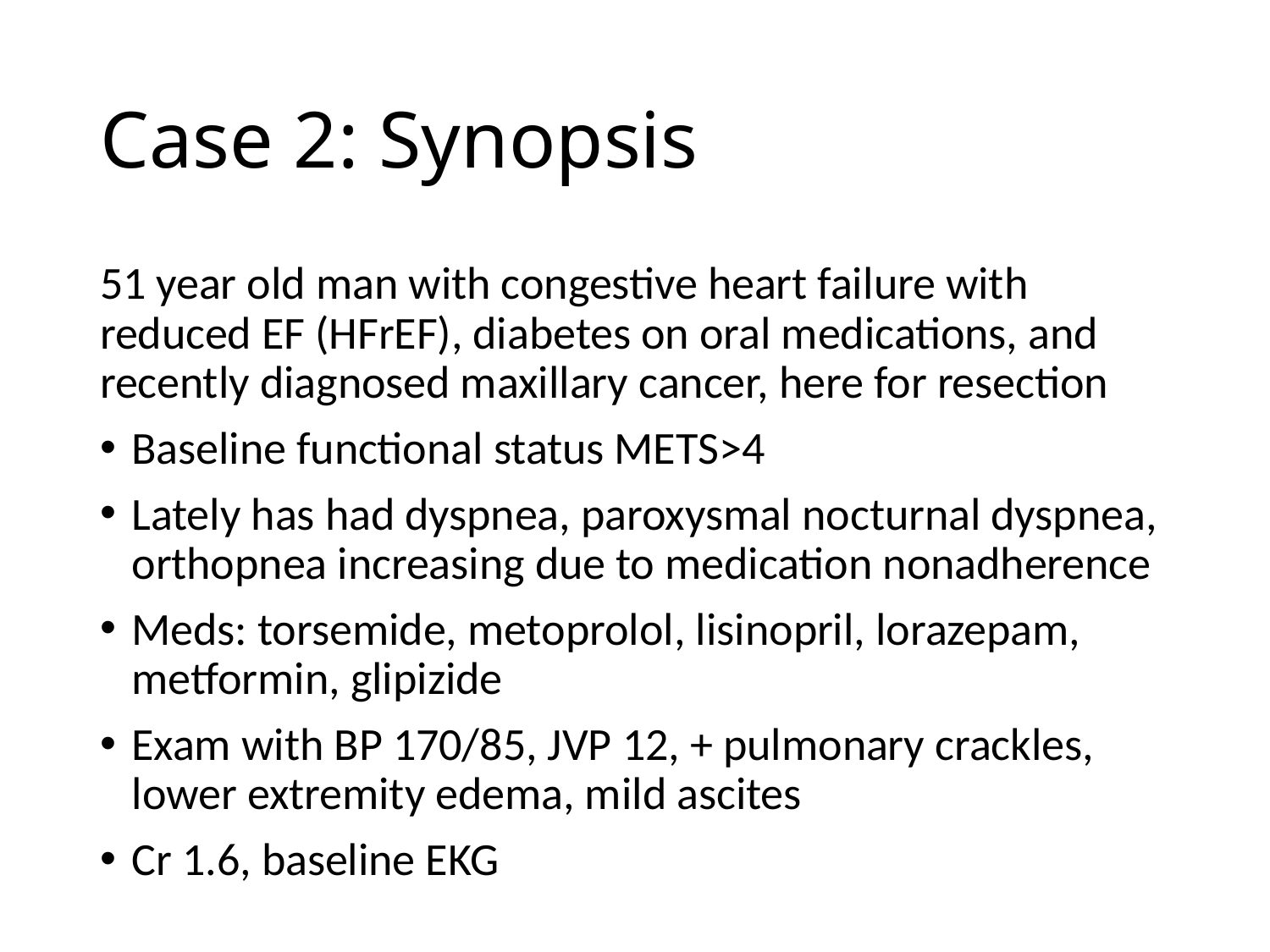

# Case 2: Synopsis
51 year old man with congestive heart failure with reduced EF (HFrEF), diabetes on oral medications, and recently diagnosed maxillary cancer, here for resection
Baseline functional status METS>4
Lately has had dyspnea, paroxysmal nocturnal dyspnea, orthopnea increasing due to medication nonadherence
Meds: torsemide, metoprolol, lisinopril, lorazepam, metformin, glipizide
Exam with BP 170/85, JVP 12, + pulmonary crackles, lower extremity edema, mild ascites
Cr 1.6, baseline EKG

## Slide 31
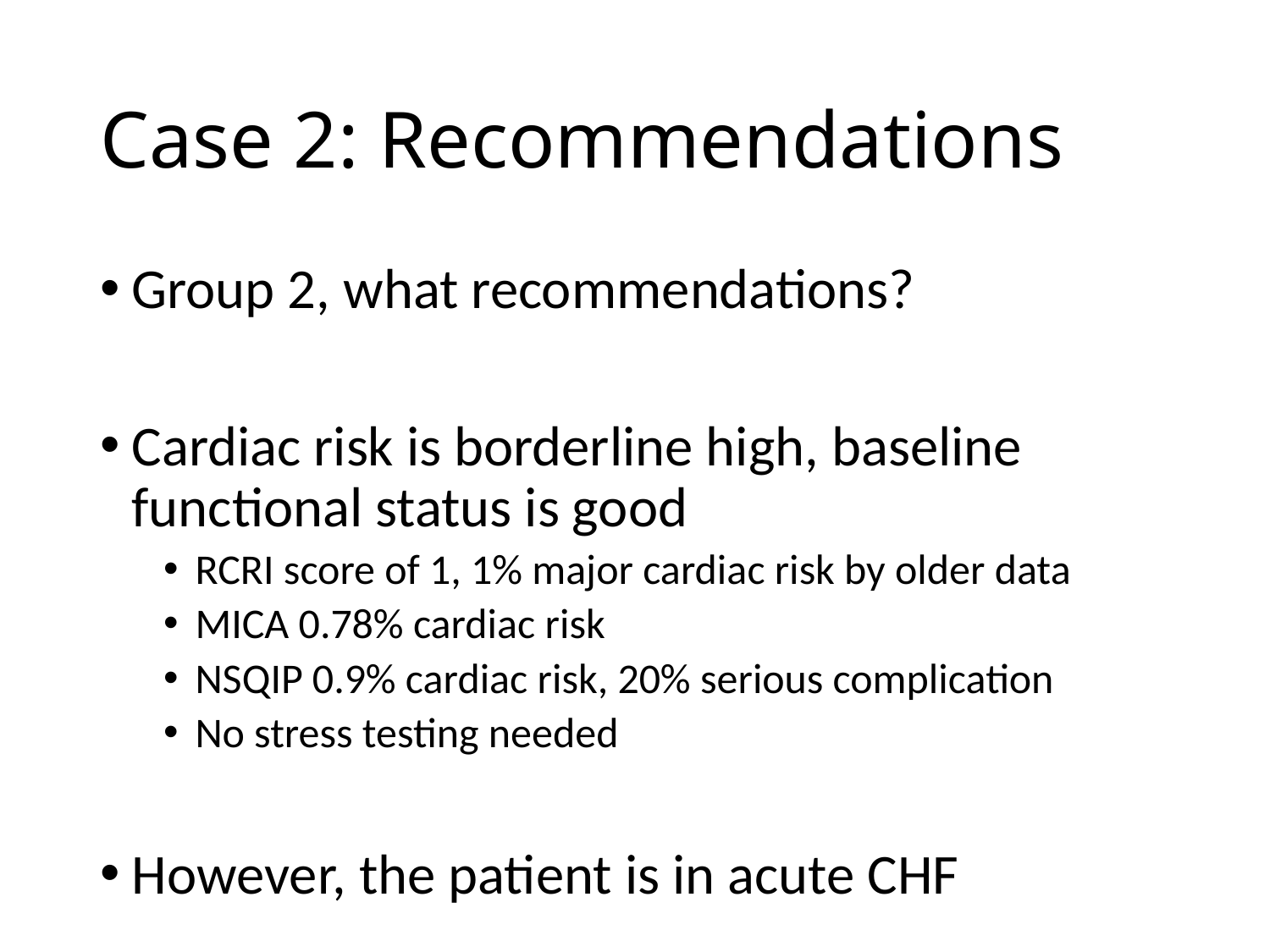

# Case 2: Recommendations
Group 2, what recommendations?
Cardiac risk is borderline high, baseline functional status is good
RCRI score of 1, 1% major cardiac risk by older data
MICA 0.78% cardiac risk
NSQIP 0.9% cardiac risk, 20% serious complication
No stress testing needed
However, the patient is in acute CHF

## Slide 32
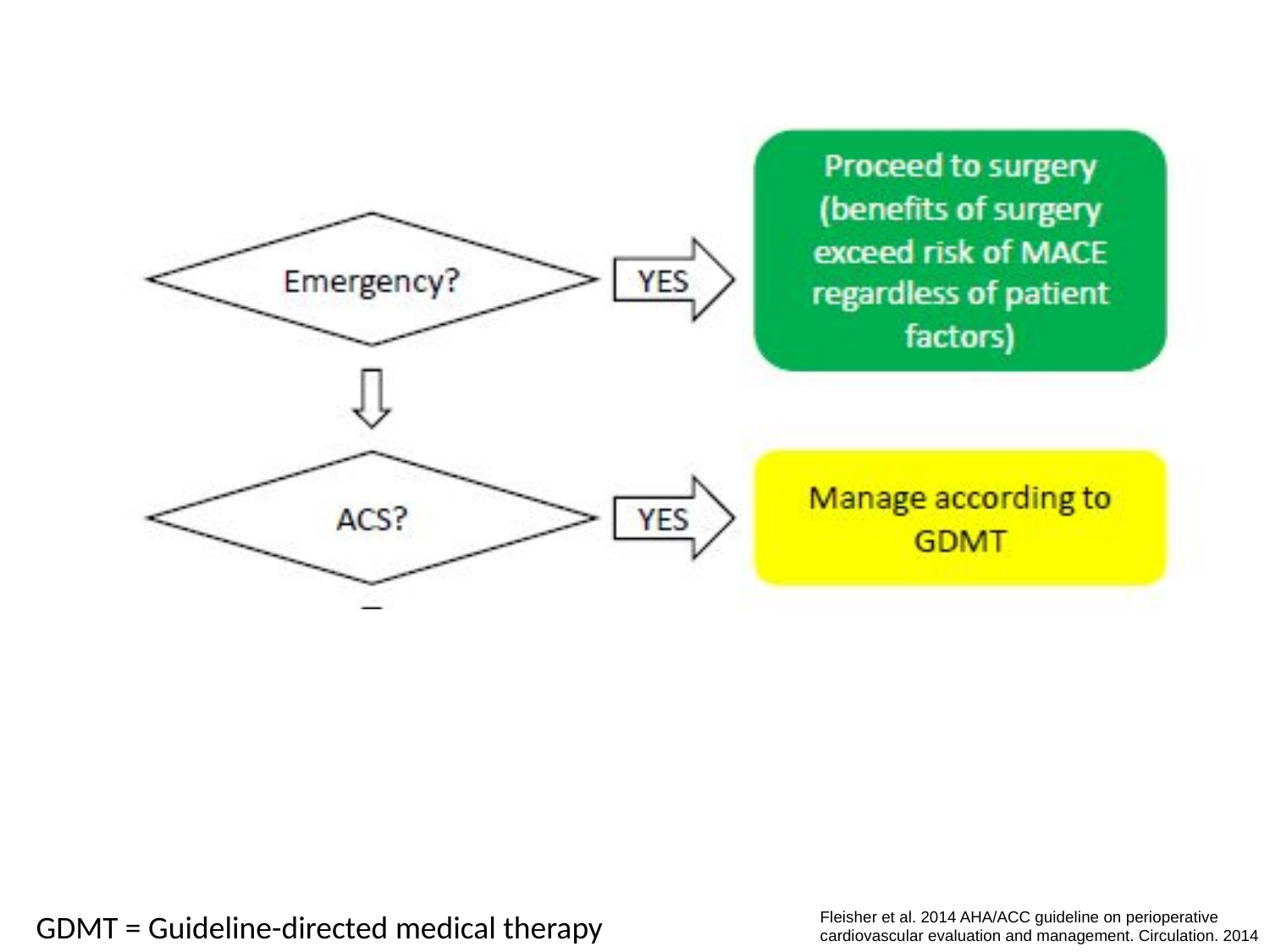

GDMT = Guideline-directed medical therapy
Fleisher et al. 2014 AHA/ACC guideline on perioperative cardiovascular evaluation and management. Circulation. 2014

## Slide 33
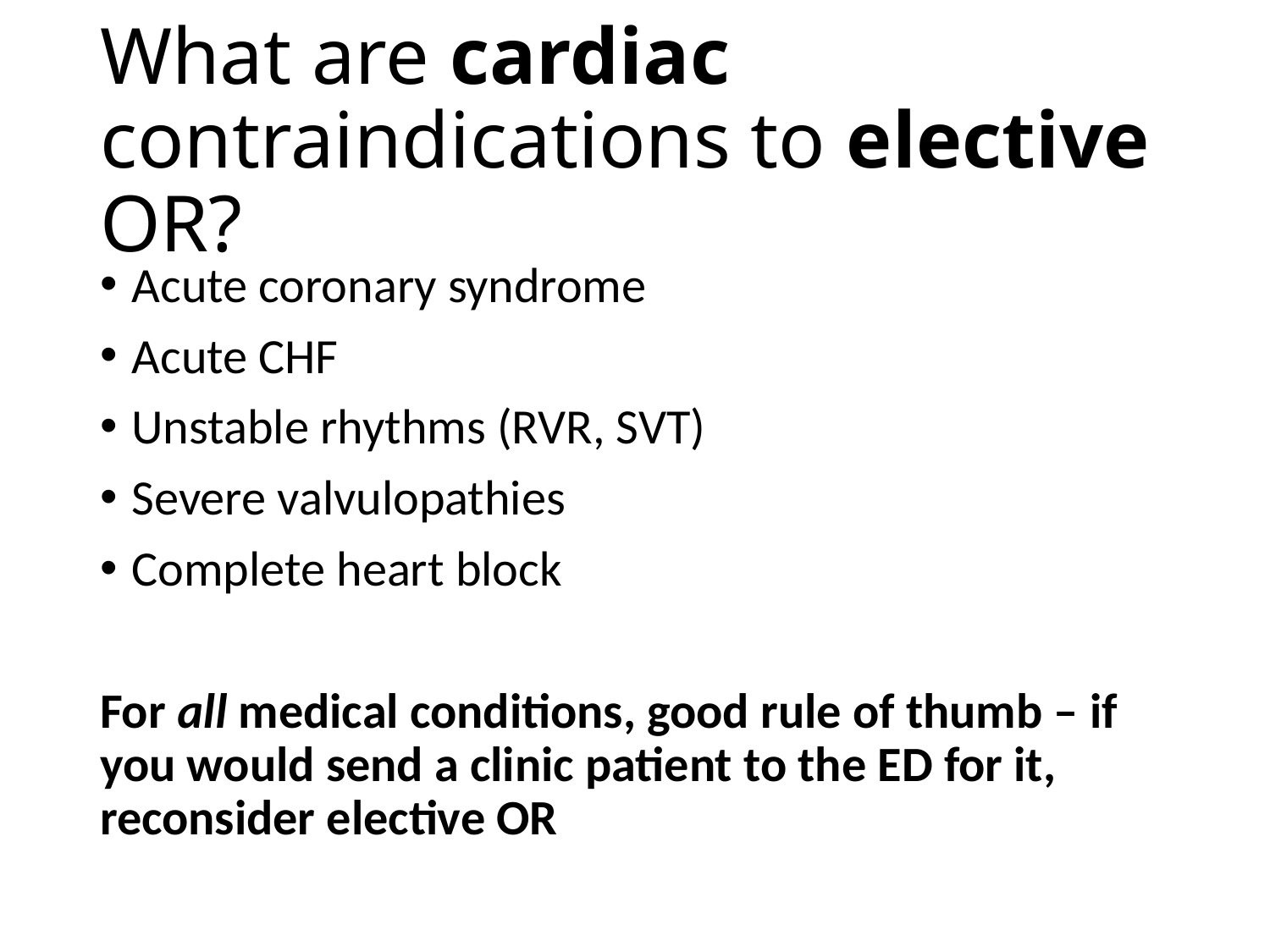

# What are cardiac contraindications to elective OR?
Acute coronary syndrome
Acute CHF
Unstable rhythms (RVR, SVT)
Severe valvulopathies
Complete heart block
For all medical conditions, good rule of thumb – if you would send a clinic patient to the ED for it, reconsider elective OR

## Slide 34
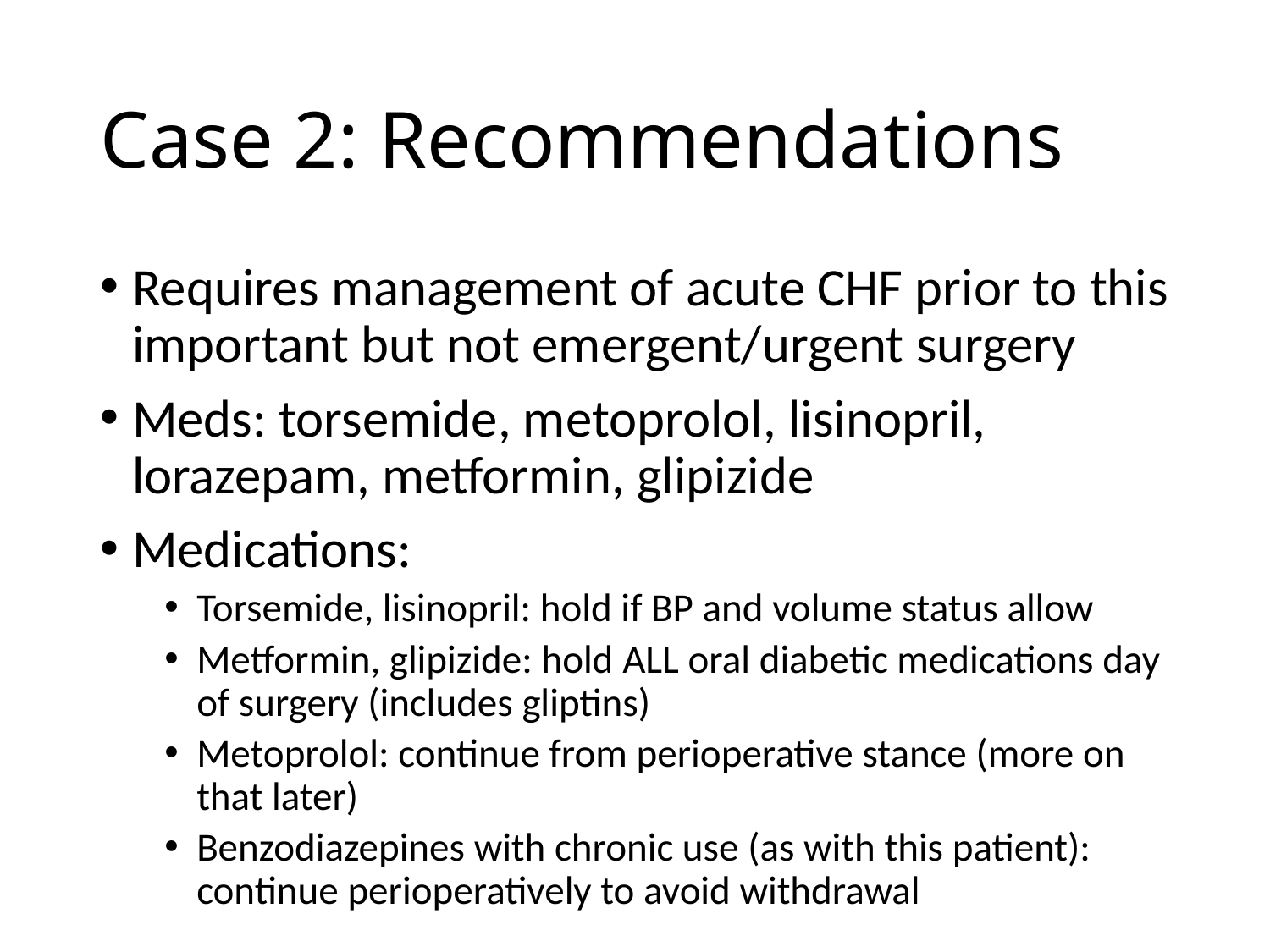

# Case 2: Recommendations
Requires management of acute CHF prior to this important but not emergent/urgent surgery
Meds: torsemide, metoprolol, lisinopril, lorazepam, metformin, glipizide
Medications:
Torsemide, lisinopril: hold if BP and volume status allow
Metformin, glipizide: hold ALL oral diabetic medications day of surgery (includes gliptins)
Metoprolol: continue from perioperative stance (more on that later)
Benzodiazepines with chronic use (as with this patient): continue perioperatively to avoid withdrawal

## Slide 35
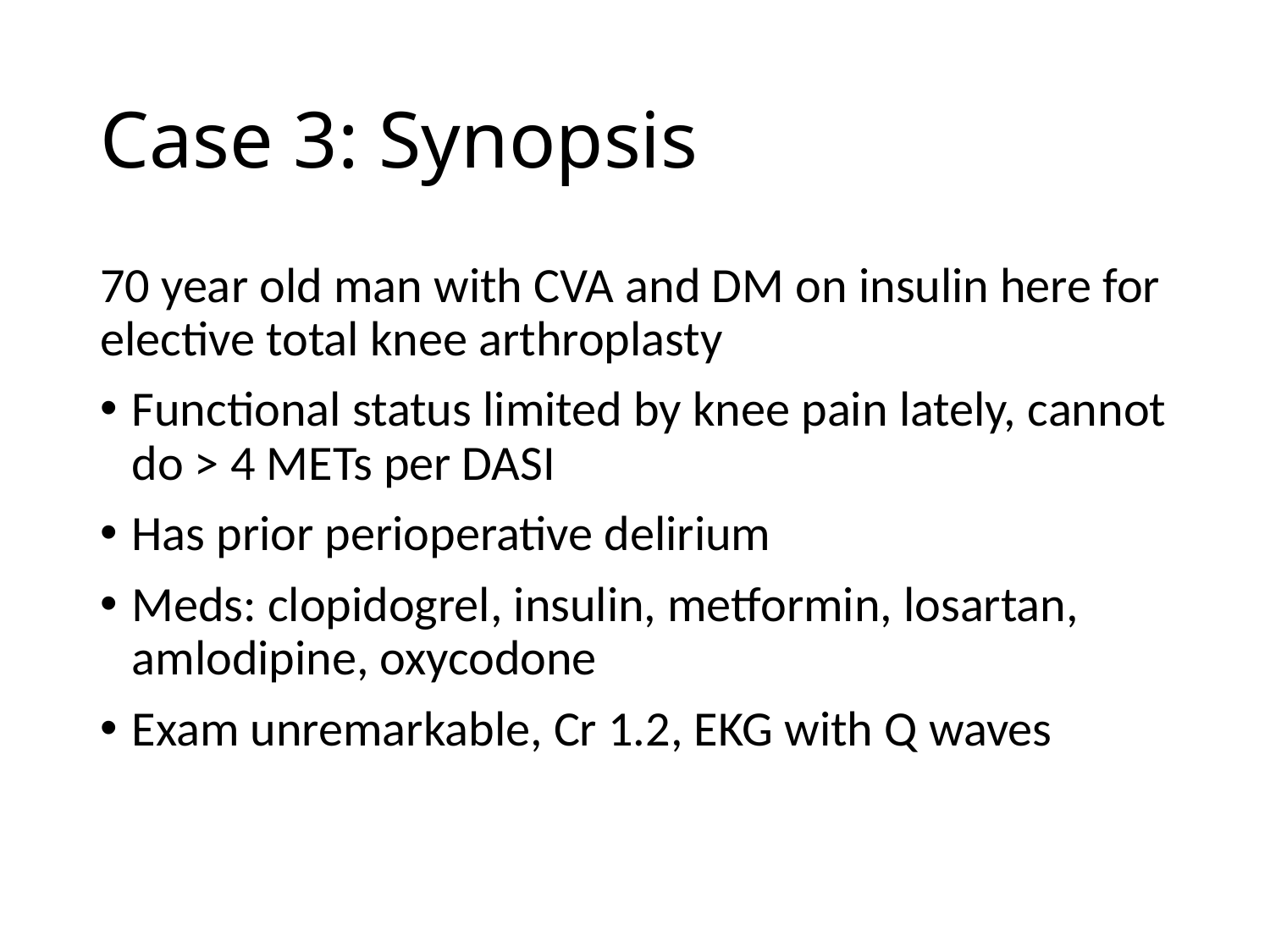

# Case 3: Synopsis
70 year old man with CVA and DM on insulin here for elective total knee arthroplasty
Functional status limited by knee pain lately, cannot do > 4 METs per DASI
Has prior perioperative delirium
Meds: clopidogrel, insulin, metformin, losartan, amlodipine, oxycodone
Exam unremarkable, Cr 1.2, EKG with Q waves

## Slide 36
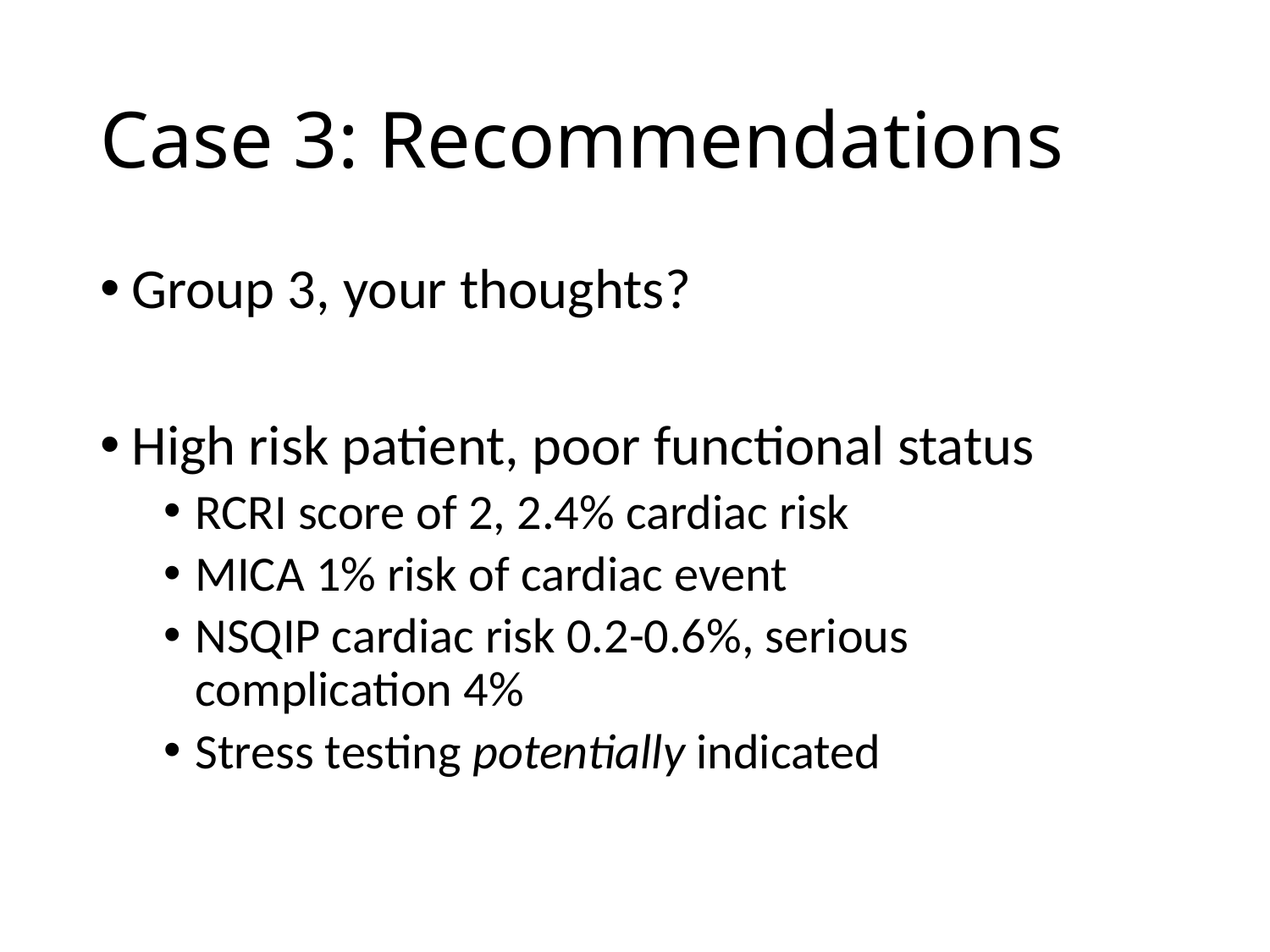

# Case 3: Recommendations
Group 3, your thoughts?
High risk patient, poor functional status
RCRI score of 2, 2.4% cardiac risk
MICA 1% risk of cardiac event
NSQIP cardiac risk 0.2-0.6%, serious complication 4%
Stress testing potentially indicated

## Slide 37
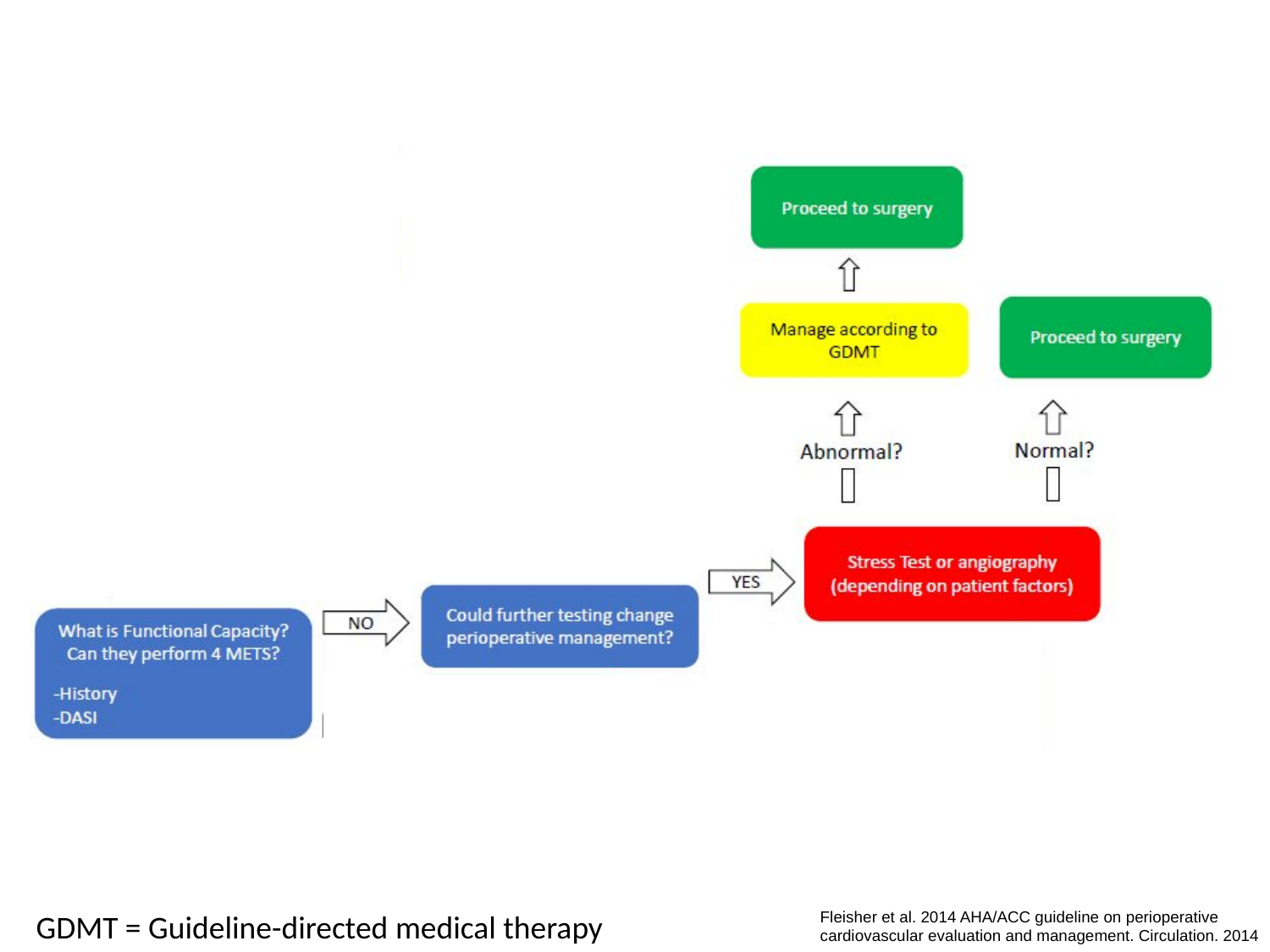

#
GDMT = Guideline-directed medical therapy
Fleisher et al. 2014 AHA/ACC guideline on perioperative cardiovascular evaluation and management. Circulation. 2014

## Slide 38
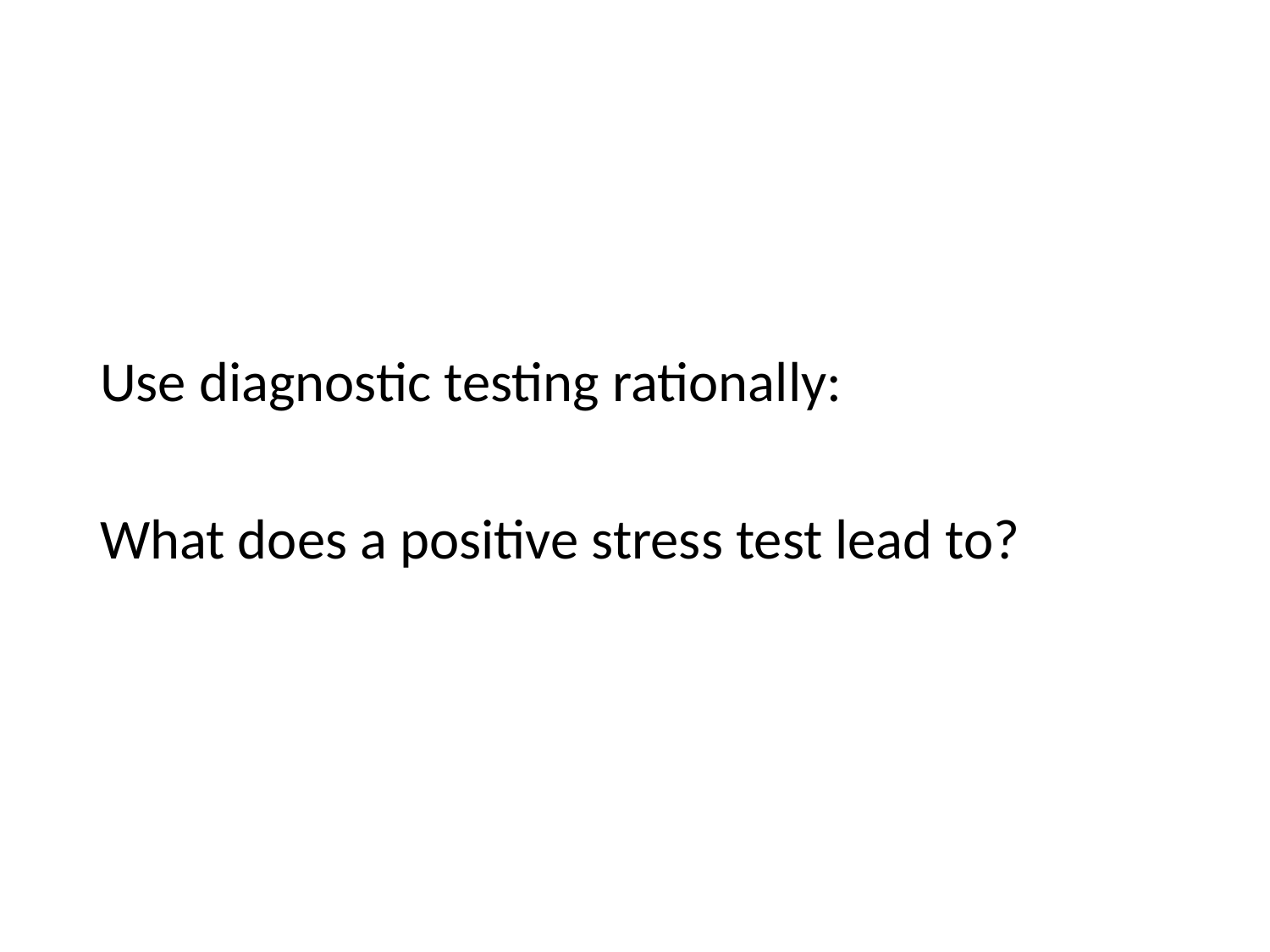

#
Use diagnostic testing rationally:
What does a positive stress test lead to?

## Slide 39
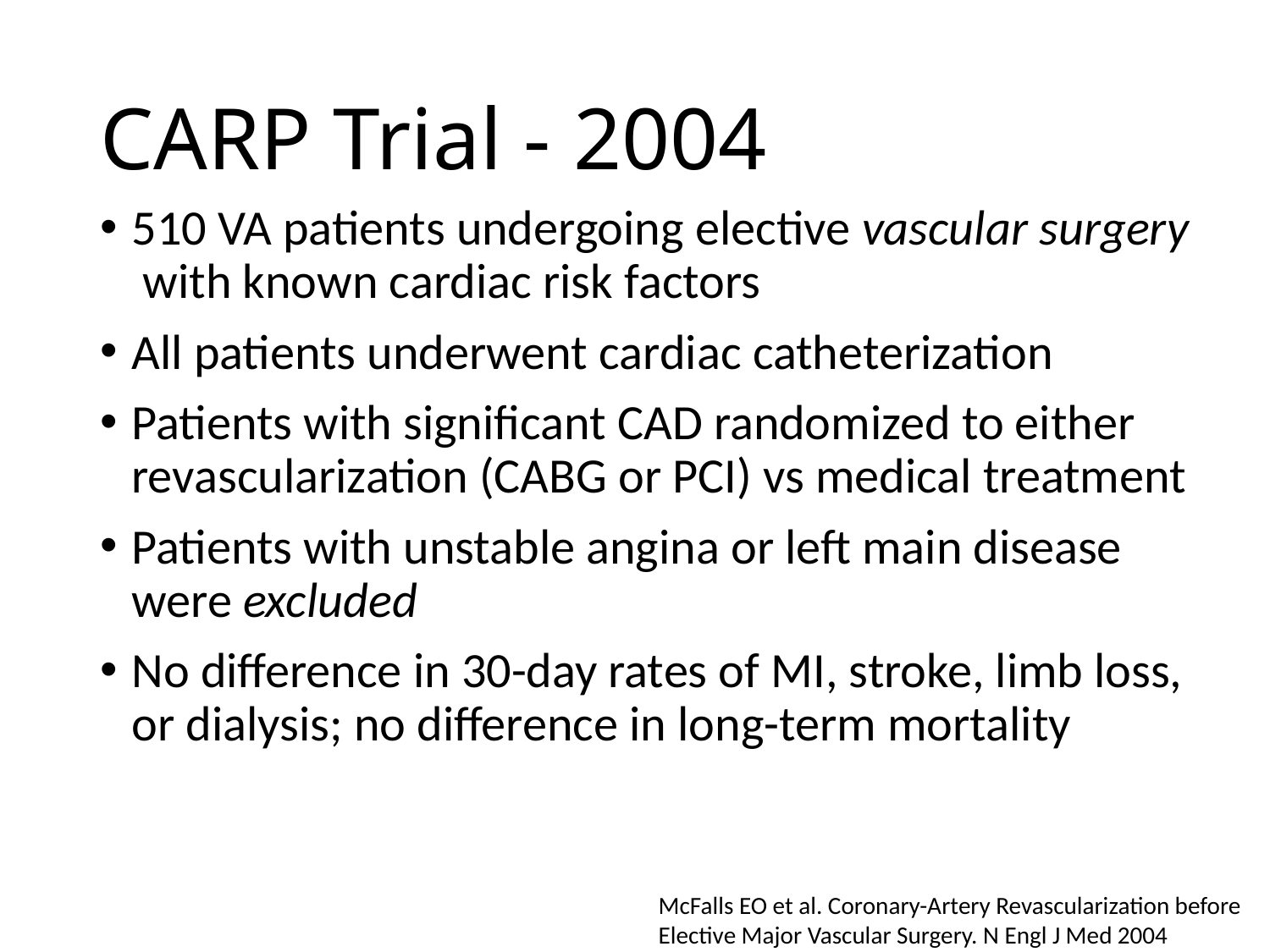

# CARP Trial - 2004
510 VA patients undergoing elective vascular surgery with known cardiac risk factors
All patients underwent cardiac catheterization
Patients with significant CAD randomized to either revascularization (CABG or PCI) vs medical treatment
Patients with unstable angina or left main disease were excluded
No difference in 30-day rates of MI, stroke, limb loss, or dialysis; no difference in long-term mortality
McFalls EO et al. Coronary-Artery Revascularization before Elective Major Vascular Surgery. N Engl J Med 2004

## Slide 40
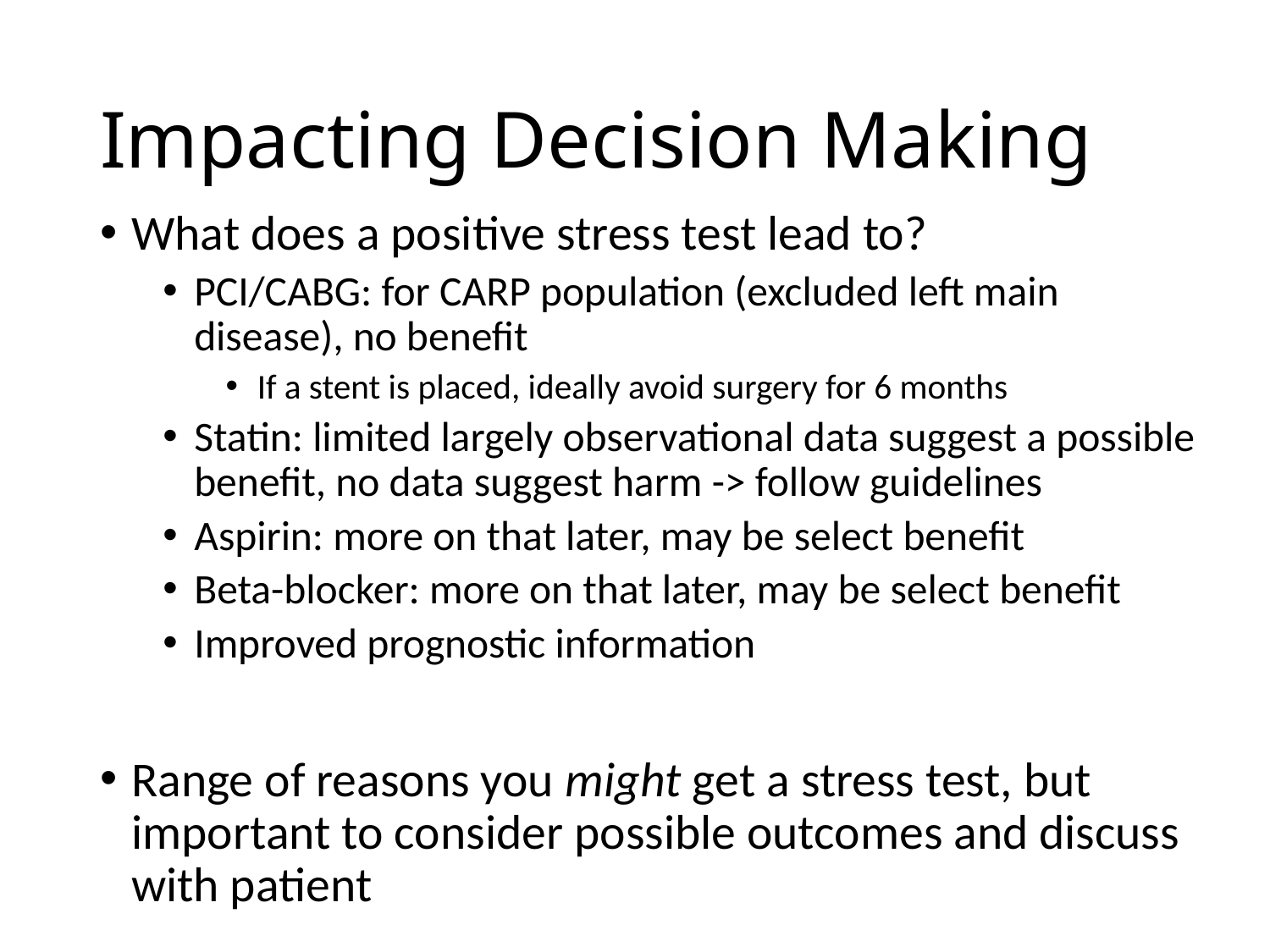

# Impacting Decision Making
What does a positive stress test lead to?
PCI/CABG: for CARP population (excluded left main disease), no benefit
If a stent is placed, ideally avoid surgery for 6 months
Statin: limited largely observational data suggest a possible benefit, no data suggest harm -> follow guidelines
Aspirin: more on that later, may be select benefit
Beta-blocker: more on that later, may be select benefit
Improved prognostic information
Range of reasons you might get a stress test, but important to consider possible outcomes and discuss with patient

## Slide 41
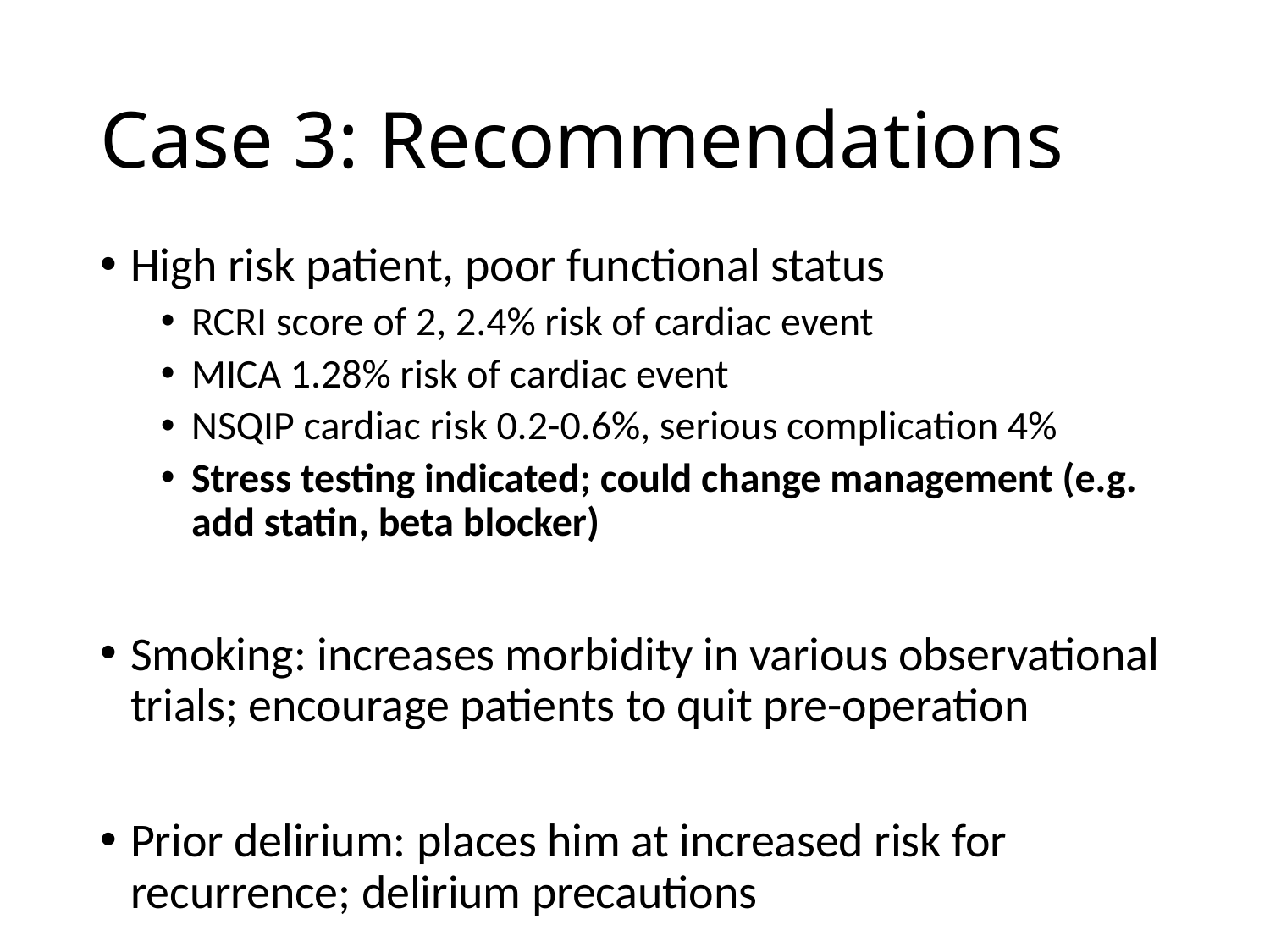

# Case 3: Recommendations
High risk patient, poor functional status
RCRI score of 2, 2.4% risk of cardiac event
MICA 1.28% risk of cardiac event
NSQIP cardiac risk 0.2-0.6%, serious complication 4%
Stress testing indicated; could change management (e.g. add statin, beta blocker)
Smoking: increases morbidity in various observational trials; encourage patients to quit pre-operation
Prior delirium: places him at increased risk for recurrence; delirium precautions

## Slide 42
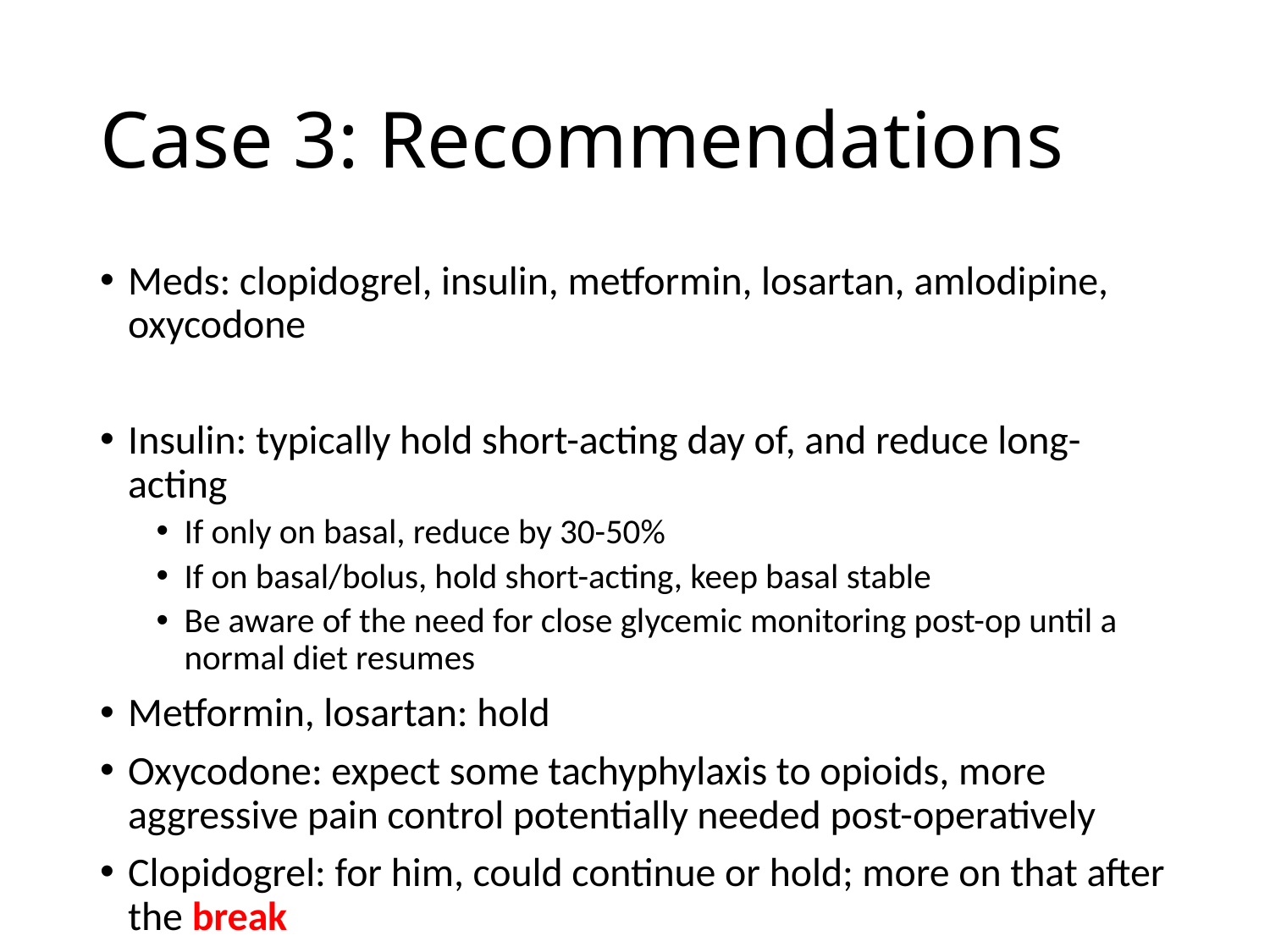

# Case 3: Recommendations
Meds: clopidogrel, insulin, metformin, losartan, amlodipine, oxycodone
Insulin: typically hold short-acting day of, and reduce long-acting
If only on basal, reduce by 30-50%
If on basal/bolus, hold short-acting, keep basal stable
Be aware of the need for close glycemic monitoring post-op until a normal diet resumes
Metformin, losartan: hold
Oxycodone: expect some tachyphylaxis to opioids, more aggressive pain control potentially needed post-operatively
Clopidogrel: for him, could continue or hold; more on that after the break

## Slide 43
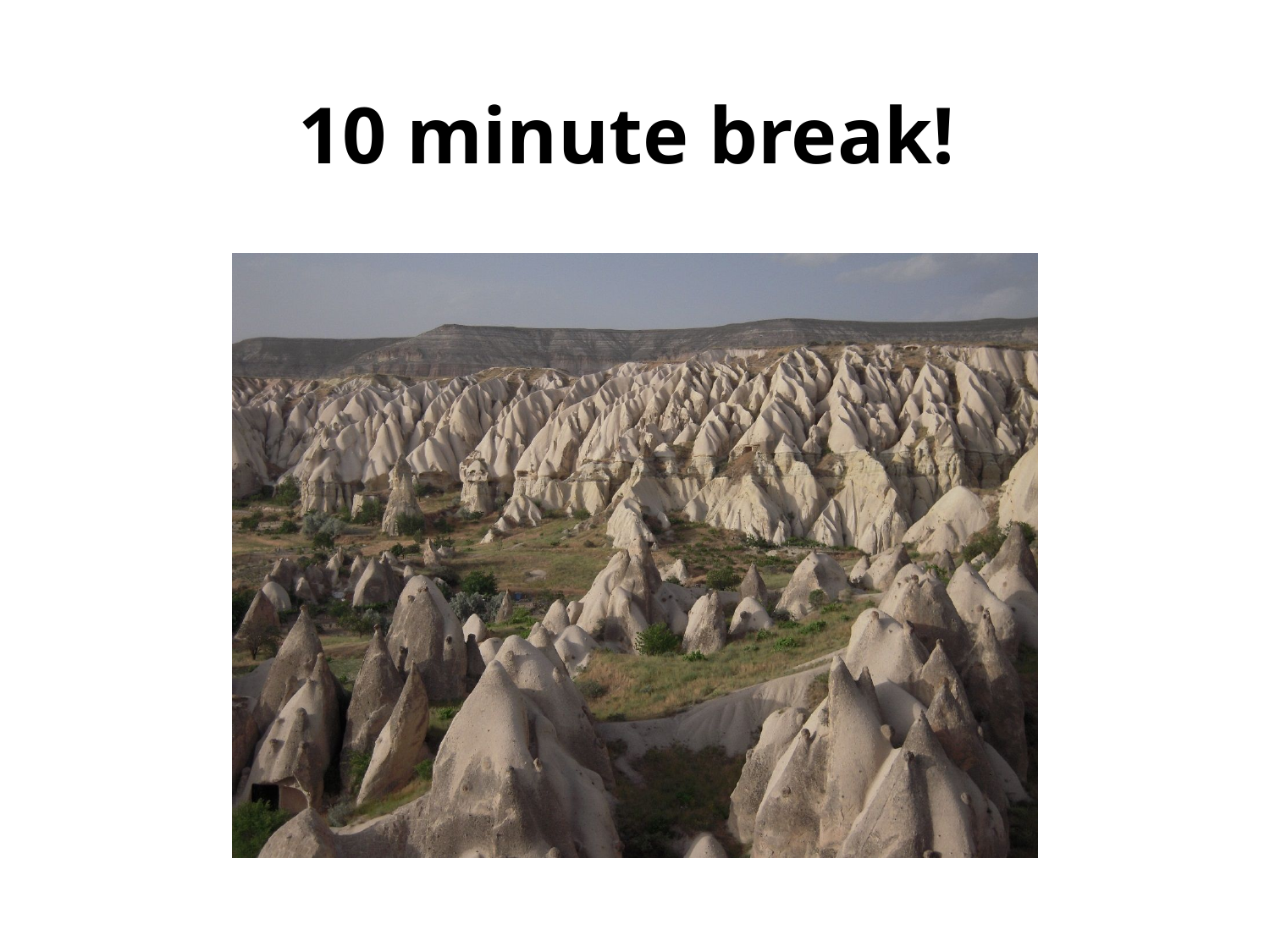

# 10 minute break!

## Slide 44
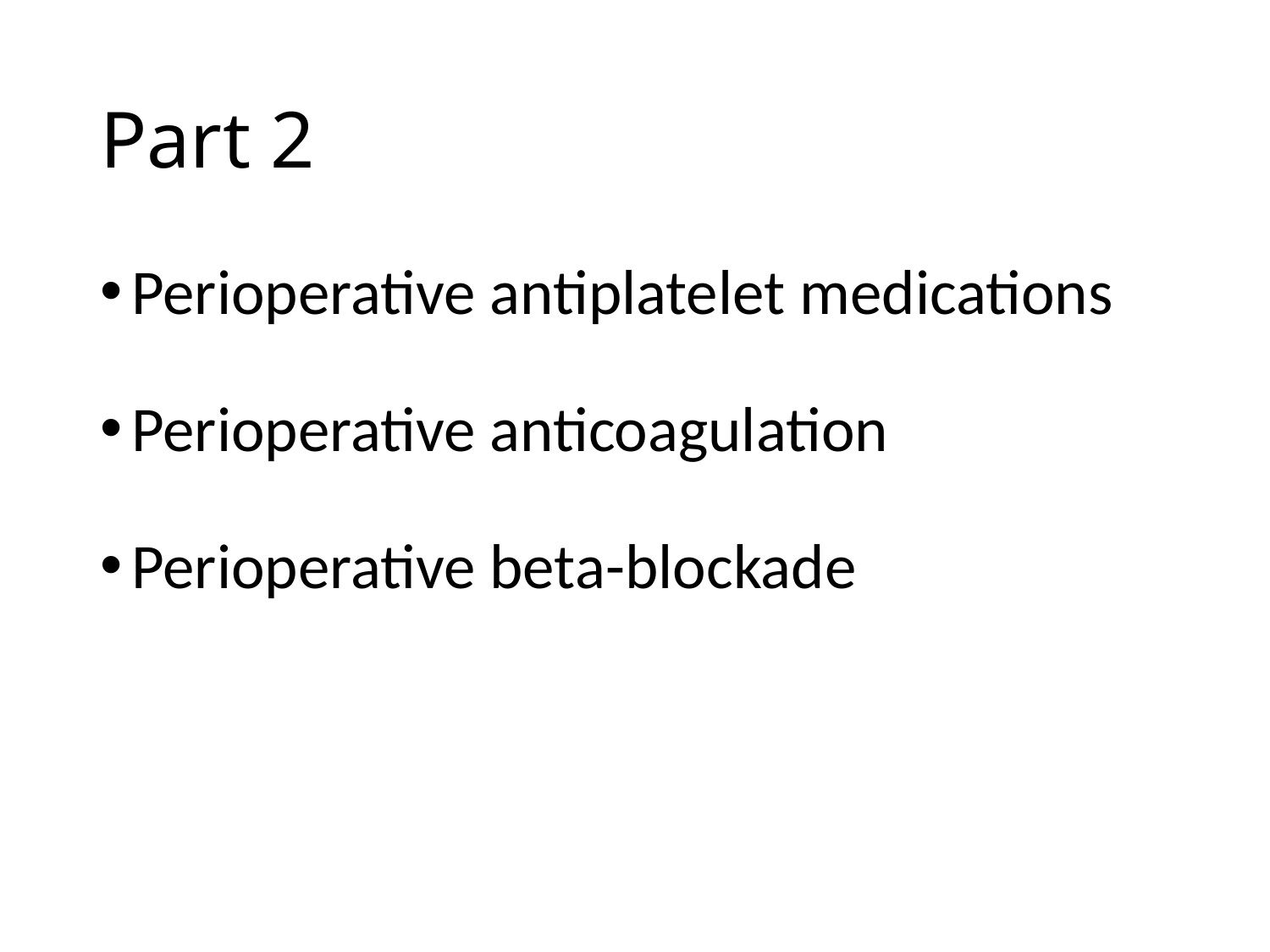

# Part 2
Perioperative antiplatelet medications
Perioperative anticoagulation
Perioperative beta-blockade

## Slide 45
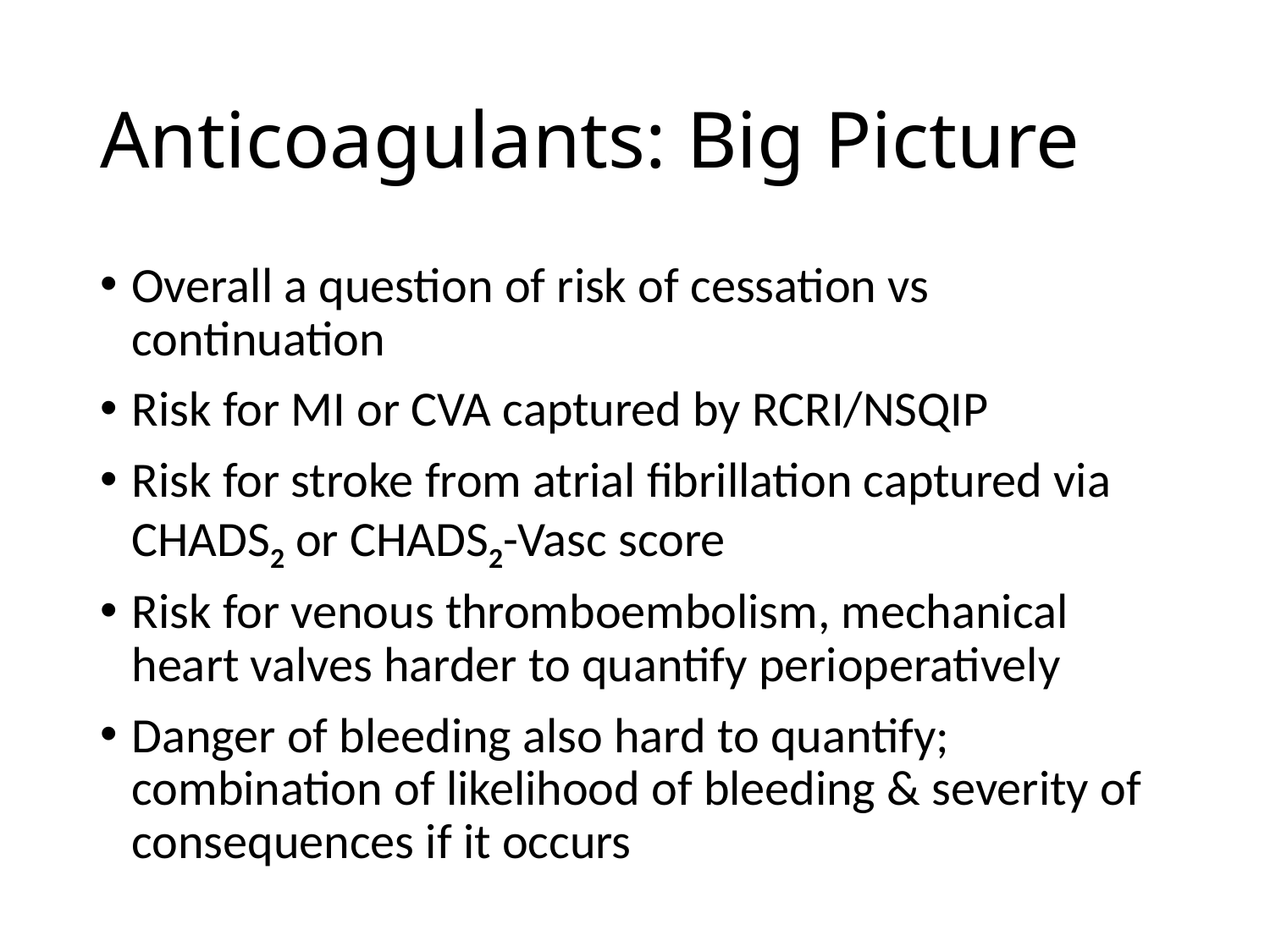

# Anticoagulants: Big Picture
Overall a question of risk of cessation vs continuation
Risk for MI or CVA captured by RCRI/NSQIP
Risk for stroke from atrial fibrillation captured via CHADS2 or CHADS2-Vasc score
Risk for venous thromboembolism, mechanical heart valves harder to quantify perioperatively
Danger of bleeding also hard to quantify; combination of likelihood of bleeding & severity of consequences if it occurs

## Slide 46
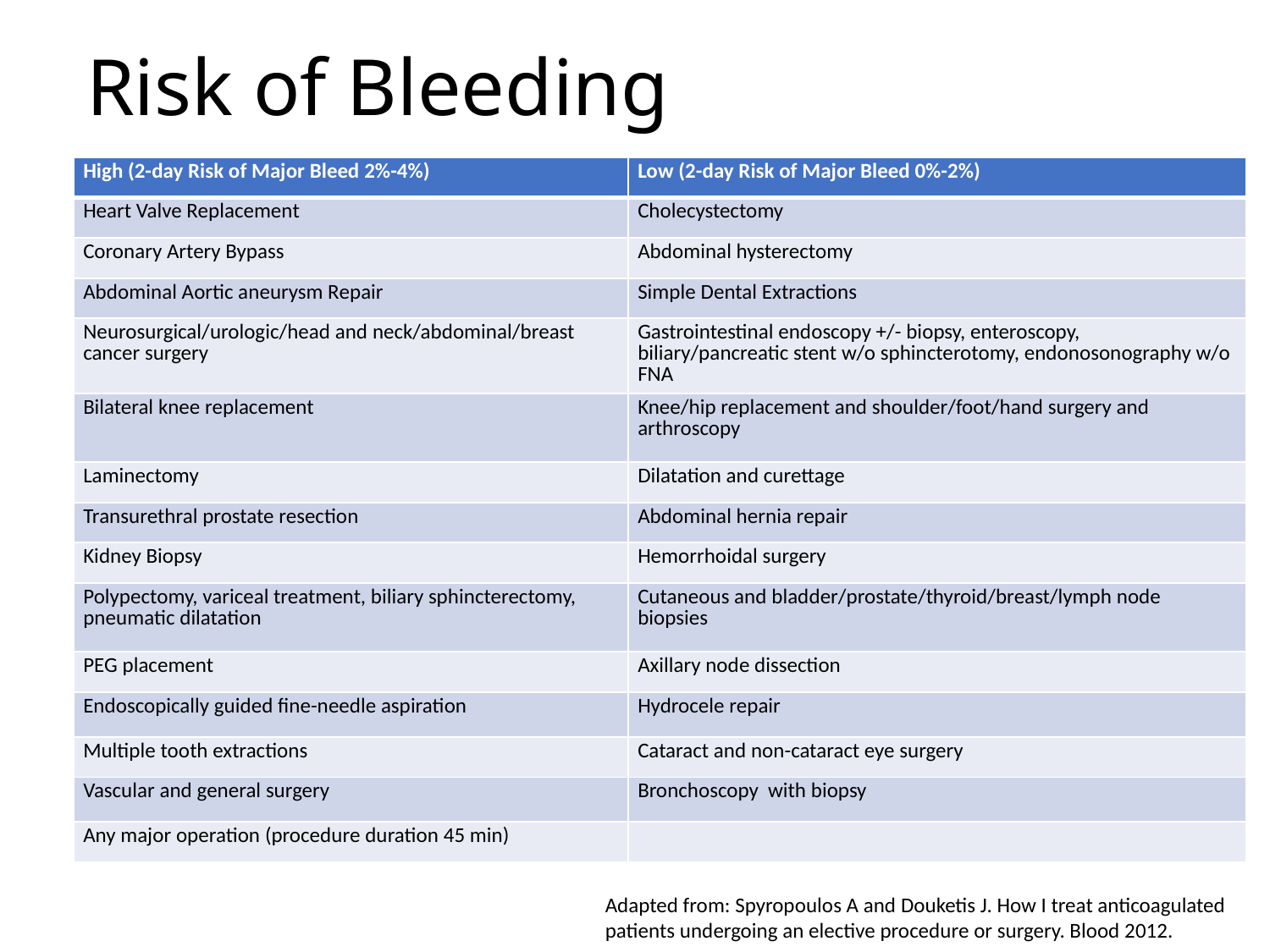

# Risk of Bleeding
| High (2-day Risk of Major Bleed 2%-4%) | Low (2-day Risk of Major Bleed 0%-2%) |
| --- | --- |
| Heart Valve Replacement | Cholecystectomy |
| Coronary Artery Bypass | Abdominal hysterectomy |
| Abdominal Aortic aneurysm Repair | Simple Dental Extractions |
| Neurosurgical/urologic/head and neck/abdominal/breast cancer surgery | Gastrointestinal endoscopy +/- biopsy, enteroscopy, biliary/pancreatic stent w/o sphincterotomy, endonosonography w/o FNA |
| Bilateral knee replacement | Knee/hip replacement and shoulder/foot/hand surgery and arthroscopy |
| Laminectomy | Dilatation and curettage |
| Transurethral prostate resection | Abdominal hernia repair |
| Kidney Biopsy | Hemorrhoidal surgery |
| Polypectomy, variceal treatment, biliary sphincterectomy, pneumatic dilatation | Cutaneous and bladder/prostate/thyroid/breast/lymph node biopsies |
| PEG placement | Axillary node dissection |
| Endoscopically guided fine-needle aspiration | Hydrocele repair |
| Multiple tooth extractions | Cataract and non-cataract eye surgery |
| Vascular and general surgery | Bronchoscopy with biopsy |
| Any major operation (procedure duration 45 min) | |
Adapted from: Spyropoulos A and Douketis J. How I treat anticoagulated patients undergoing an elective procedure or surgery. Blood 2012.

## Slide 47
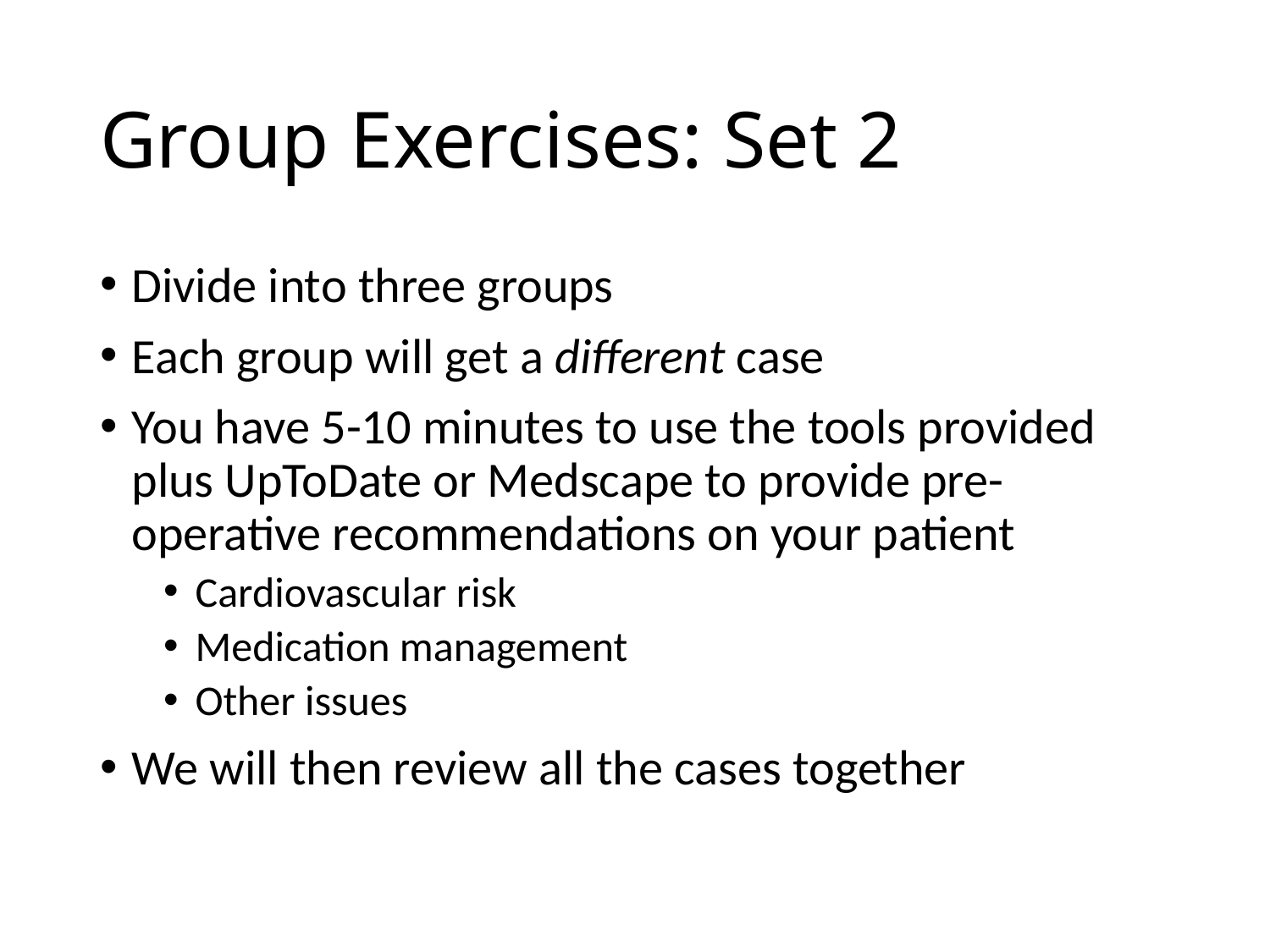

# Group Exercises: Set 2
Divide into three groups
Each group will get a different case
You have 5-10 minutes to use the tools provided plus UpToDate or Medscape to provide pre-operative recommendations on your patient
Cardiovascular risk
Medication management
Other issues
We will then review all the cases together

## Slide 48
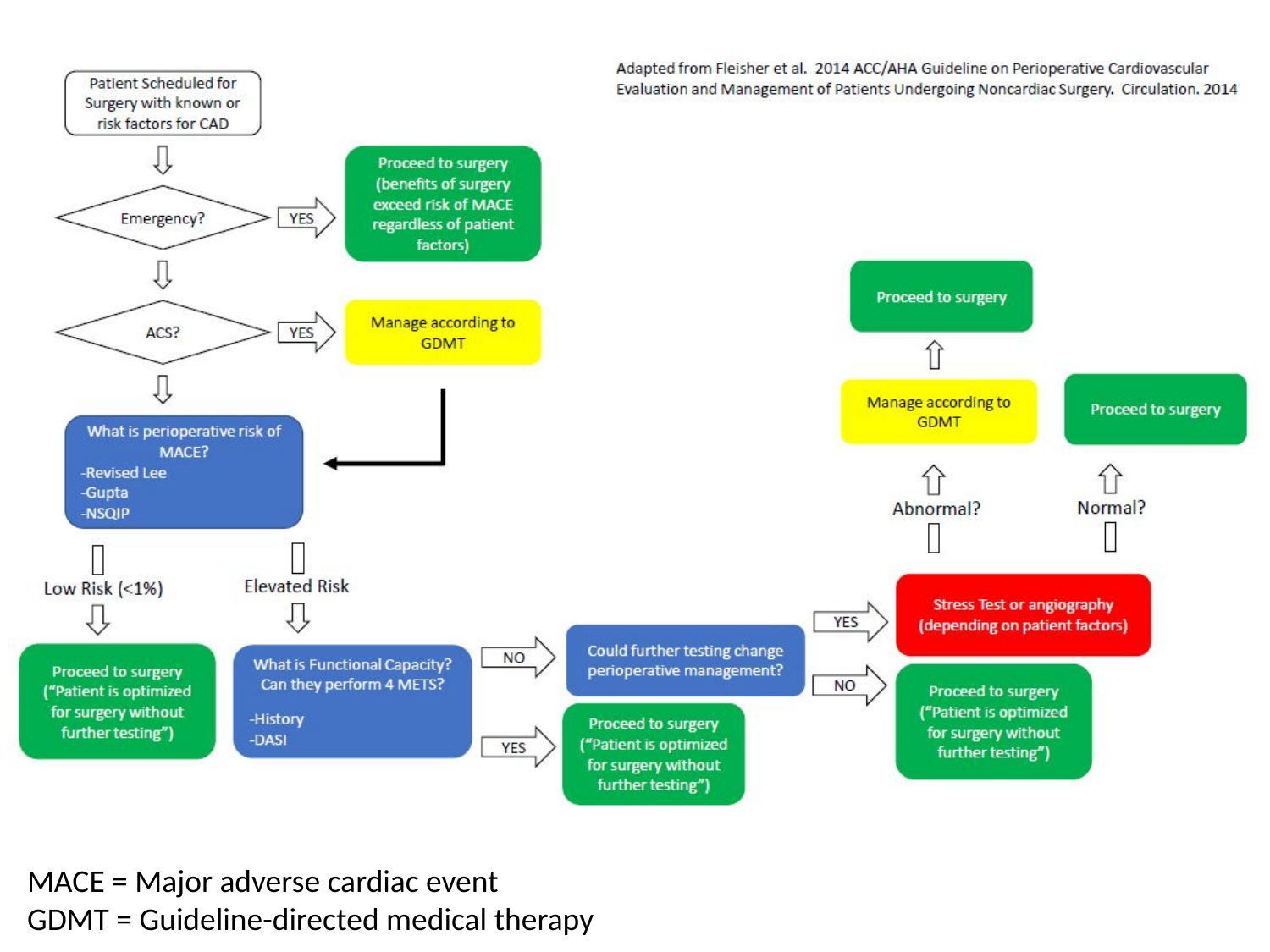

#
MACE = Major adverse cardiac event
GDMT = Guideline-directed medical therapy

## Slide 49
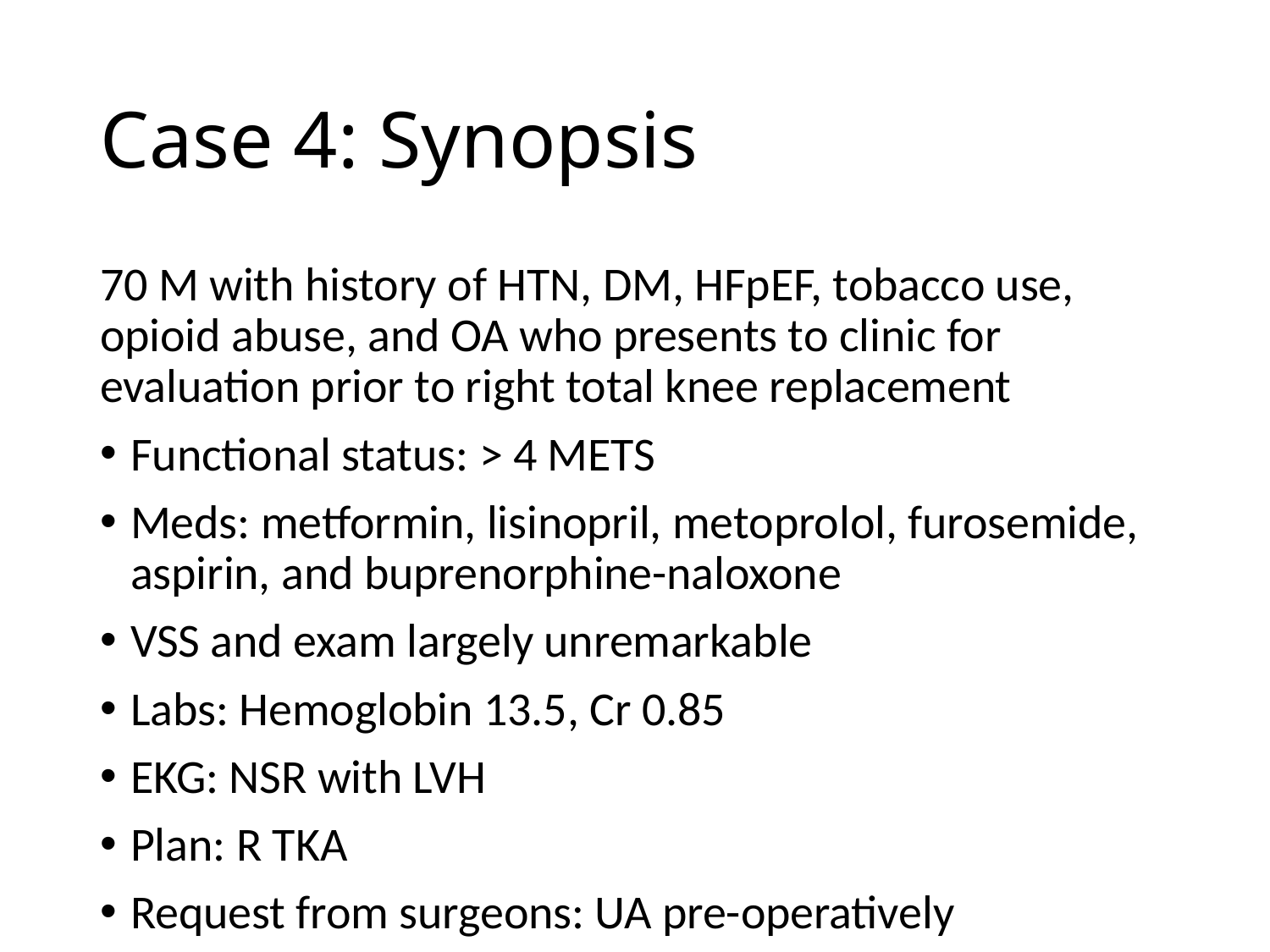

# Case 4: Synopsis
70 M with history of HTN, DM, HFpEF, tobacco use, opioid abuse, and OA who presents to clinic for evaluation prior to right total knee replacement
Functional status: > 4 METS
Meds: metformin, lisinopril, metoprolol, furosemide, aspirin, and buprenorphine-naloxone
VSS and exam largely unremarkable
Labs: Hemoglobin 13.5, Cr 0.85
EKG: NSR with LVH
Plan: R TKA
Request from surgeons: UA pre-operatively

## Slide 50
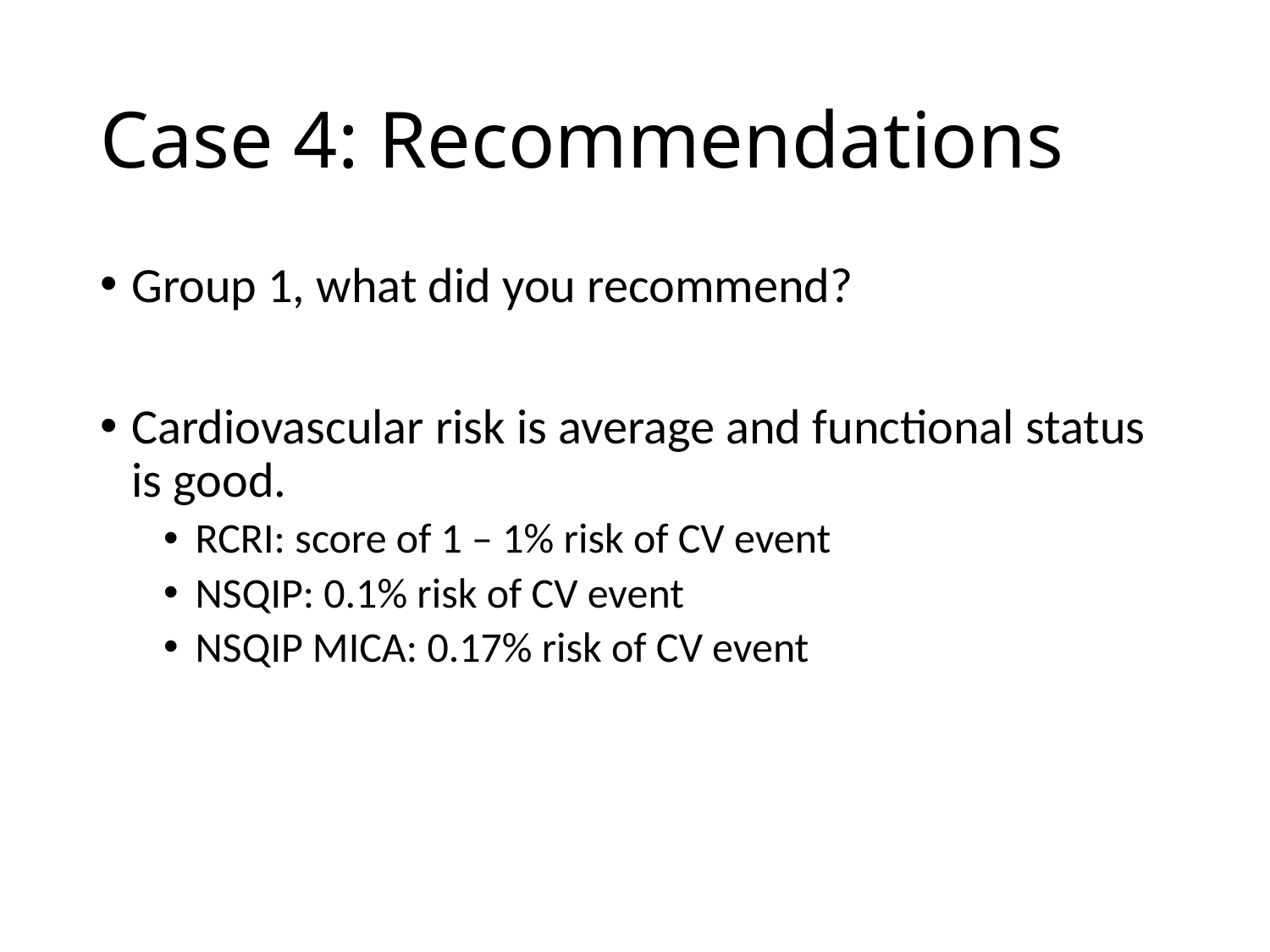

# Case 4: Recommendations
Group 1, what did you recommend?
Cardiovascular risk is average and functional status is good.
RCRI: score of 1 – 1% risk of CV event
NSQIP: 0.1% risk of CV event
NSQIP MICA: 0.17% risk of CV event

## Slide 51
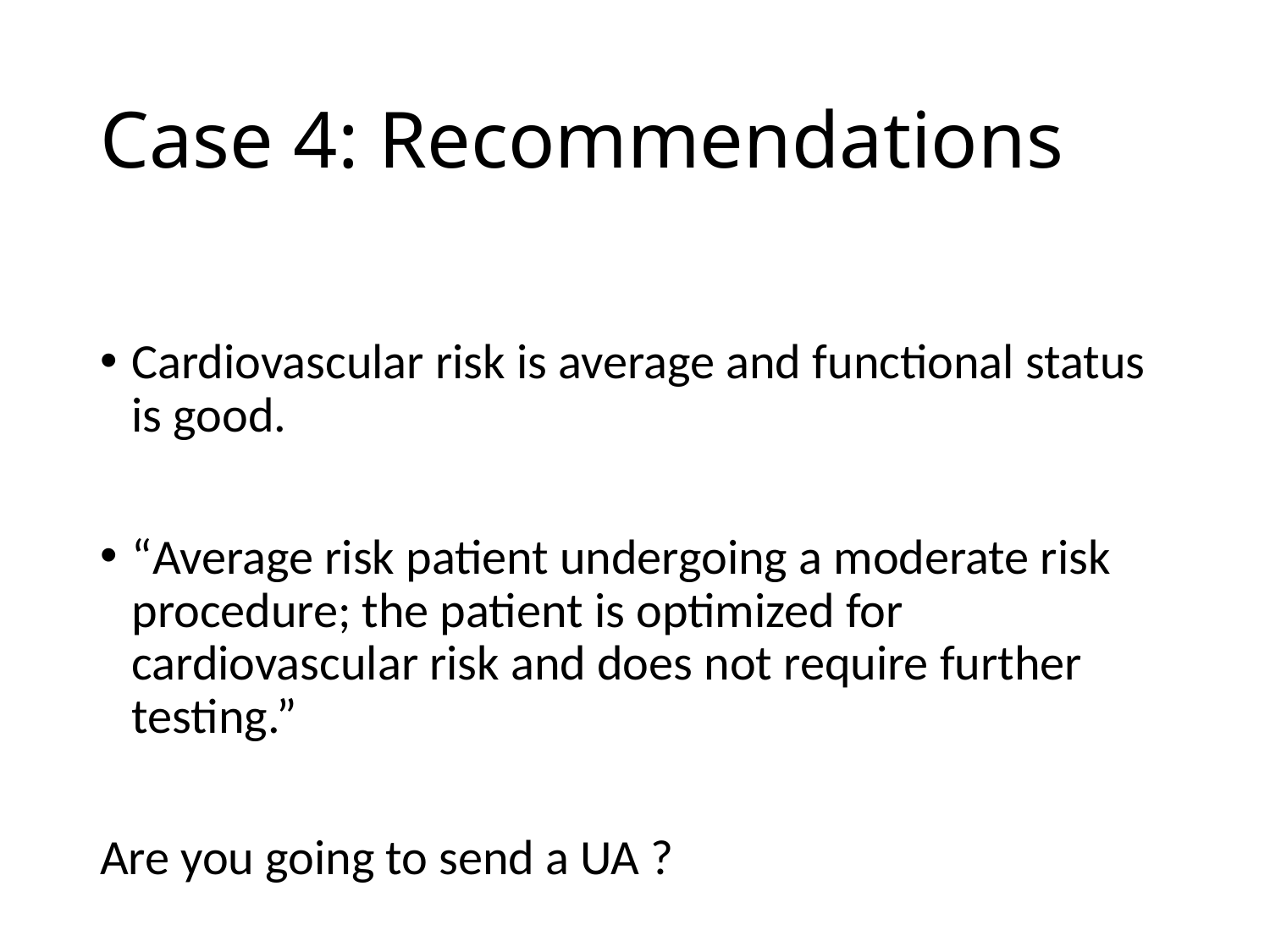

# Case 4: Recommendations
Cardiovascular risk is average and functional status is good.
“Average risk patient undergoing a moderate risk procedure; the patient is optimized for cardiovascular risk and does not require further testing.”
Are you going to send a UA ?

## Slide 52
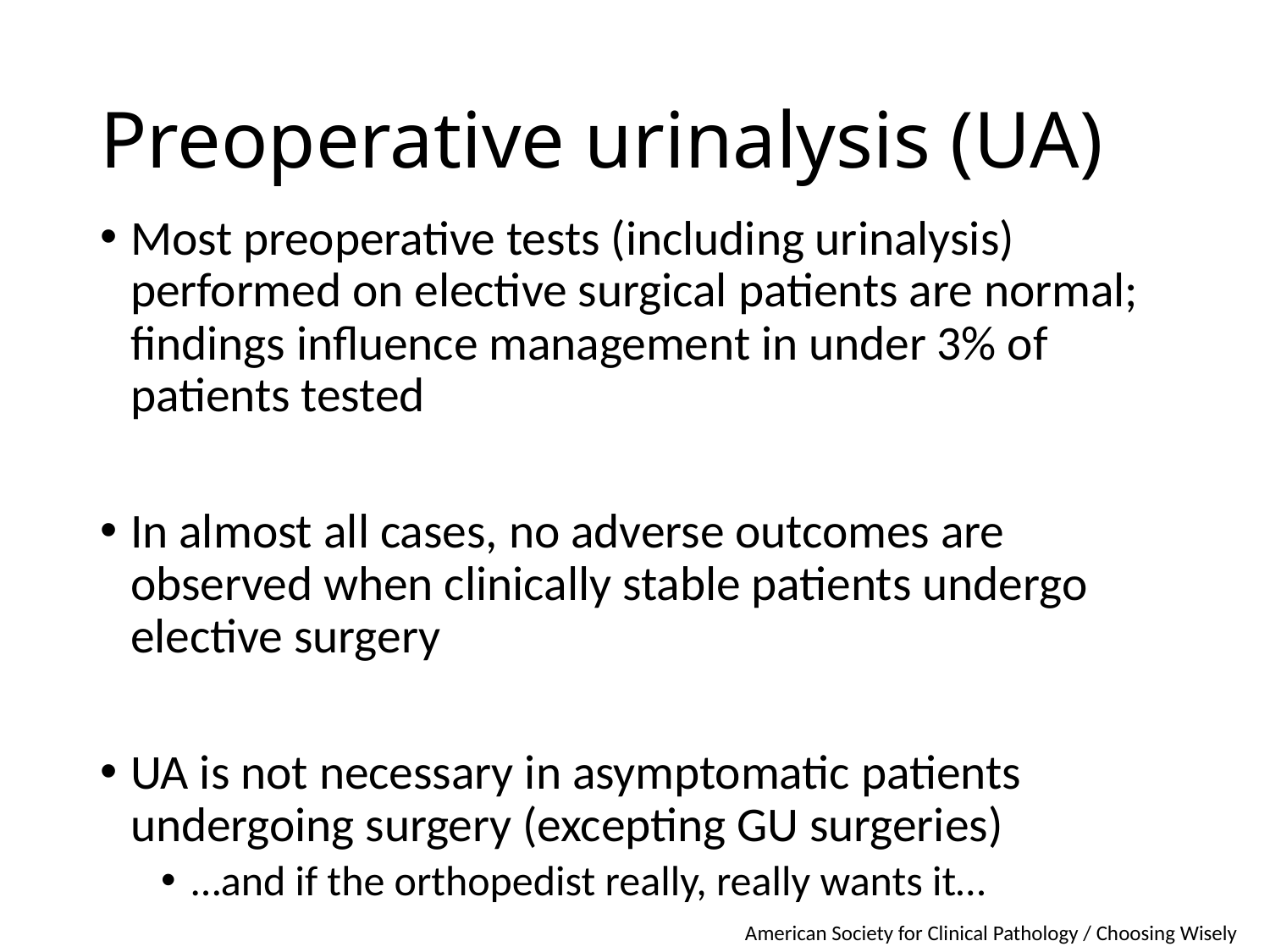

# Preoperative urinalysis (UA)
Most preoperative tests (including urinalysis) performed on elective surgical patients are normal; findings influence management in under 3% of patients tested
In almost all cases, no adverse outcomes are observed when clinically stable patients undergo elective surgery
UA is not necessary in asymptomatic patients undergoing surgery (excepting GU surgeries)
…and if the orthopedist really, really wants it…
American Society for Clinical Pathology / Choosing Wisely

## Slide 53
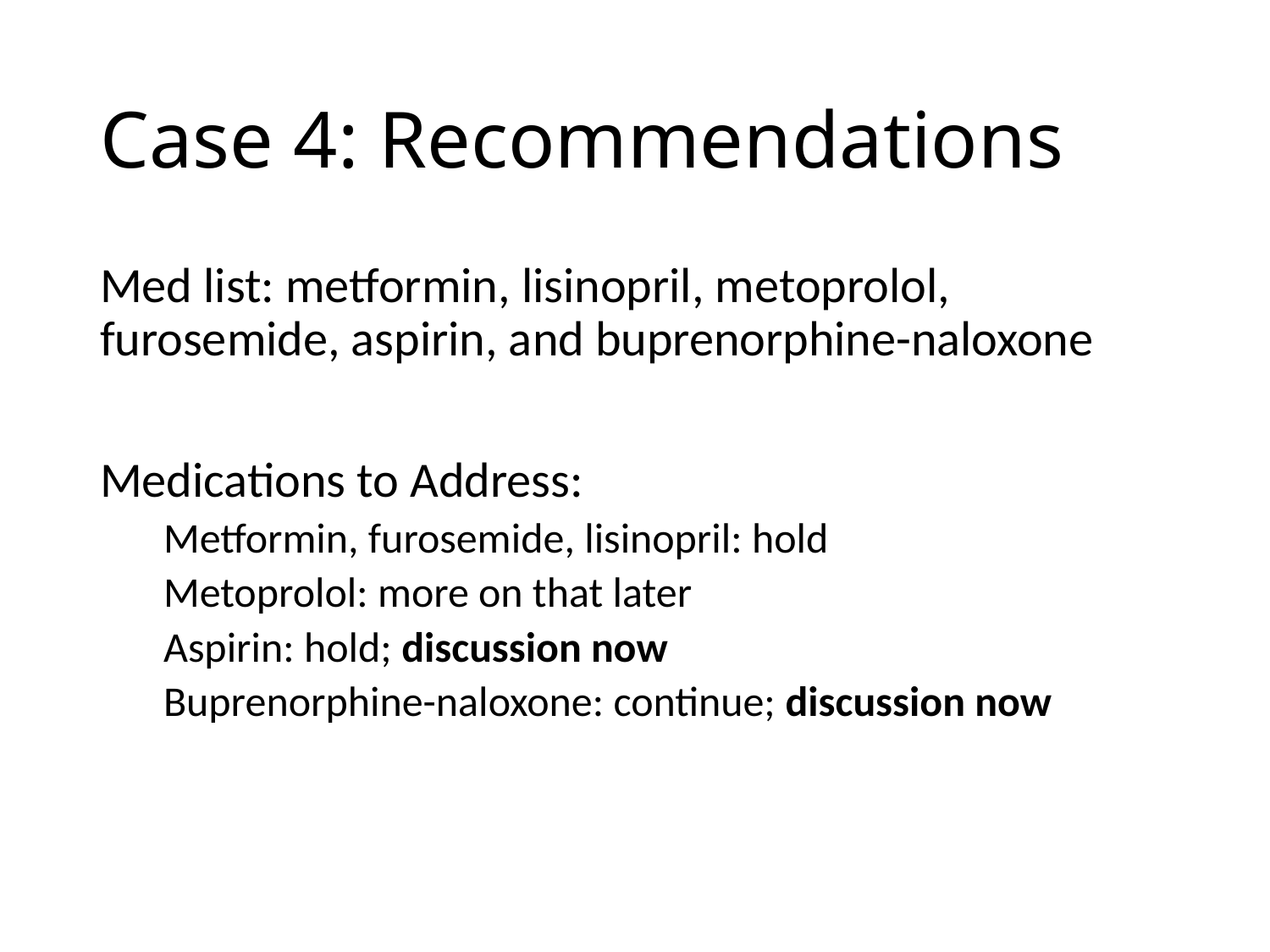

# Case 4: Recommendations
Med list: metformin, lisinopril, metoprolol, furosemide, aspirin, and buprenorphine-naloxone
Medications to Address:
Metformin, furosemide, lisinopril: hold
Metoprolol: more on that later
Aspirin: hold; discussion now
Buprenorphine-naloxone: continue; discussion now

## Slide 54
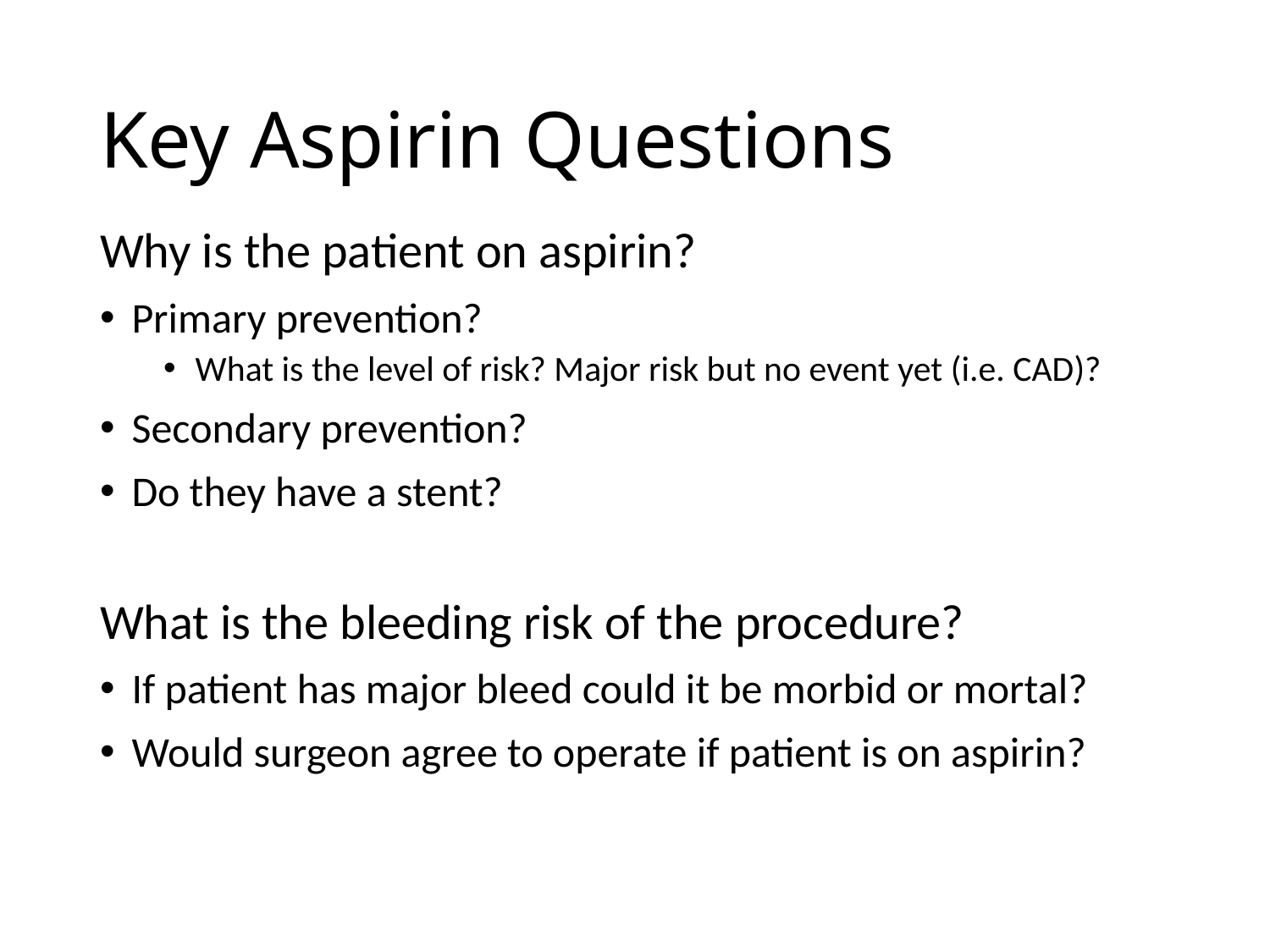

# Key Aspirin Questions
Why is the patient on aspirin?
Primary prevention?
What is the level of risk? Major risk but no event yet (i.e. CAD)?
Secondary prevention?
Do they have a stent?
What is the bleeding risk of the procedure?
If patient has major bleed could it be morbid or mortal?
Would surgeon agree to operate if patient is on aspirin?

## Slide 55
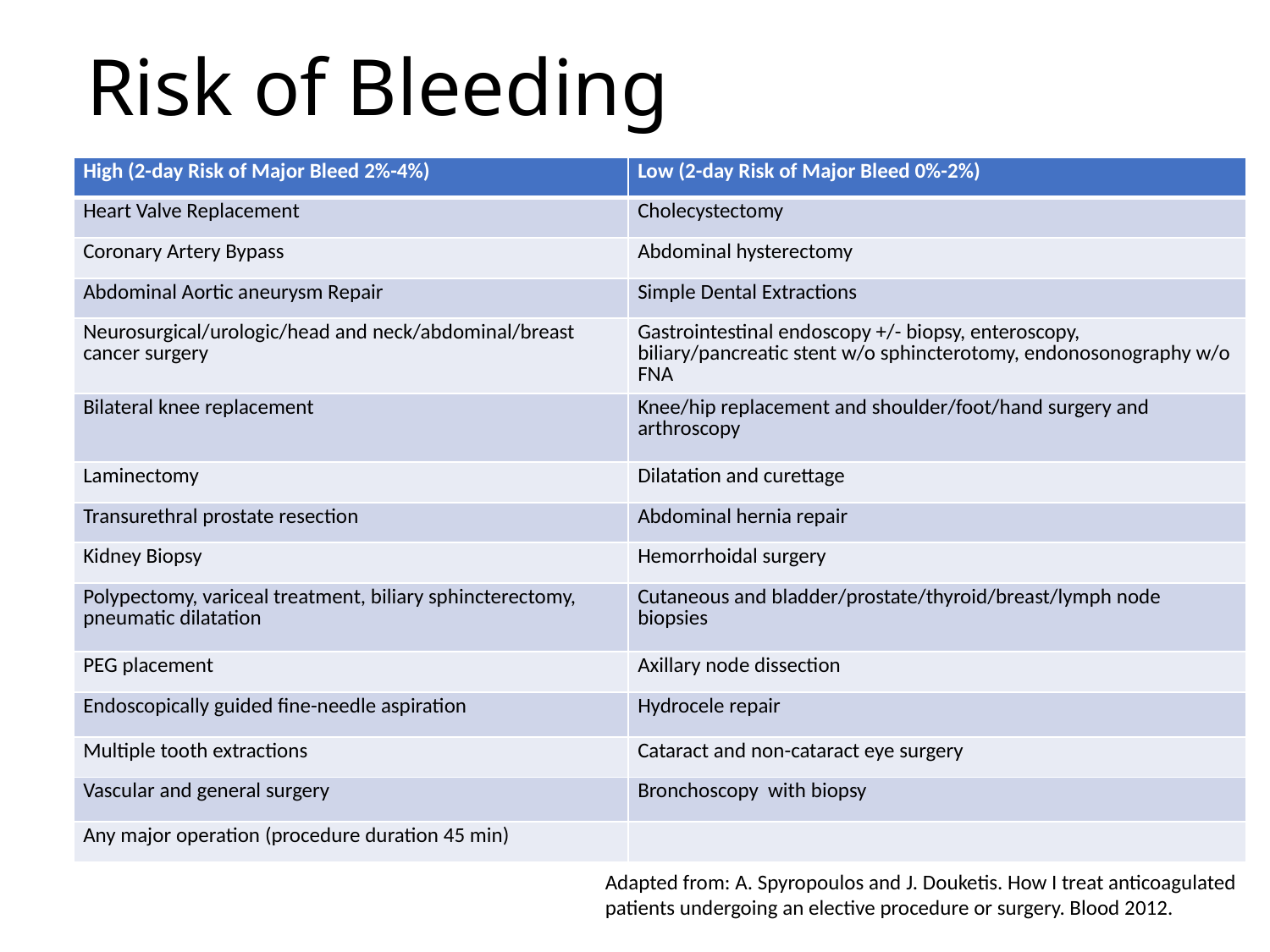

# Risk of Bleeding
| High (2-day Risk of Major Bleed 2%-4%) | Low (2-day Risk of Major Bleed 0%-2%) |
| --- | --- |
| Heart Valve Replacement | Cholecystectomy |
| Coronary Artery Bypass | Abdominal hysterectomy |
| Abdominal Aortic aneurysm Repair | Simple Dental Extractions |
| Neurosurgical/urologic/head and neck/abdominal/breast cancer surgery | Gastrointestinal endoscopy +/- biopsy, enteroscopy, biliary/pancreatic stent w/o sphincterotomy, endonosonography w/o FNA |
| Bilateral knee replacement | Knee/hip replacement and shoulder/foot/hand surgery and arthroscopy |
| Laminectomy | Dilatation and curettage |
| Transurethral prostate resection | Abdominal hernia repair |
| Kidney Biopsy | Hemorrhoidal surgery |
| Polypectomy, variceal treatment, biliary sphincterectomy, pneumatic dilatation | Cutaneous and bladder/prostate/thyroid/breast/lymph node biopsies |
| PEG placement | Axillary node dissection |
| Endoscopically guided fine-needle aspiration | Hydrocele repair |
| Multiple tooth extractions | Cataract and non-cataract eye surgery |
| Vascular and general surgery | Bronchoscopy with biopsy |
| Any major operation (procedure duration 45 min) | |
Adapted from: A. Spyropoulos and J. Douketis. How I treat anticoagulated patients undergoing an elective procedure or surgery. Blood 2012.

## Slide 56
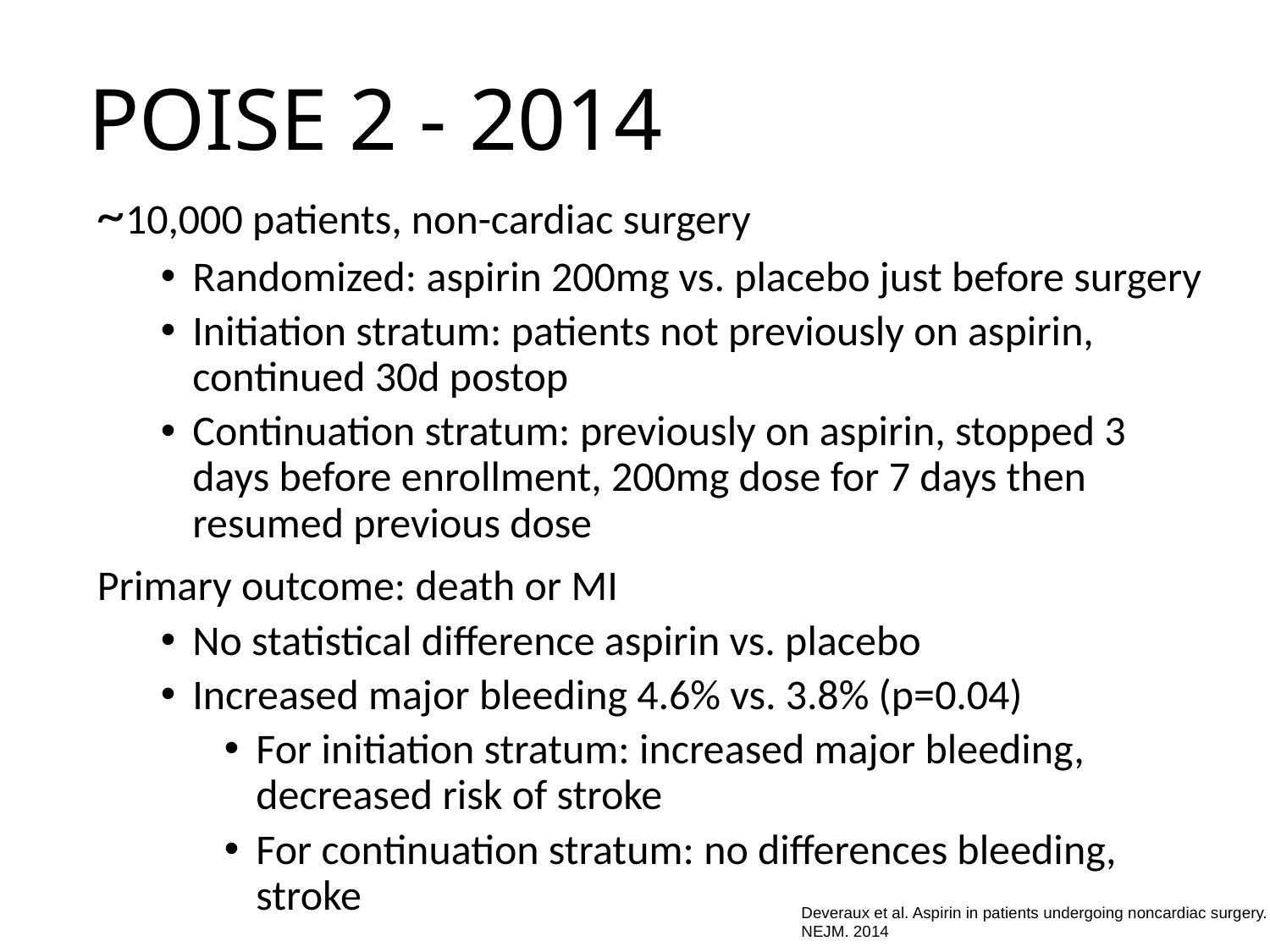

# POISE 2 - 2014
~10,000 patients, non-cardiac surgery
Randomized: aspirin 200mg vs. placebo just before surgery
Initiation stratum: patients not previously on aspirin, continued 30d postop
Continuation stratum: previously on aspirin, stopped 3 days before enrollment, 200mg dose for 7 days then resumed previous dose
Primary outcome: death or MI
No statistical difference aspirin vs. placebo
Increased major bleeding 4.6% vs. 3.8% (p=0.04)
For initiation stratum: increased major bleeding, decreased risk of stroke
For continuation stratum: no differences bleeding, stroke
Deveraux et al. Aspirin in patients undergoing noncardiac surgery. NEJM. 2014

## Slide 57
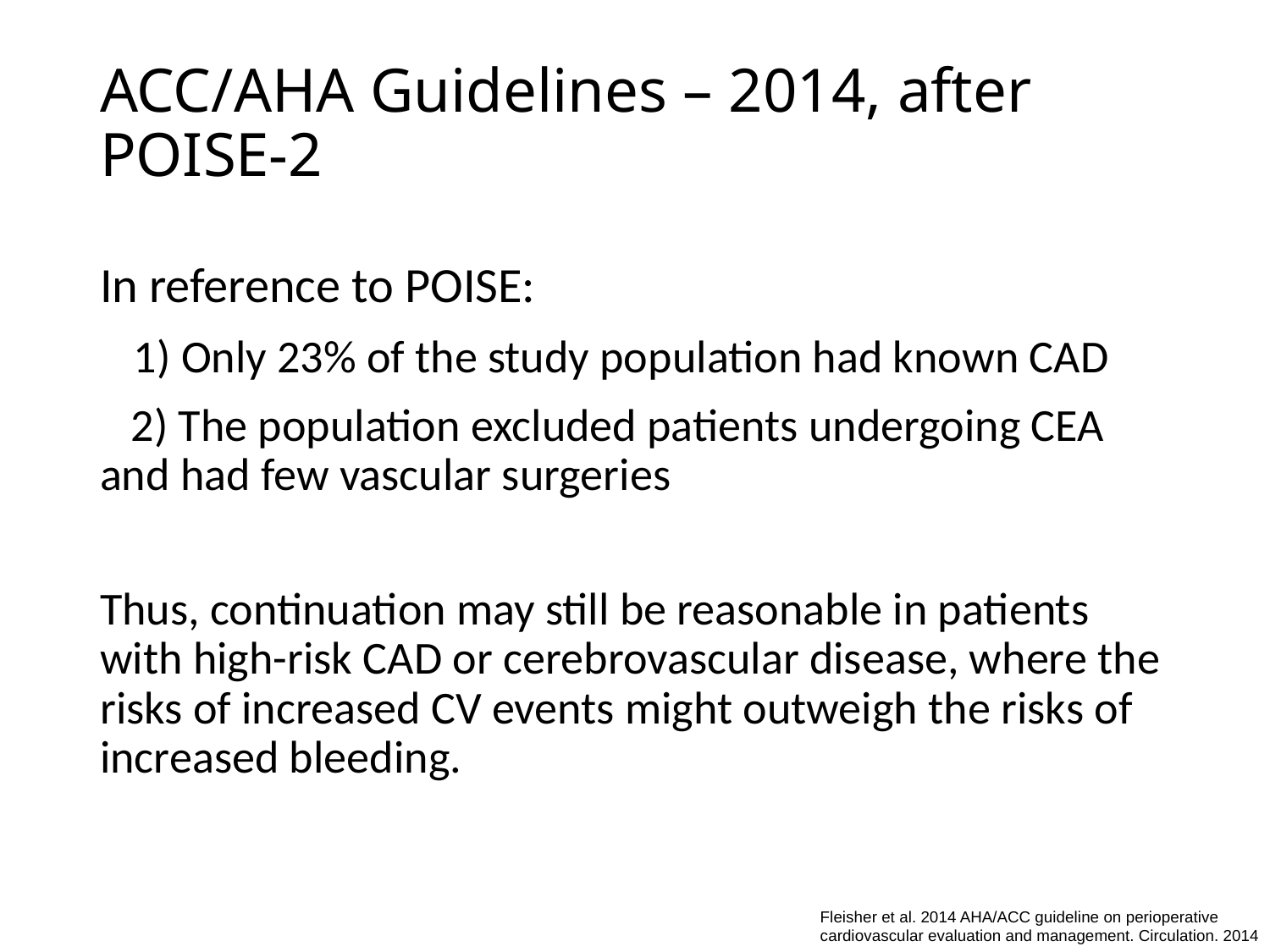

ACC/AHA Guidelines – 2014, after POISE-2
In reference to POISE:
  1) Only 23% of the study population had known CAD
 2) The population excluded patients undergoing CEA and had few vascular surgeries
Thus, continuation may still be reasonable in patients with high-risk CAD or cerebrovascular disease, where the risks of increased CV events might outweigh the risks of increased bleeding.
Fleisher et al. 2014 AHA/ACC guideline on perioperative cardiovascular evaluation and management. Circulation. 2014

## Slide 58
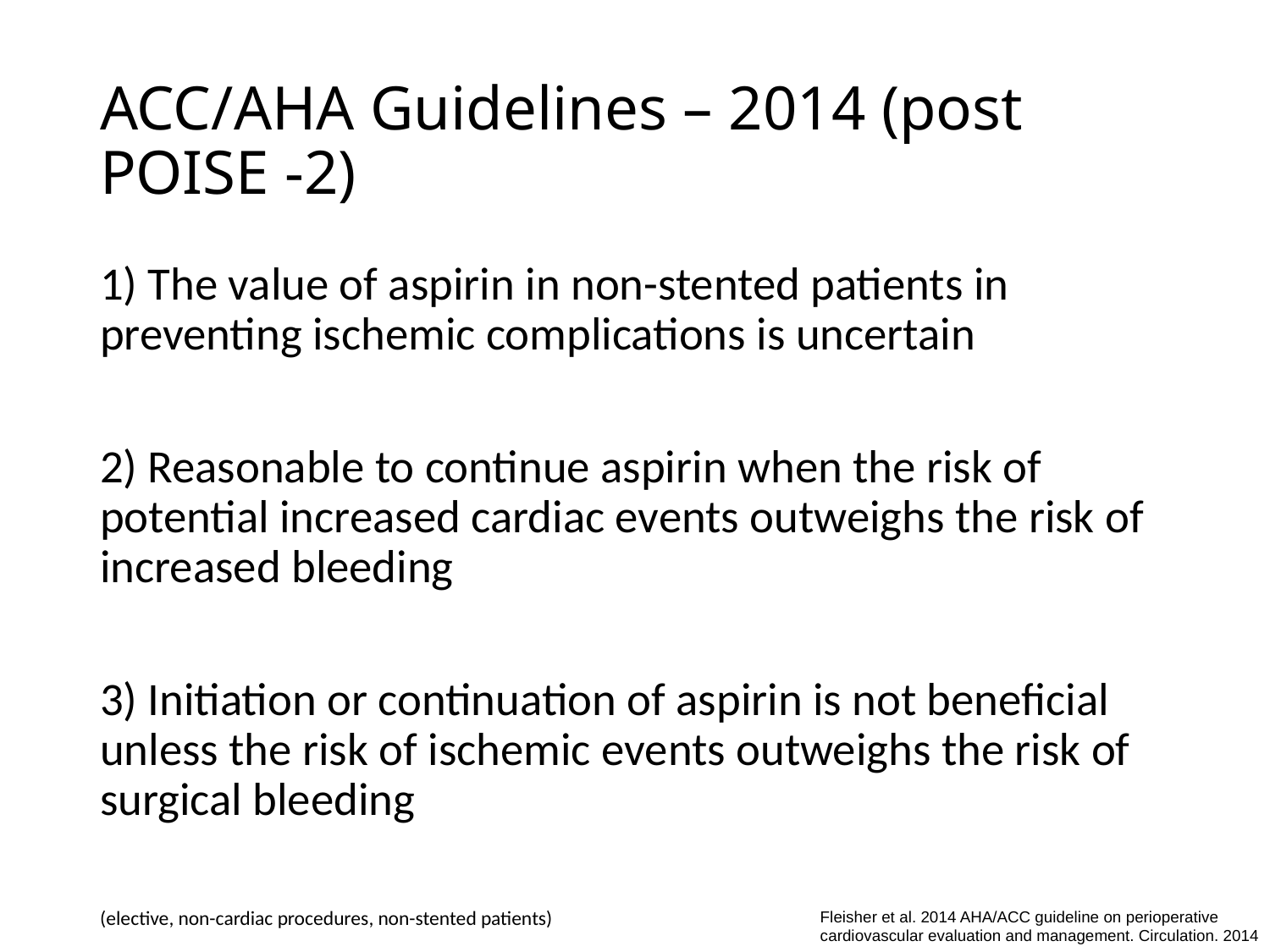

# ACC/AHA Guidelines – 2014 (post POISE -2)
1) The value of aspirin in non-stented patients in preventing ischemic complications is uncertain
2) Reasonable to continue aspirin when the risk of potential increased cardiac events outweighs the risk of increased bleeding
3) Initiation or continuation of aspirin is not beneficial unless the risk of ischemic events outweighs the risk of surgical bleeding
(elective, non-cardiac procedures, non-stented patients)
Fleisher et al. 2014 AHA/ACC guideline on perioperative cardiovascular evaluation and management. Circulation. 2014

## Slide 59
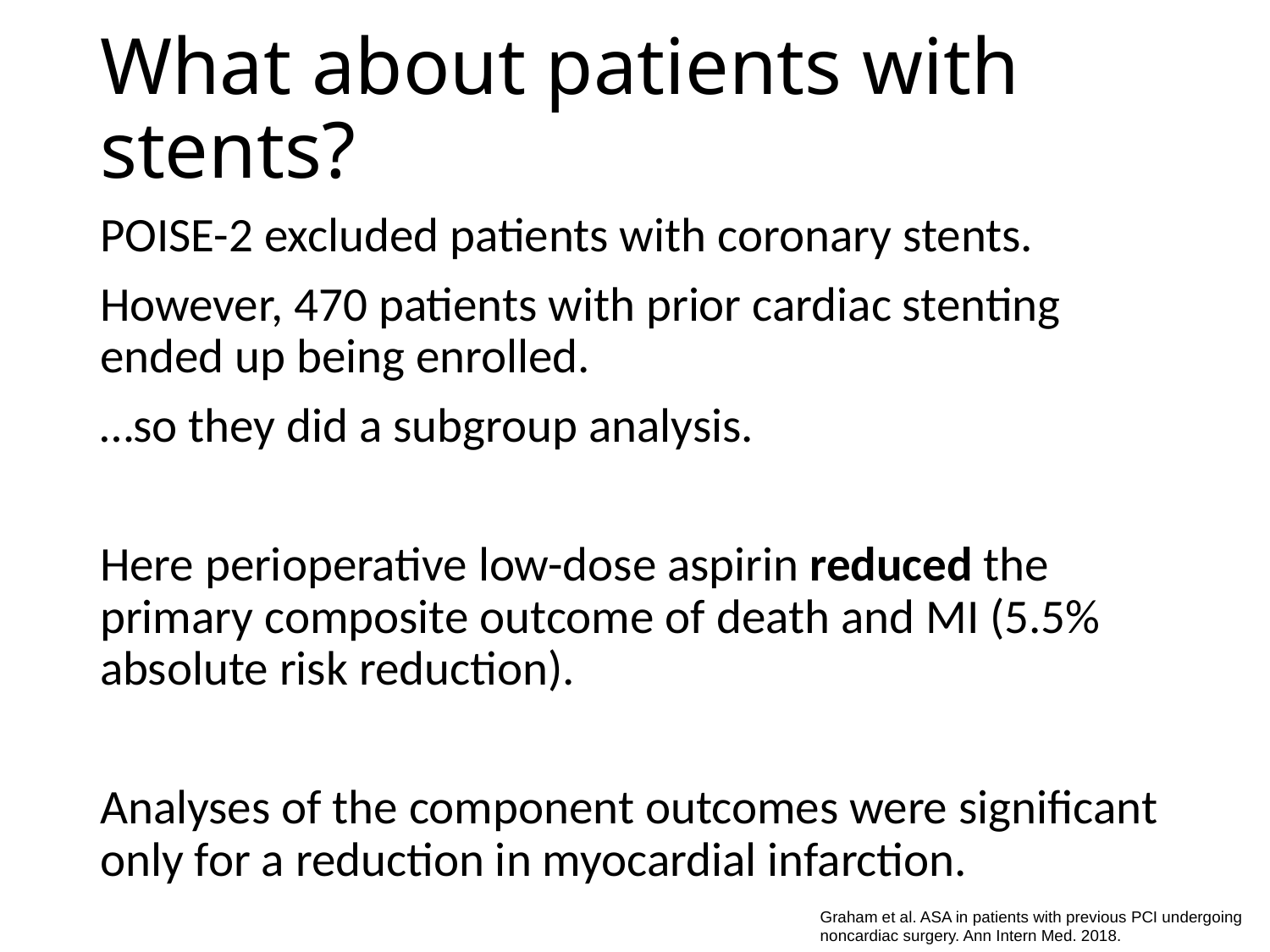

# What about patients with stents?
POISE-2 excluded patients with coronary stents.
However, 470 patients with prior cardiac stenting ended up being enrolled.
…so they did a subgroup analysis.
Here perioperative low-dose aspirin reduced the primary composite outcome of death and MI (5.5% absolute risk reduction).
Analyses of the component outcomes were significant only for a reduction in myocardial infarction.
Graham et al. ASA in patients with previous PCI undergoing noncardiac surgery. Ann Intern Med. 2018.

## Slide 60
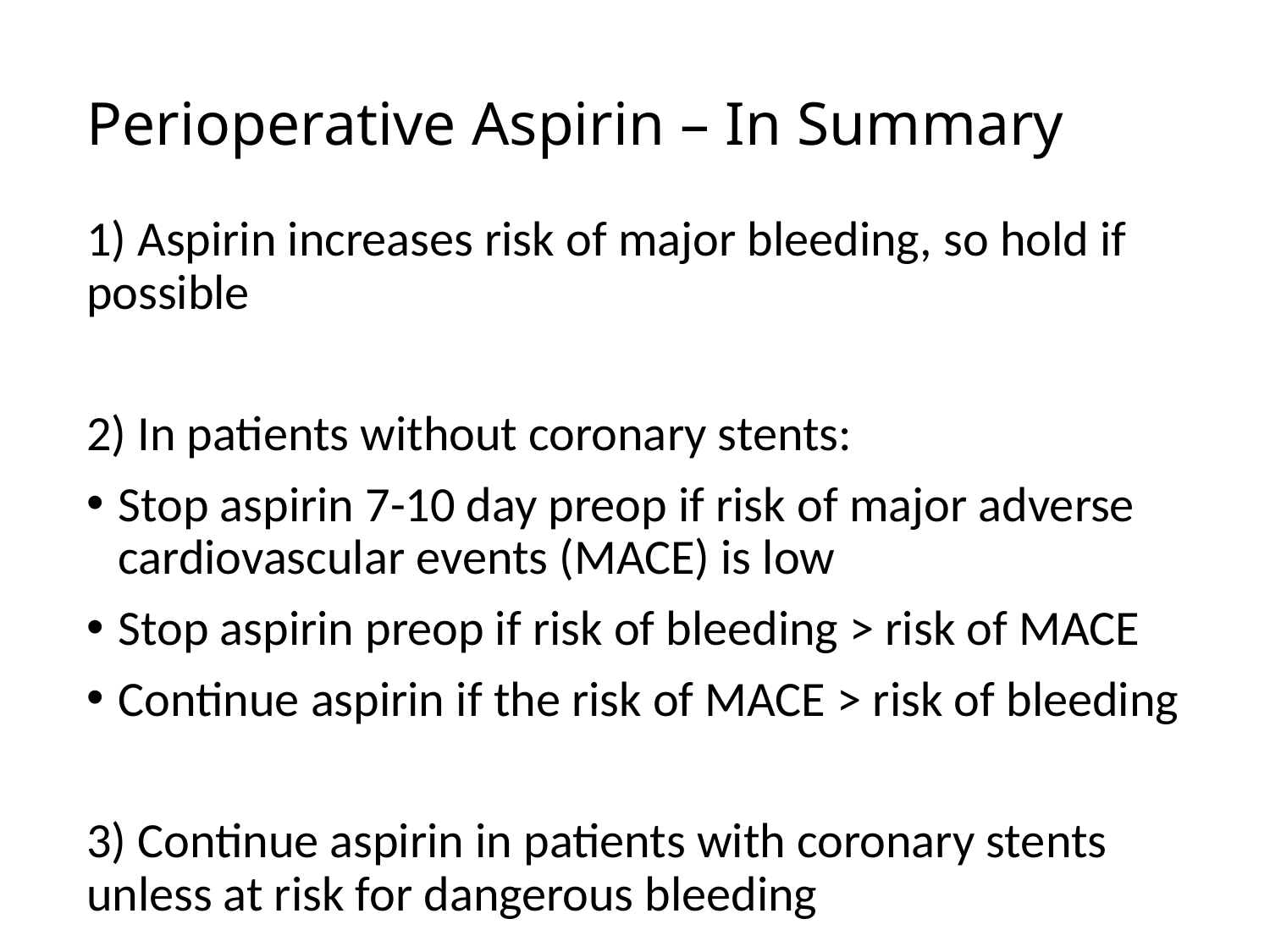

# Perioperative Aspirin – In Summary
1) Aspirin increases risk of major bleeding, so hold if possible
2) In patients without coronary stents:
Stop aspirin 7-10 day preop if risk of major adverse cardiovascular events (MACE) is low
Stop aspirin preop if risk of bleeding > risk of MACE
Continue aspirin if the risk of MACE > risk of bleeding
3) Continue aspirin in patients with coronary stents unless at risk for dangerous bleeding

## Slide 61
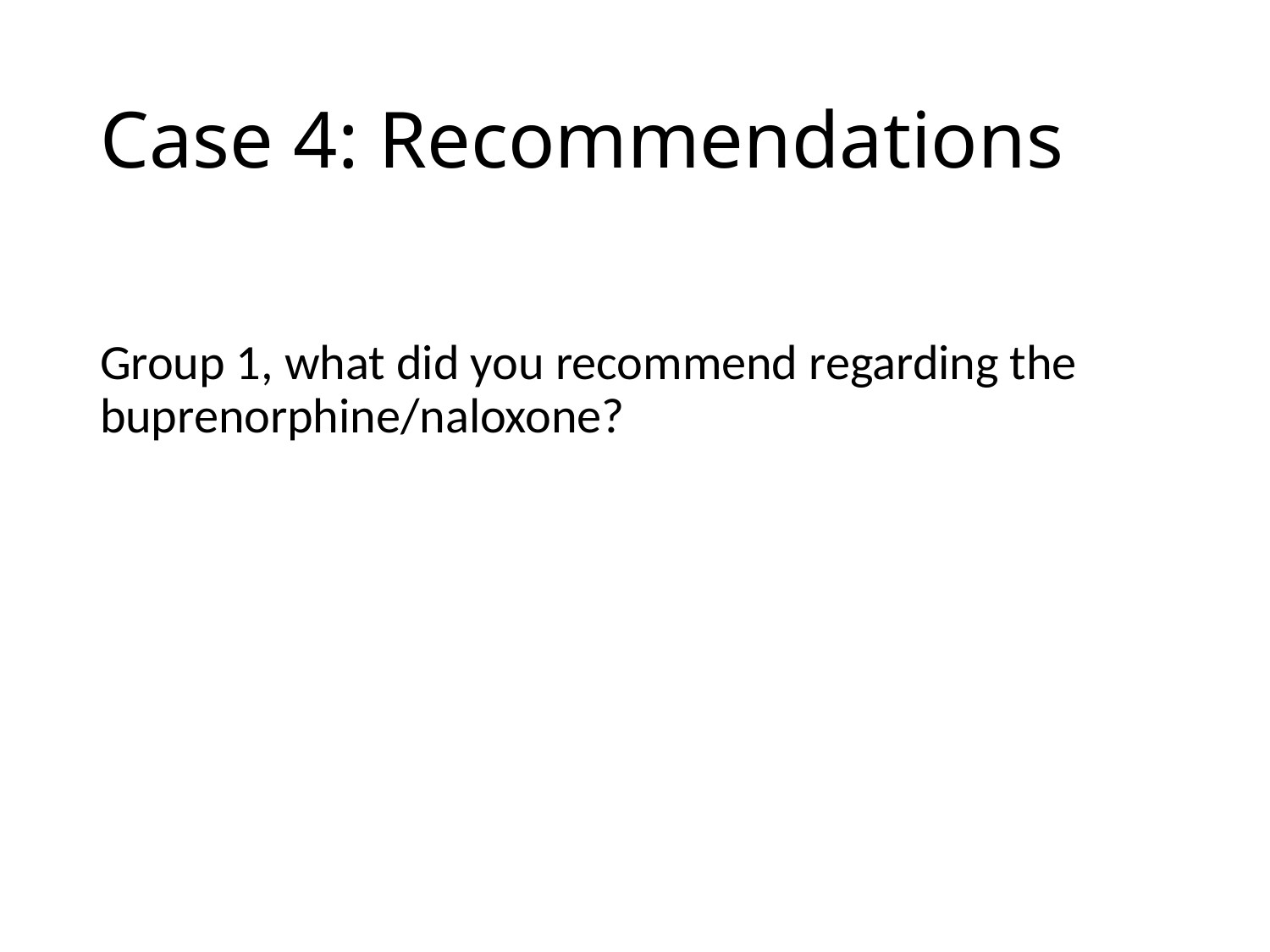

# Case 4: Recommendations
Group 1, what did you recommend regarding the buprenorphine/naloxone?

## Slide 62
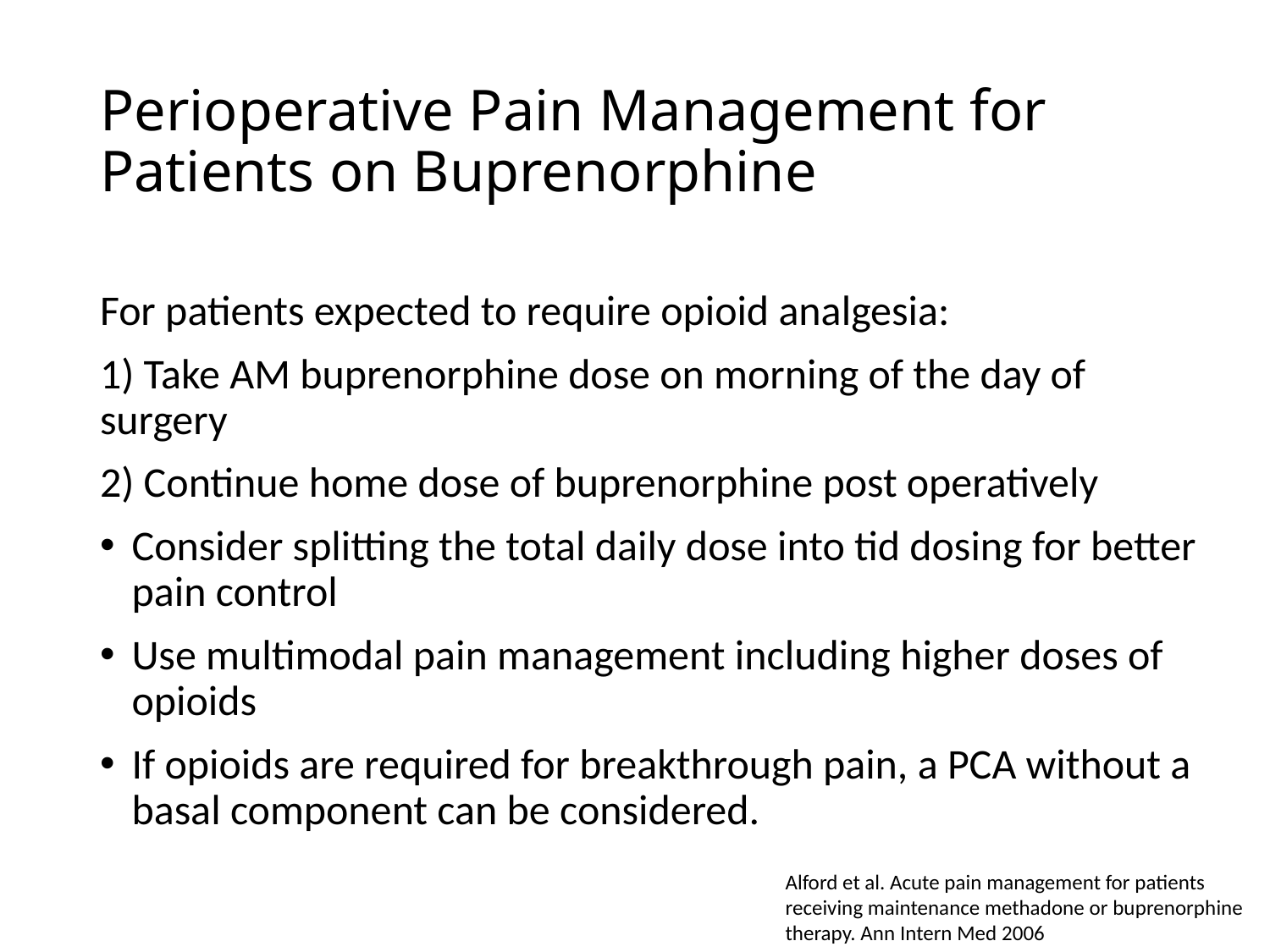

# Perioperative Pain Management for Patients on Buprenorphine
For patients expected to require opioid analgesia:
1) Take AM buprenorphine dose on morning of the day of surgery
2) Continue home dose of buprenorphine post operatively
Consider splitting the total daily dose into tid dosing for better pain control
Use multimodal pain management including higher doses of opioids
If opioids are required for breakthrough pain, a PCA without a basal component can be considered.
Alford et al. Acute pain management for patients receiving maintenance methadone or buprenorphine therapy. Ann Intern Med 2006

## Slide 63
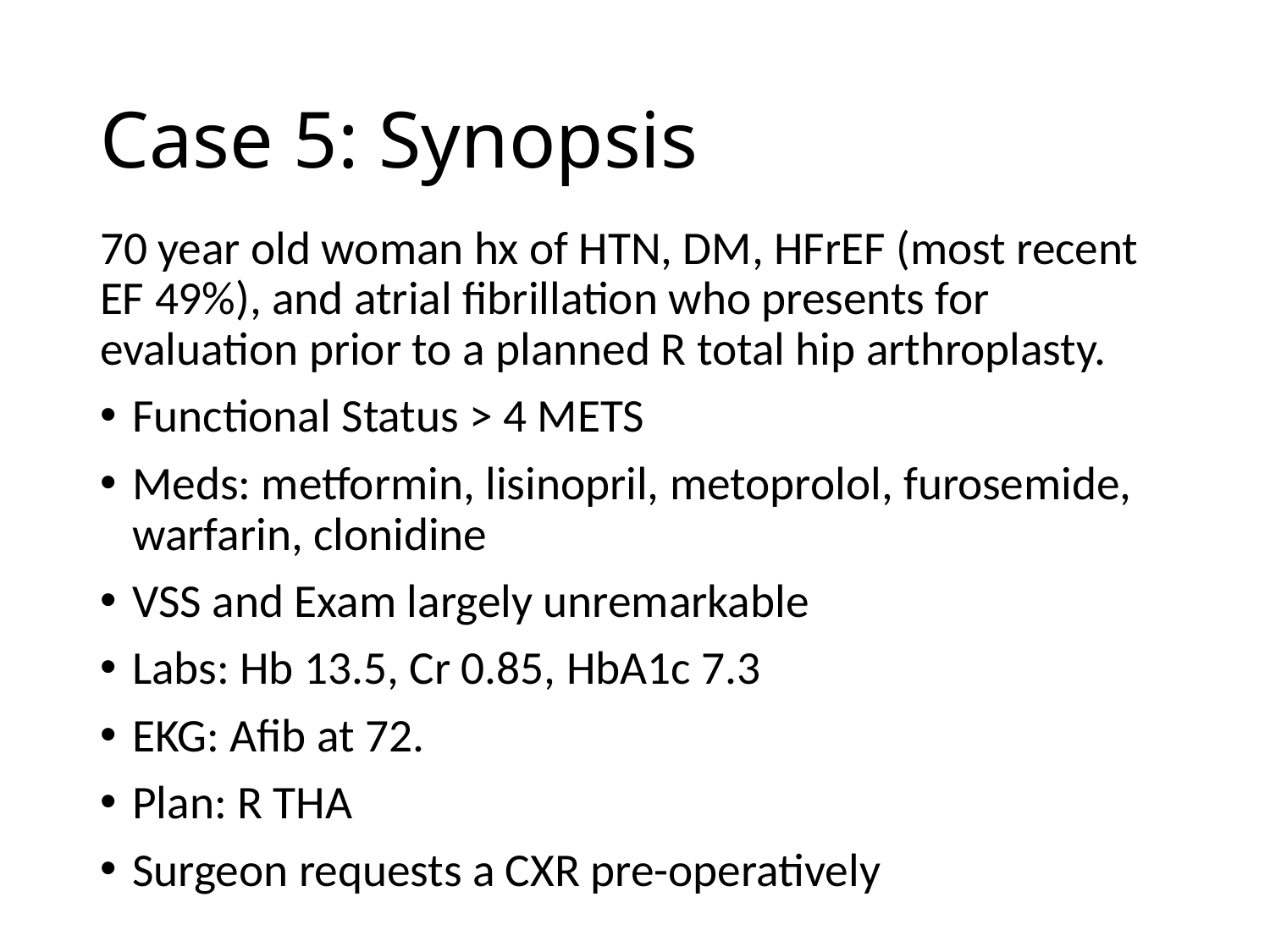

# Case 5: Synopsis
70 year old woman hx of HTN, DM, HFrEF (most recent EF 49%), and atrial fibrillation who presents for evaluation prior to a planned R total hip arthroplasty.
Functional Status > 4 METS
Meds: metformin, lisinopril, metoprolol, furosemide, warfarin, clonidine
VSS and Exam largely unremarkable
Labs: Hb 13.5, Cr 0.85, HbA1c 7.3
EKG: Afib at 72.
Plan: R THA
Surgeon requests a CXR pre-operatively

## Slide 64
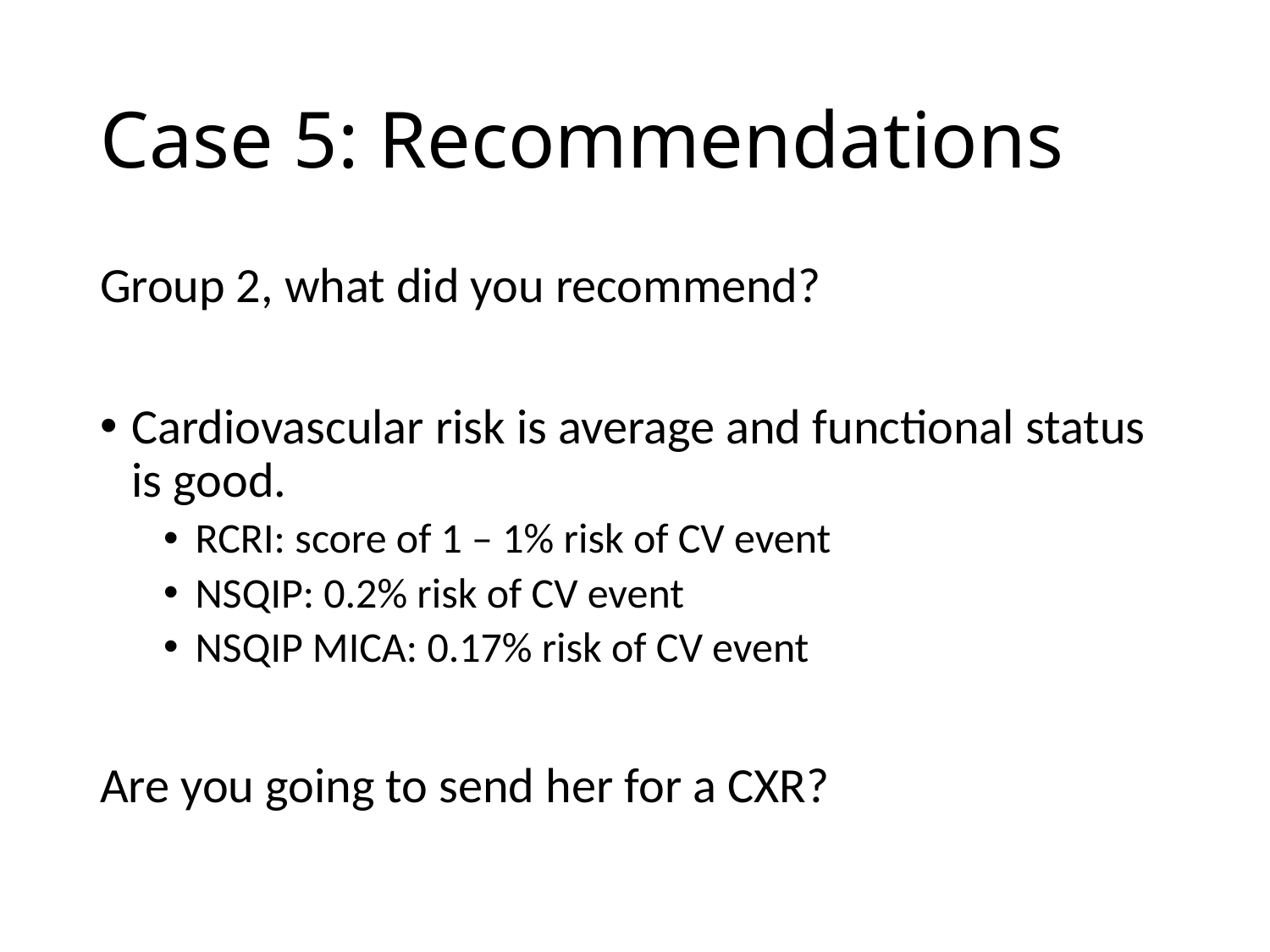

# Case 5: Recommendations
Group 2, what did you recommend?
Cardiovascular risk is average and functional status is good.
RCRI: score of 1 – 1% risk of CV event
NSQIP: 0.2% risk of CV event
NSQIP MICA: 0.17% risk of CV event
Are you going to send her for a CXR?

## Slide 65
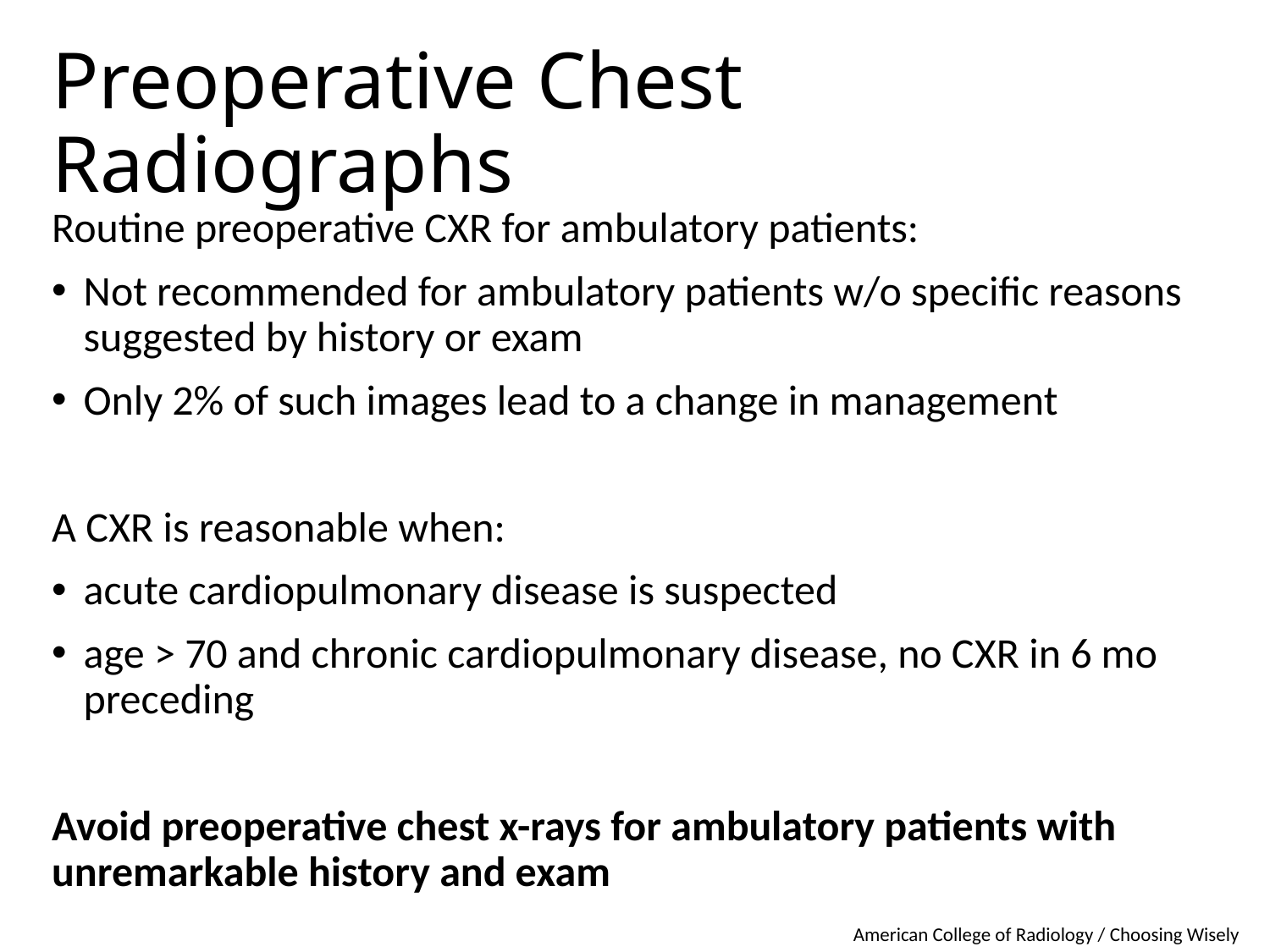

# Preoperative Chest Radiographs
Routine preoperative CXR for ambulatory patients:
Not recommended for ambulatory patients w/o specific reasons suggested by history or exam
Only 2% of such images lead to a change in management
A CXR is reasonable when:
acute cardiopulmonary disease is suspected
age > 70 and chronic cardiopulmonary disease, no CXR in 6 mo preceding
Avoid preoperative chest x-rays for ambulatory patients with unremarkable history and exam
American College of Radiology / Choosing Wisely

## Slide 66
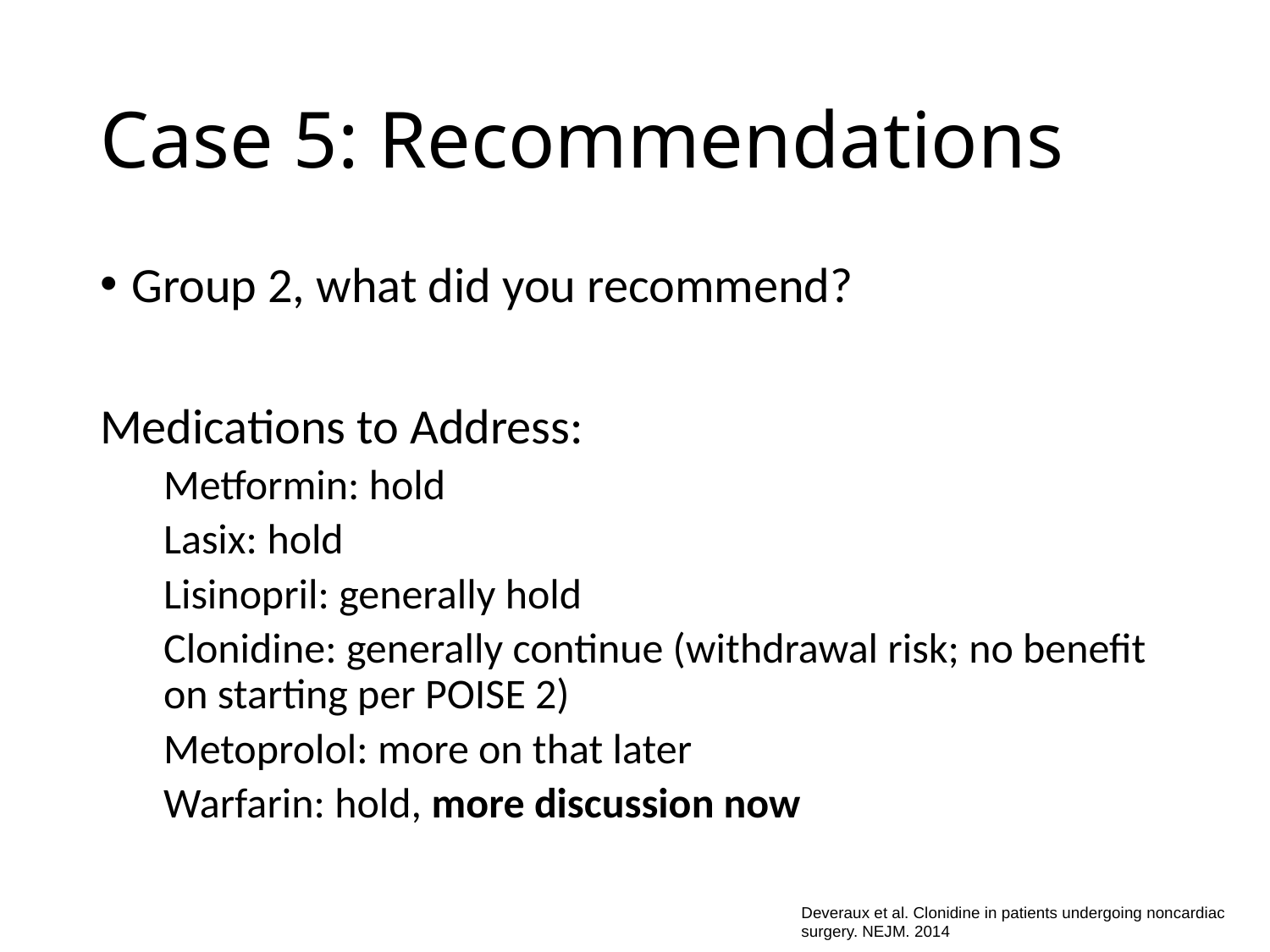

# Case 5: Recommendations
Group 2, what did you recommend?
Medications to Address:
Metformin: hold
Lasix: hold
Lisinopril: generally hold
Clonidine: generally continue (withdrawal risk; no benefit on starting per POISE 2)
Metoprolol: more on that later
Warfarin: hold, more discussion now
Deveraux et al. Clonidine in patients undergoing noncardiac surgery. NEJM. 2014

## Slide 67
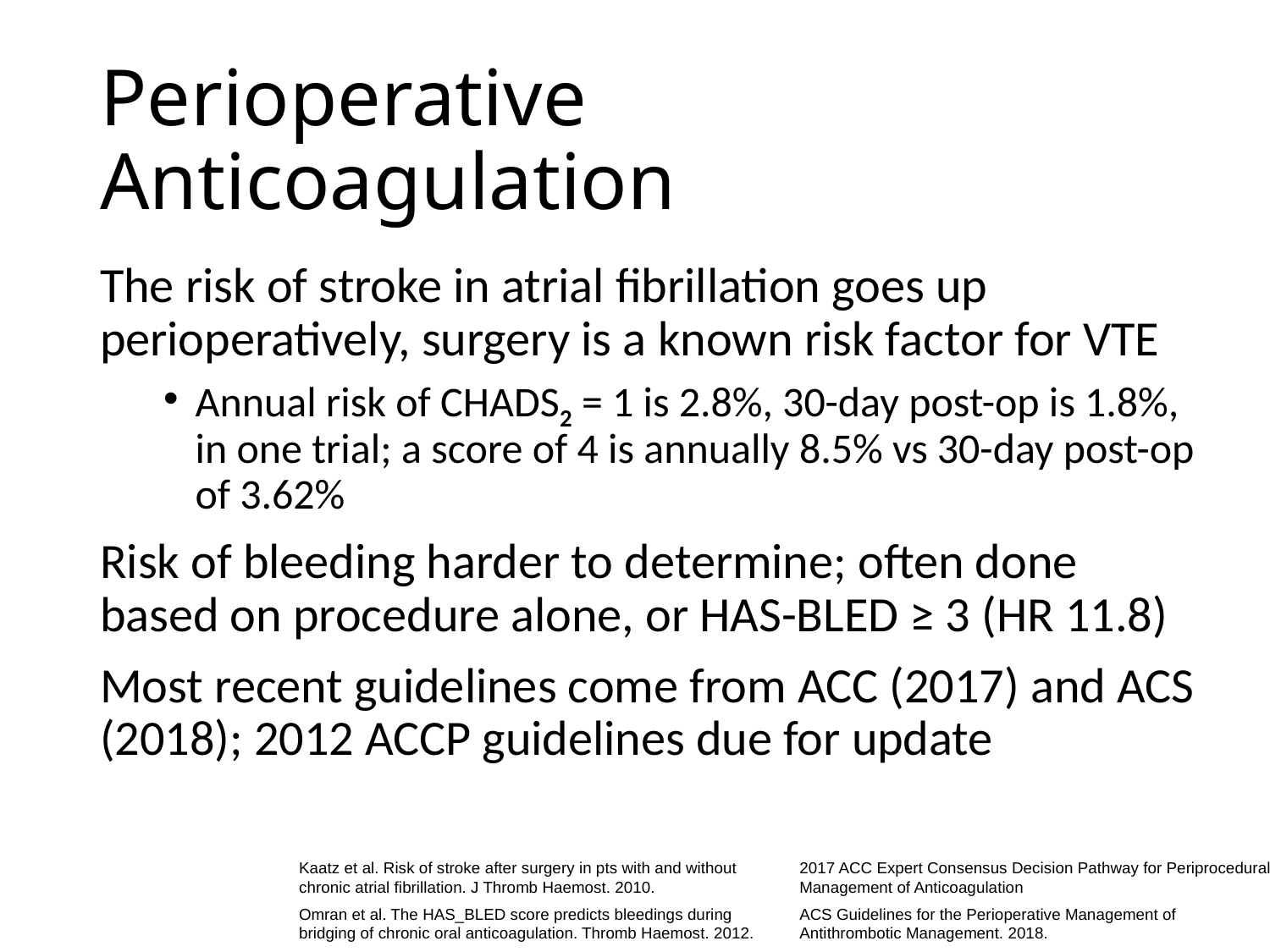

# Perioperative Anticoagulation
The risk of stroke in atrial fibrillation goes up perioperatively, surgery is a known risk factor for VTE
Annual risk of CHADS2 = 1 is 2.8%, 30-day post-op is 1.8%, in one trial; a score of 4 is annually 8.5% vs 30-day post-op of 3.62%
Risk of bleeding harder to determine; often done based on procedure alone, or HAS-BLED ≥ 3 (HR 11.8)
Most recent guidelines come from ACC (2017) and ACS (2018); 2012 ACCP guidelines due for update
2017 ACC Expert Consensus Decision Pathway for Periprocedural Management of Anticoagulation
ACS Guidelines for the Perioperative Management of Antithrombotic Management. 2018.
Kaatz et al. Risk of stroke after surgery in pts with and without chronic atrial fibrillation. J Thromb Haemost. 2010.
Omran et al. The HAS_BLED score predicts bleedings during bridging of chronic oral anticoagulation. Thromb Haemost. 2012.

## Slide 68
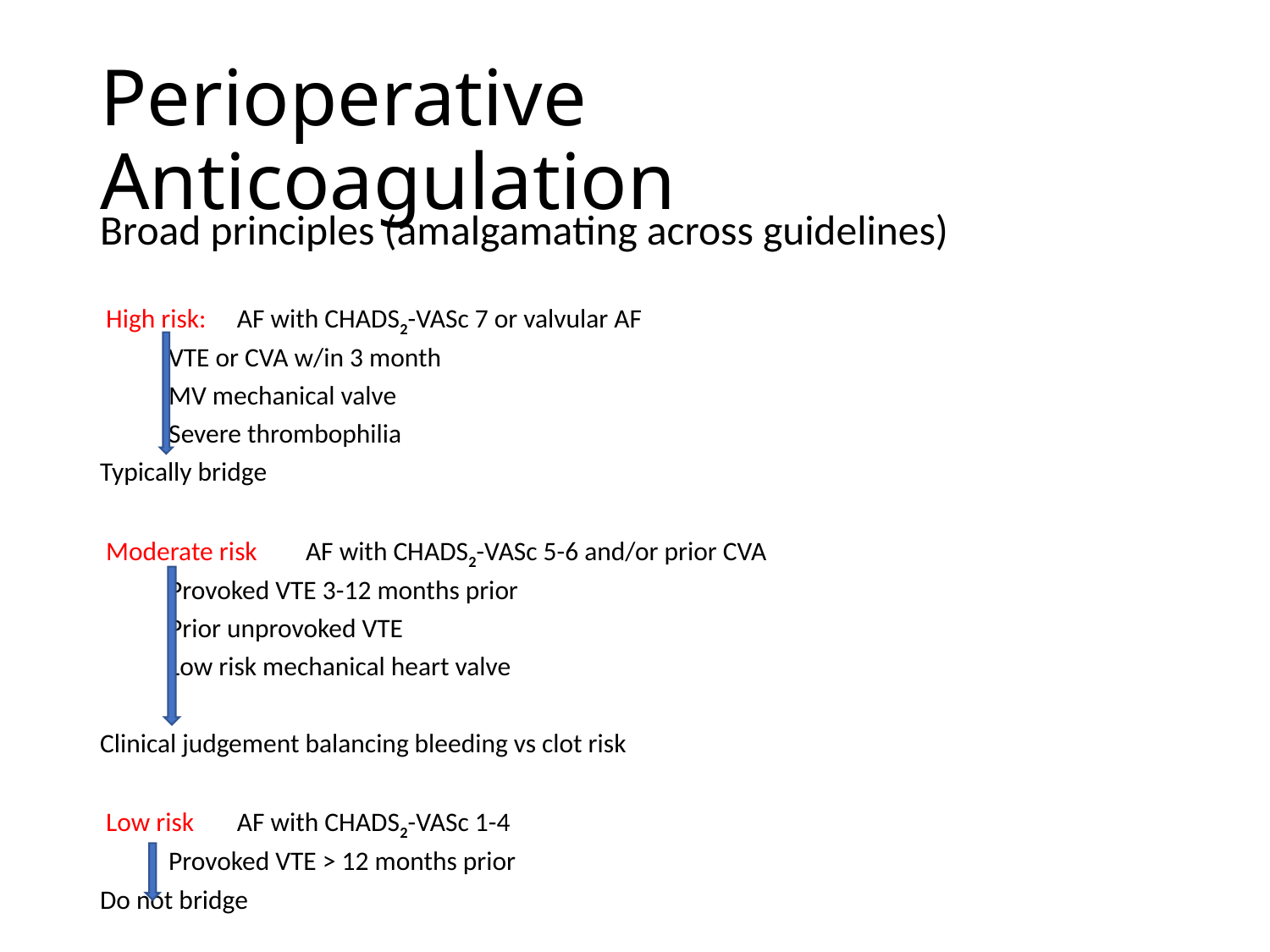

# Perioperative Anticoagulation
Broad principles (amalgamating across guidelines)
 High risk:		AF with CHADS2-VASc 7 or valvular AF
		VTE or CVA w/in 3 month
		MV mechanical valve
		Severe thrombophilia
Typically bridge
 Moderate risk 	AF with CHADS2-VASc 5-6 and/or prior CVA
		Provoked VTE 3-12 months prior
		Prior unprovoked VTE
		Low risk mechanical heart valve
Clinical judgement balancing bleeding vs clot risk
 Low risk 		AF with CHADS2-VASc 1-4
		Provoked VTE > 12 months prior
Do not bridge

## Slide 69
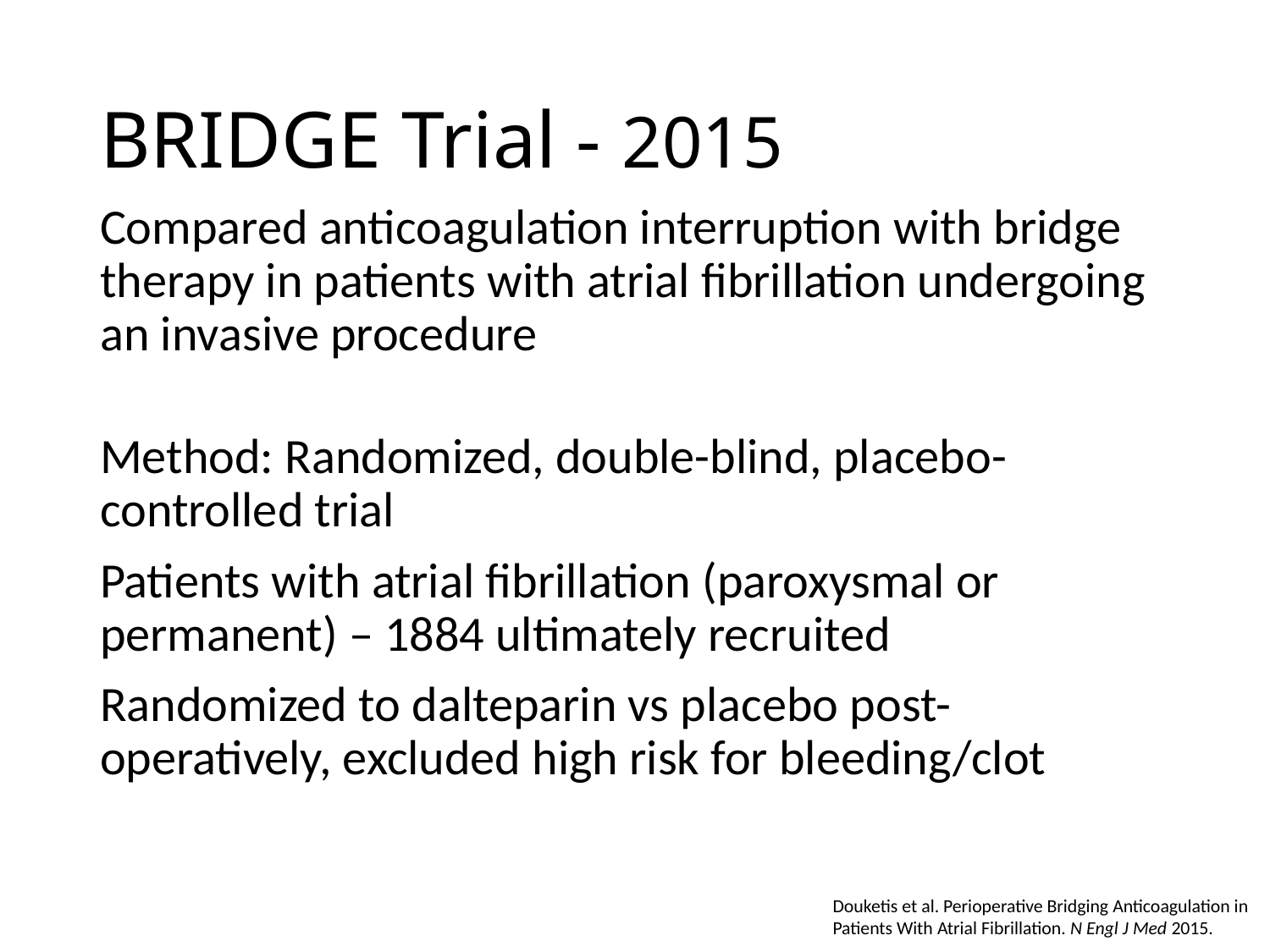

# BRIDGE Trial - 2015
Compared anticoagulation interruption with bridge therapy in patients with atrial fibrillation undergoing an invasive procedure
Method: Randomized, double-blind, placebo-controlled trial
Patients with atrial fibrillation (paroxysmal or permanent) – 1884 ultimately recruited
Randomized to dalteparin vs placebo post-operatively, excluded high risk for bleeding/clot
Douketis et al. Perioperative Bridging Anticoagulation in Patients With Atrial Fibrillation. N Engl J Med 2015.

## Slide 70
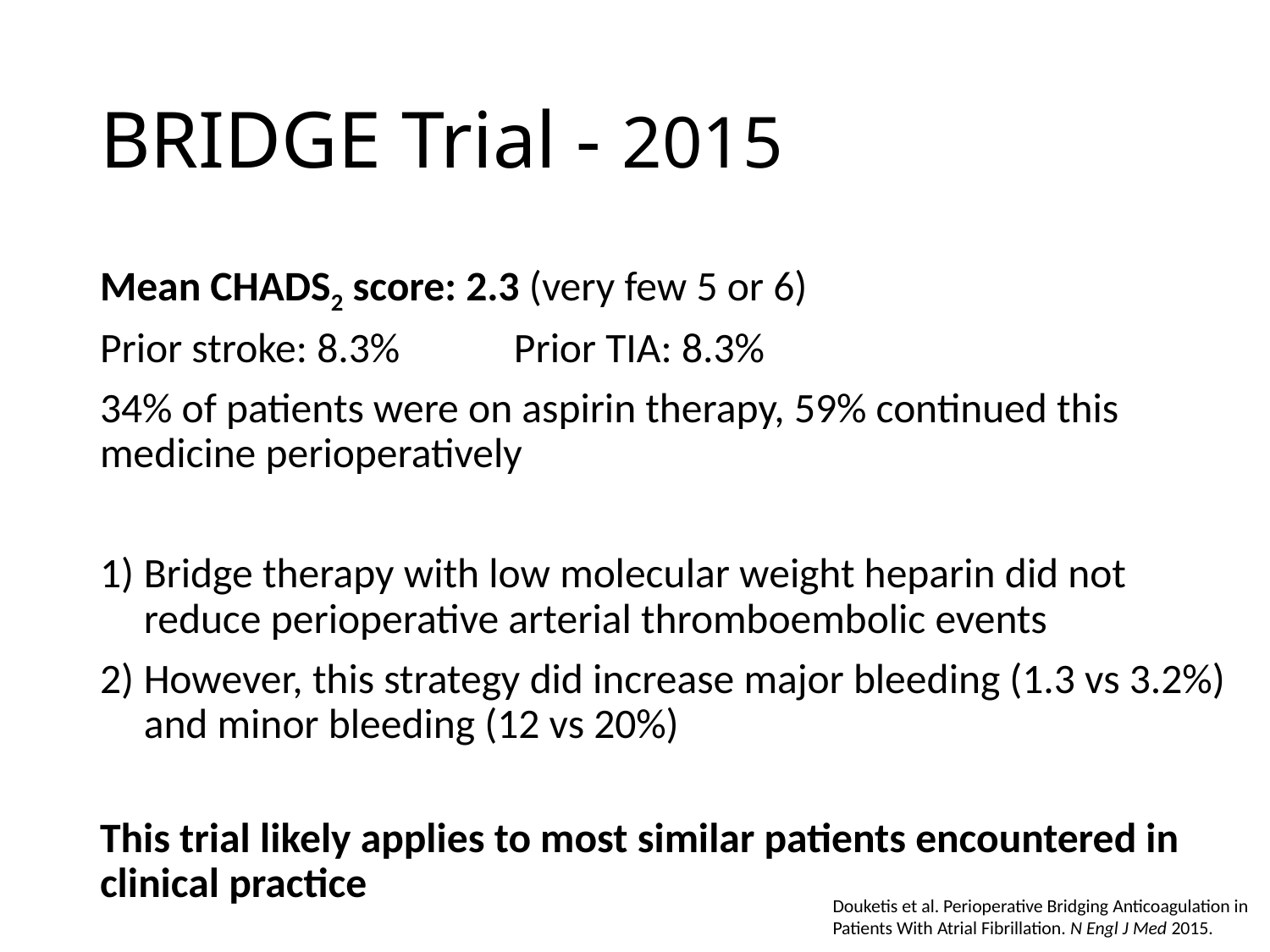

# BRIDGE Trial - 2015
Mean CHADS2 score: 2.3 (very few 5 or 6)
Prior stroke: 8.3% Prior TIA: 8.3%
34% of patients were on aspirin therapy, 59% continued this medicine perioperatively
Bridge therapy with low molecular weight heparin did not reduce perioperative arterial thromboembolic events
However, this strategy did increase major bleeding (1.3 vs 3.2%) and minor bleeding (12 vs 20%)
This trial likely applies to most similar patients encountered in clinical practice
Douketis et al. Perioperative Bridging Anticoagulation in Patients With Atrial Fibrillation. N Engl J Med 2015.

## Slide 71
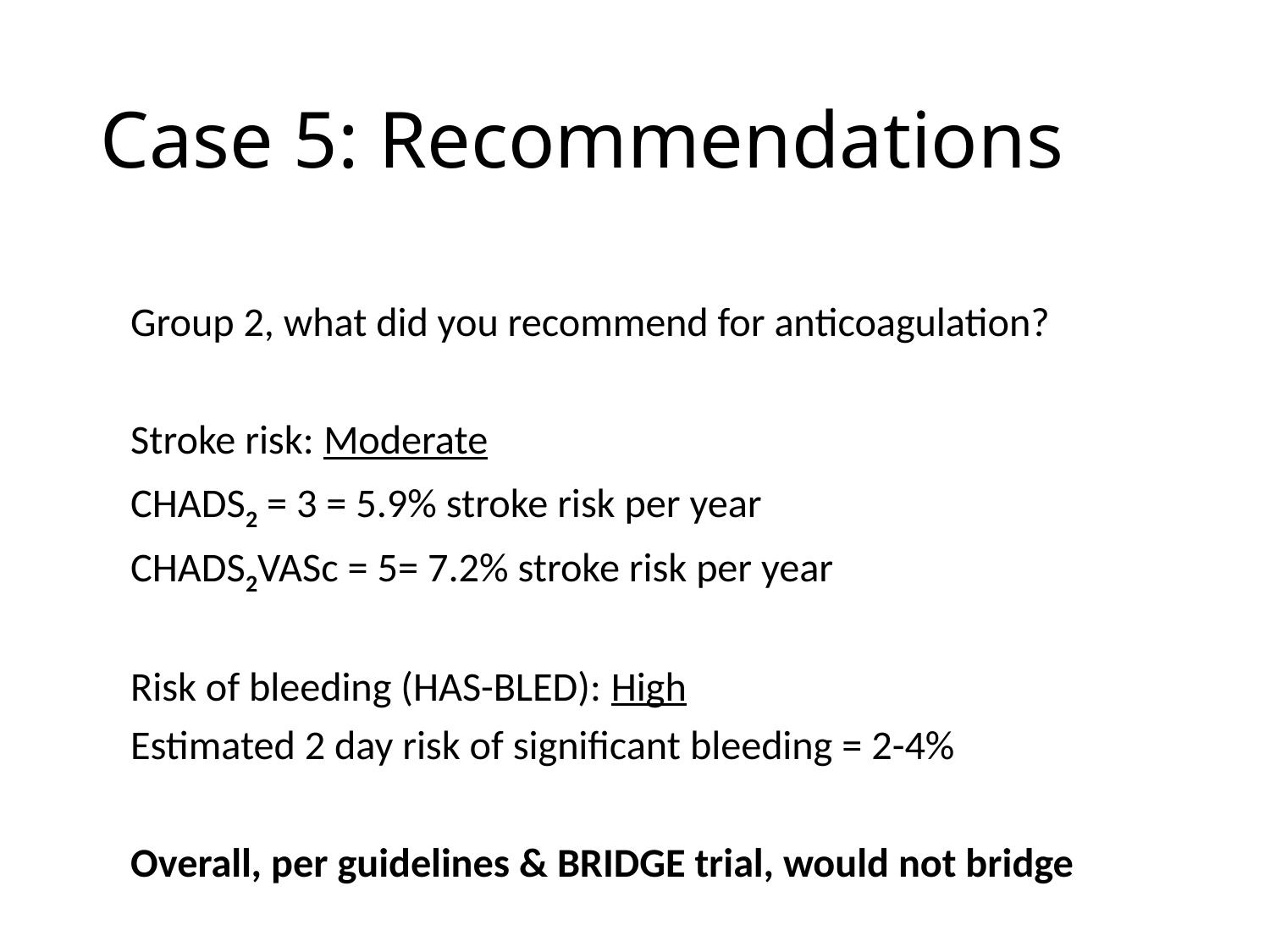

# Case 5: Recommendations
Group 2, what did you recommend for anticoagulation?
Stroke risk: Moderate
CHADS2 = 3 = 5.9% stroke risk per year
CHADS2VASc = 5= 7.2% stroke risk per year
Risk of bleeding (HAS-BLED): High
Estimated 2 day risk of significant bleeding = 2-4%
Overall, per guidelines & BRIDGE trial, would not bridge

## Slide 72
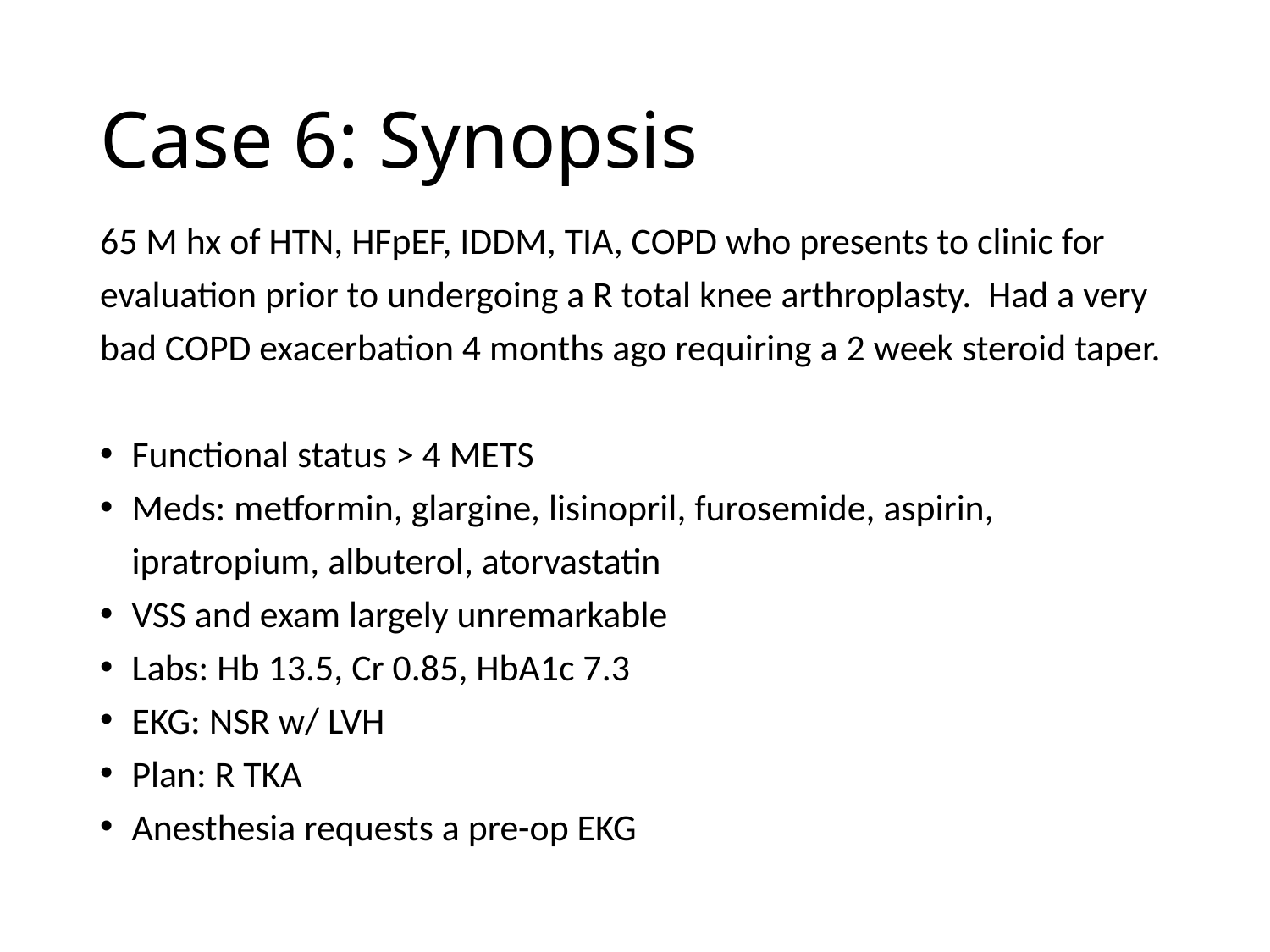

# Case 6: Synopsis
65 M hx of HTN, HFpEF, IDDM, TIA, COPD who presents to clinic for evaluation prior to undergoing a R total knee arthroplasty. Had a very bad COPD exacerbation 4 months ago requiring a 2 week steroid taper.
Functional status > 4 METS
Meds: metformin, glargine, lisinopril, furosemide, aspirin, ipratropium, albuterol, atorvastatin
VSS and exam largely unremarkable
Labs: Hb 13.5, Cr 0.85, HbA1c 7.3
EKG: NSR w/ LVH
Plan: R TKA
Anesthesia requests a pre-op EKG

## Slide 73
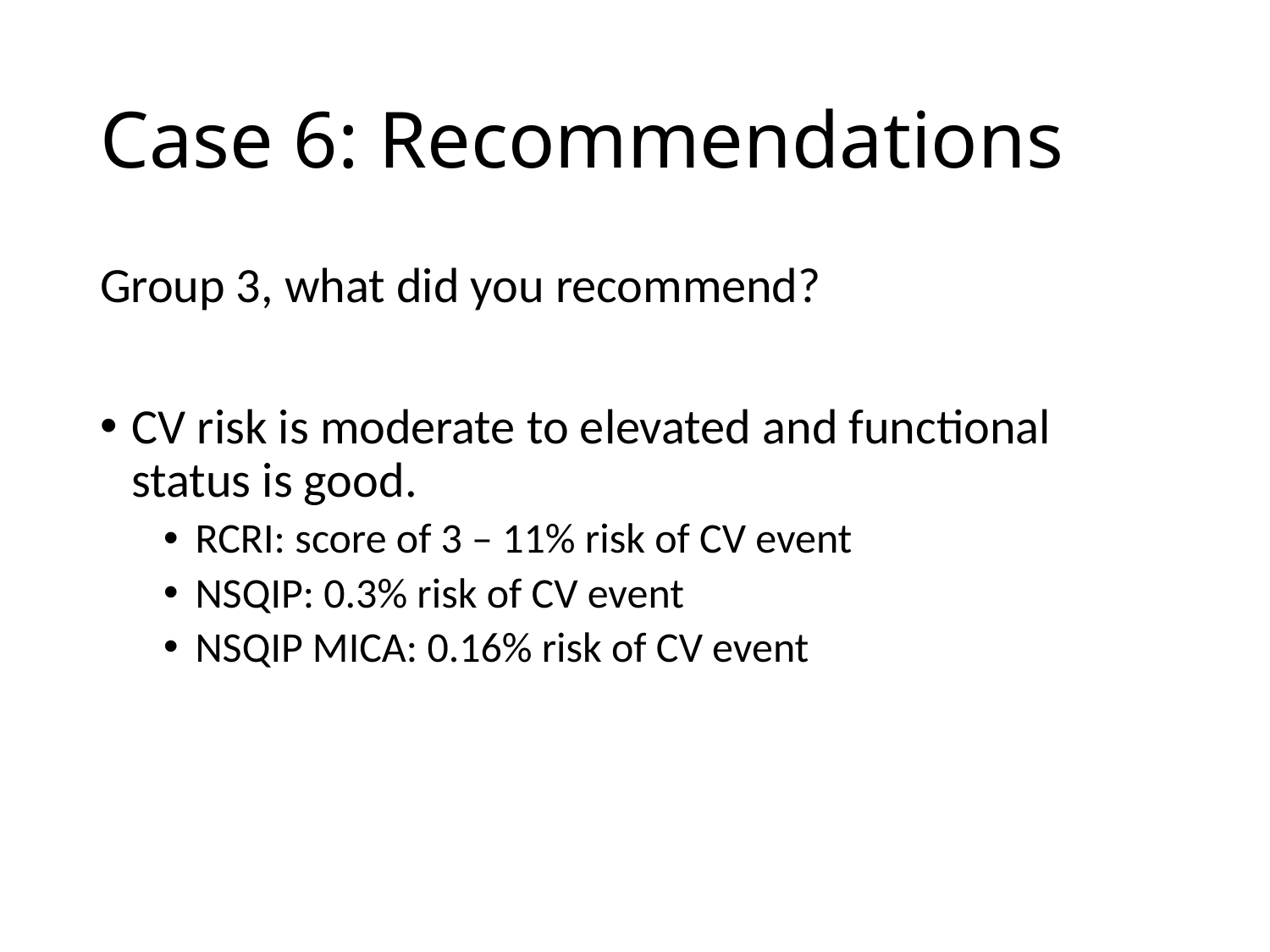

# Case 6: Recommendations
Group 3, what did you recommend?
CV risk is moderate to elevated and functional status is good.
RCRI: score of 3 – 11% risk of CV event
NSQIP: 0.3% risk of CV event
NSQIP MICA: 0.16% risk of CV event

## Slide 74
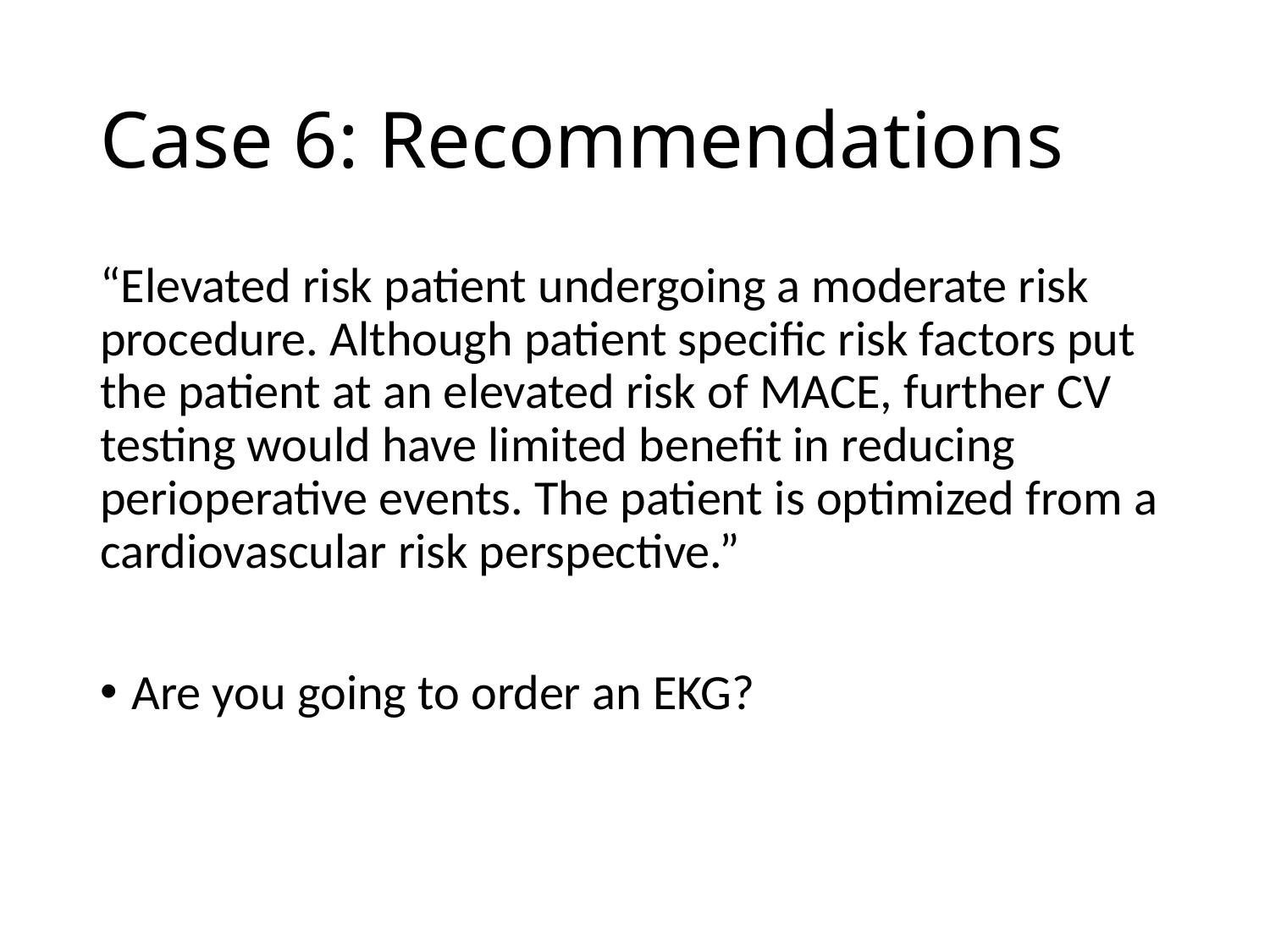

# Case 6: Recommendations
“Elevated risk patient undergoing a moderate risk procedure. Although patient specific risk factors put the patient at an elevated risk of MACE, further CV testing would have limited benefit in reducing perioperative events. The patient is optimized from a cardiovascular risk perspective.”
Are you going to order an EKG?

## Slide 75
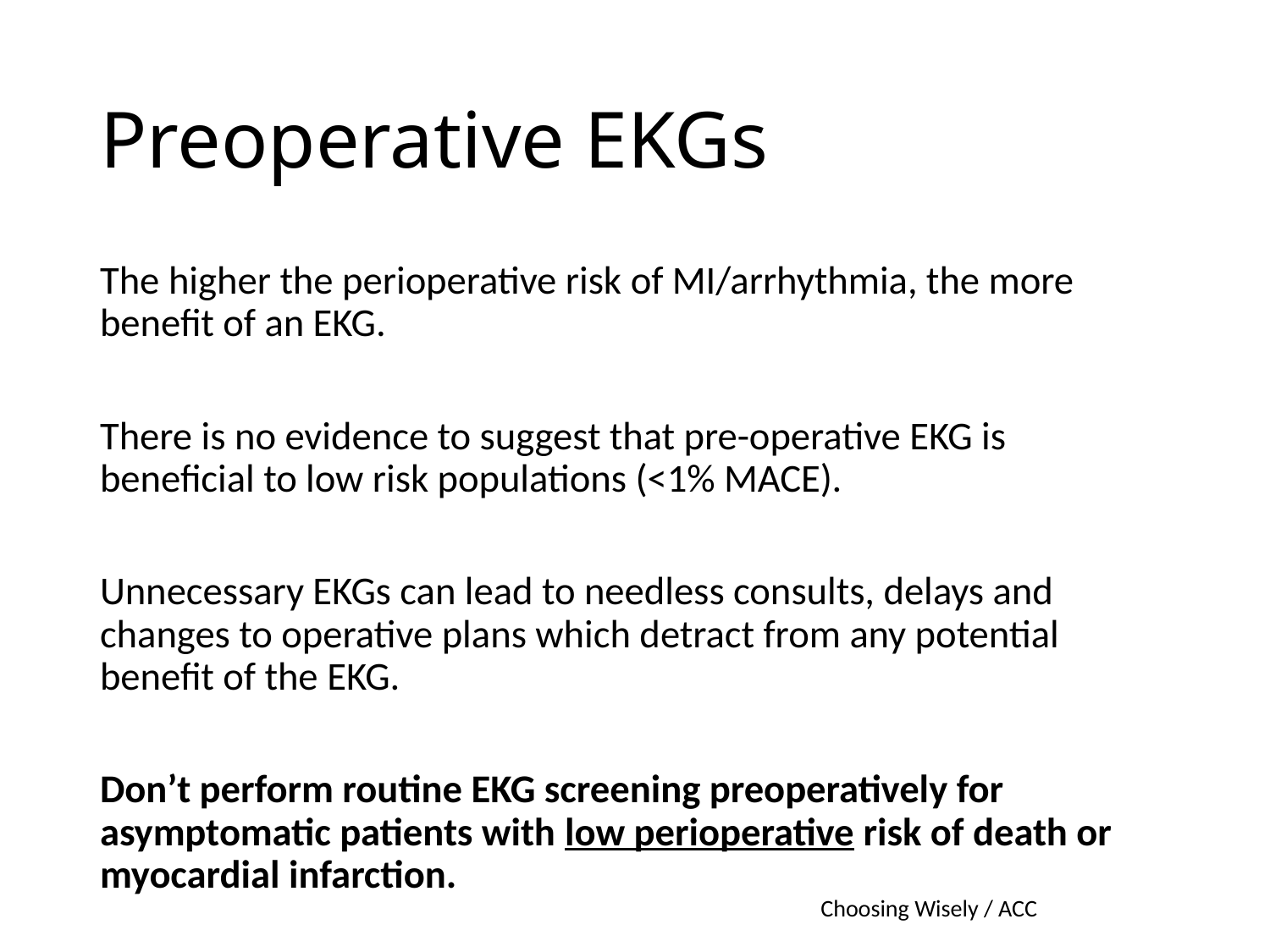

# Preoperative EKGs
The higher the perioperative risk of MI/arrhythmia, the more benefit of an EKG.
There is no evidence to suggest that pre-operative EKG is beneficial to low risk populations (<1% MACE).
Unnecessary EKGs can lead to needless consults, delays and changes to operative plans which detract from any potential benefit of the EKG.
Don’t perform routine EKG screening preoperatively for asymptomatic patients with low perioperative risk of death or myocardial infarction.
Choosing Wisely / ACC

## Slide 76
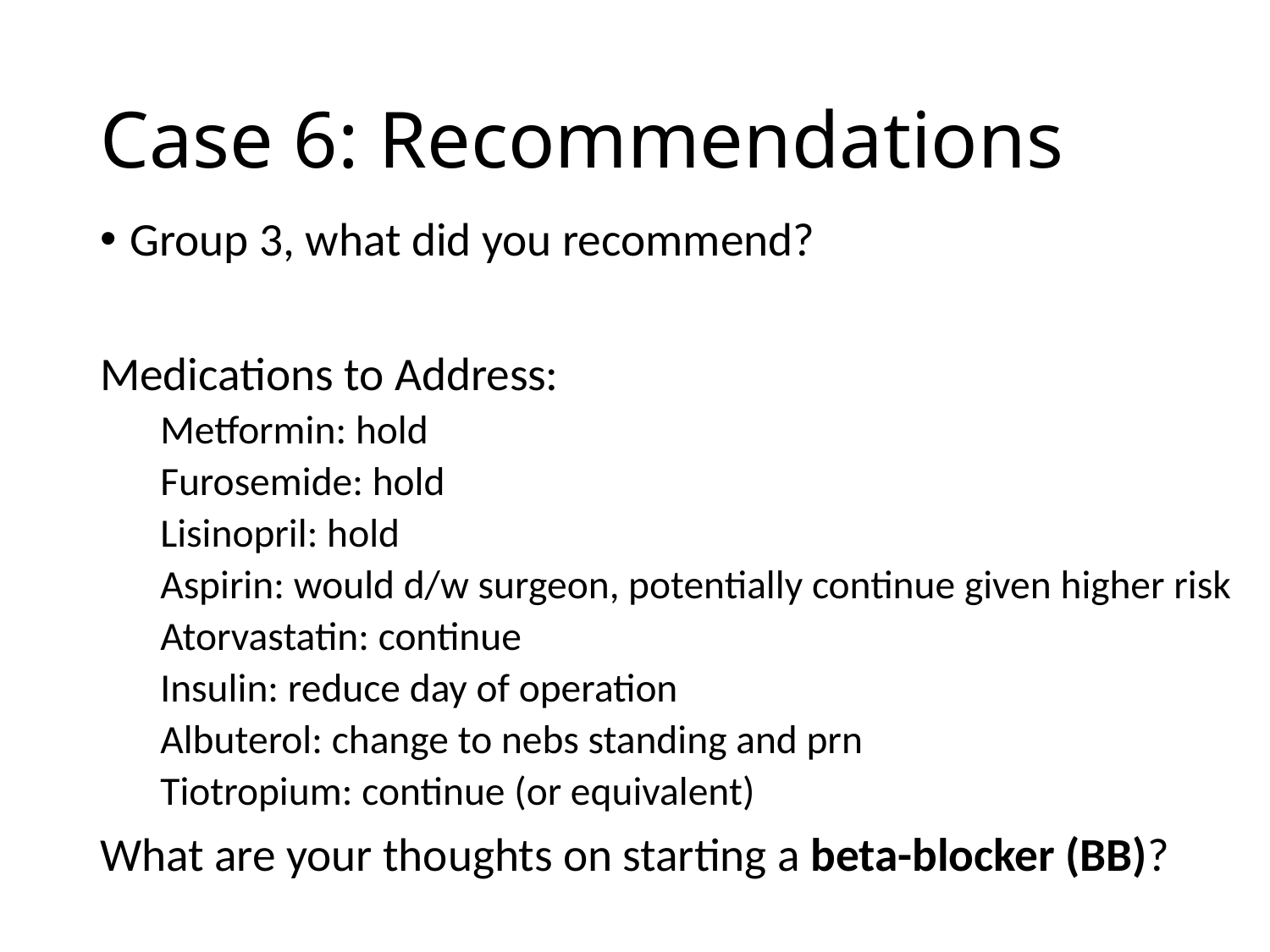

# Case 6: Recommendations
Group 3, what did you recommend?
Medications to Address:
Metformin: hold
Furosemide: hold
Lisinopril: hold
Aspirin: would d/w surgeon, potentially continue given higher risk
Atorvastatin: continue
Insulin: reduce day of operation
Albuterol: change to nebs standing and prn
Tiotropium: continue (or equivalent)
What are your thoughts on starting a beta-blocker (BB)?

## Slide 77
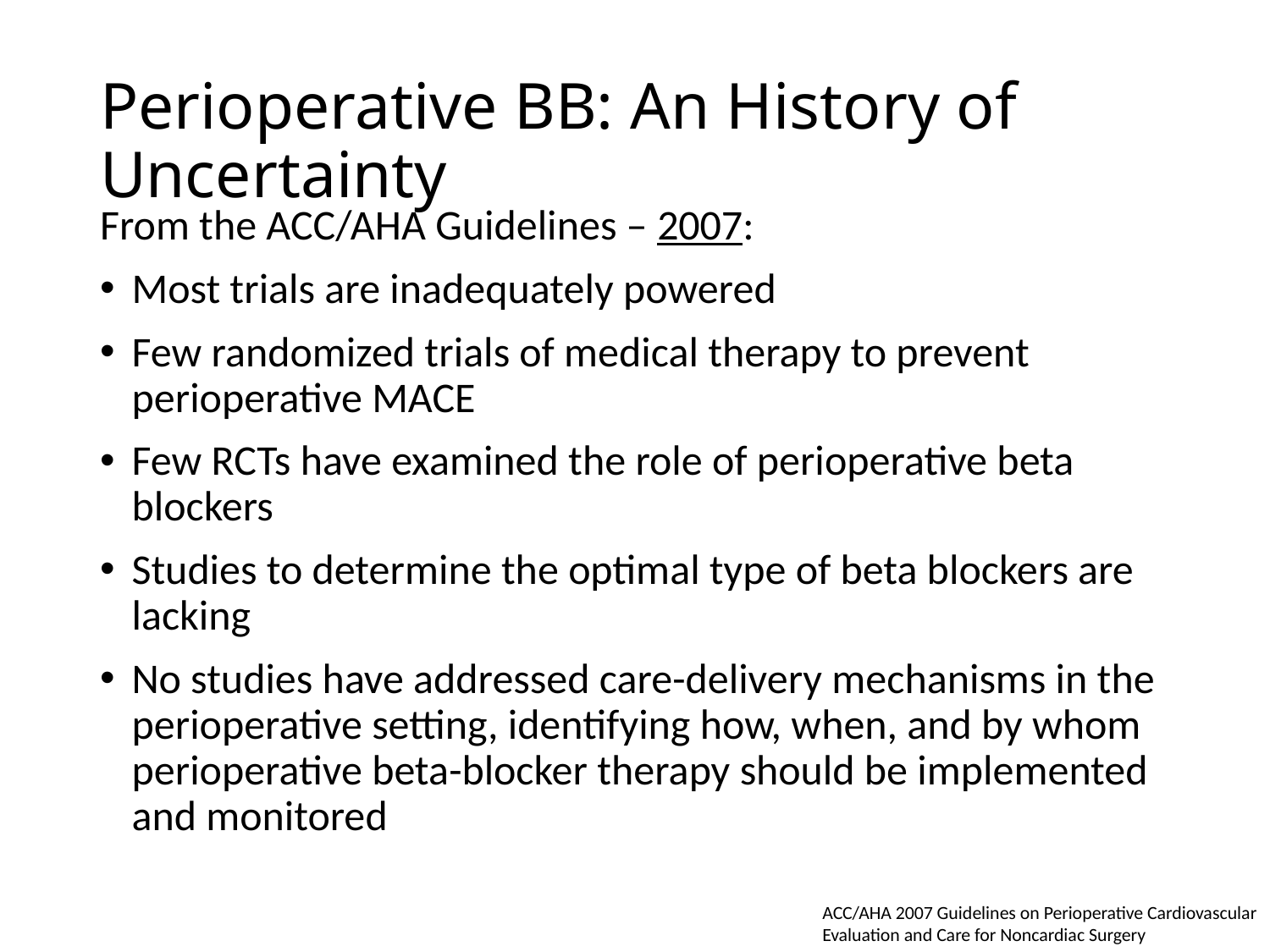

# Perioperative BB: An History of Uncertainty
From the ACC/AHA Guidelines – 2007:
Most trials are inadequately powered
Few randomized trials of medical therapy to prevent perioperative MACE
Few RCTs have examined the role of perioperative beta blockers
Studies to determine the optimal type of beta blockers are lacking
No studies have addressed care-delivery mechanisms in the perioperative setting, identifying how, when, and by whom perioperative beta-blocker therapy should be implemented and monitored
ACC/AHA 2007 Guidelines on Perioperative Cardiovascular Evaluation and Care for Noncardiac Surgery

## Slide 78
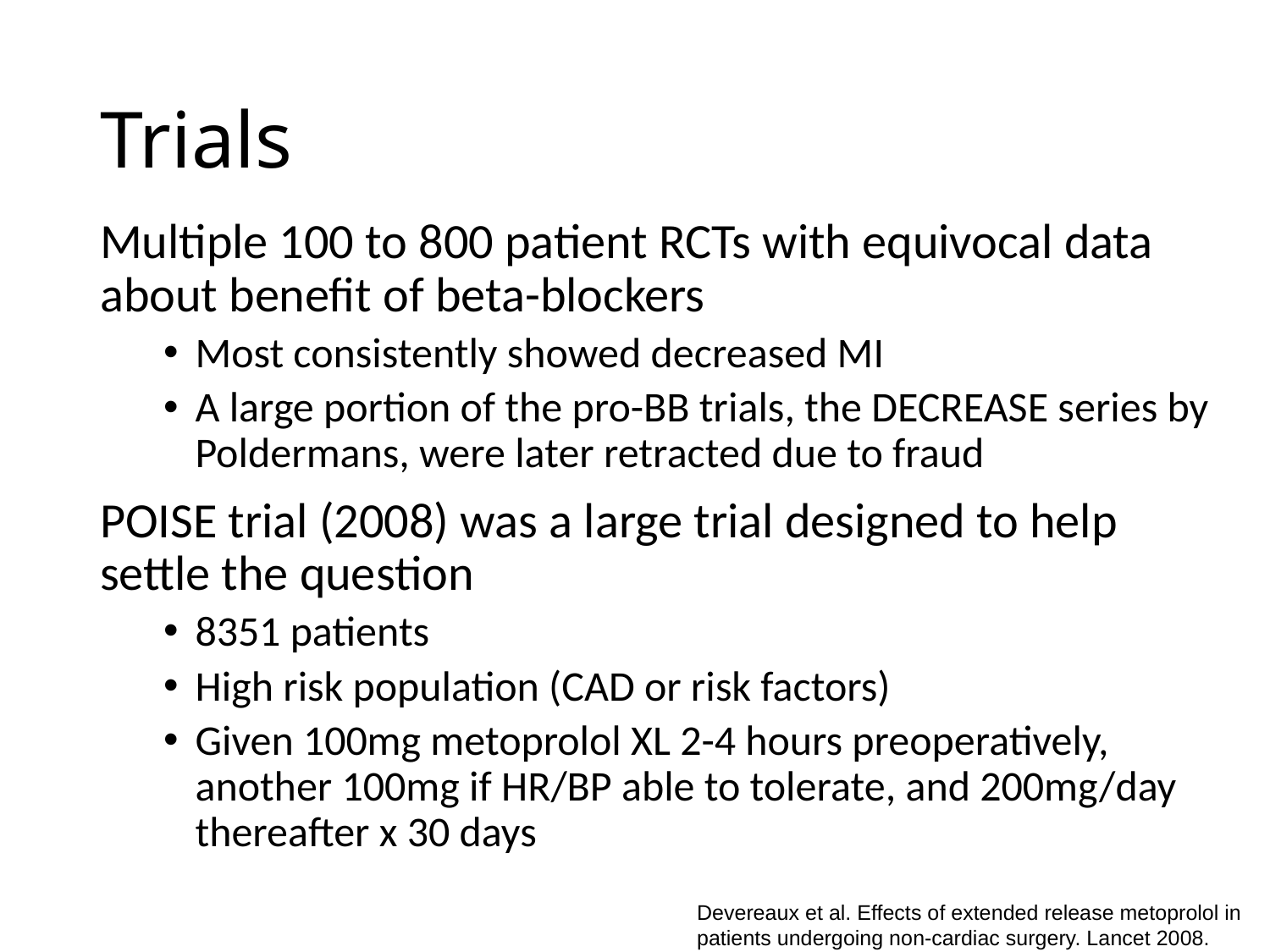

# Trials
Multiple 100 to 800 patient RCTs with equivocal data about benefit of beta-blockers
Most consistently showed decreased MI
A large portion of the pro-BB trials, the DECREASE series by Poldermans, were later retracted due to fraud
POISE trial (2008) was a large trial designed to help settle the question
8351 patients
High risk population (CAD or risk factors)
Given 100mg metoprolol XL 2-4 hours preoperatively, another 100mg if HR/BP able to tolerate, and 200mg/day thereafter x 30 days
Devereaux et al. Effects of extended release metoprolol in patients undergoing non-cardiac surgery. Lancet 2008.

## Slide 79
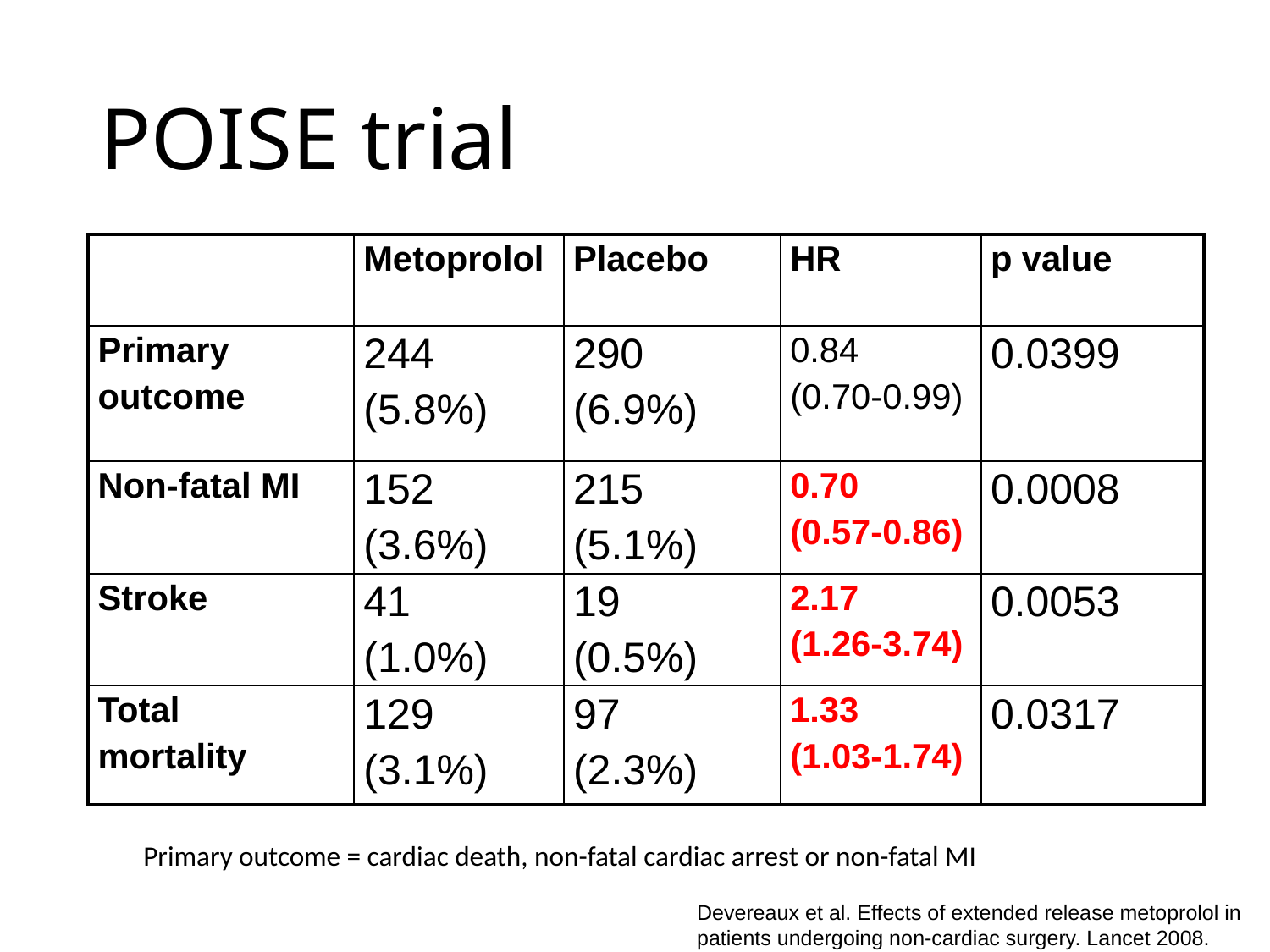

# POISE trial
| | Metoprolol | Placebo | HR | p value |
| --- | --- | --- | --- | --- |
| Primary outcome | 244 (5.8%) | 290 (6.9%) | 0.84 (0.70-0.99) | 0.0399 |
| Non-fatal MI | 152 (3.6%) | 215 (5.1%) | 0.70 (0.57-0.86) | 0.0008 |
| Stroke | 41 (1.0%) | 19 (0.5%) | 2.17 (1.26-3.74) | 0.0053 |
| Total mortality | 129 (3.1%) | 97 (2.3%) | 1.33 (1.03-1.74) | 0.0317 |
Primary outcome = cardiac death, non-fatal cardiac arrest or non-fatal MI
Devereaux et al. Effects of extended release metoprolol in patients undergoing non-cardiac surgery. Lancet 2008.

## Slide 80
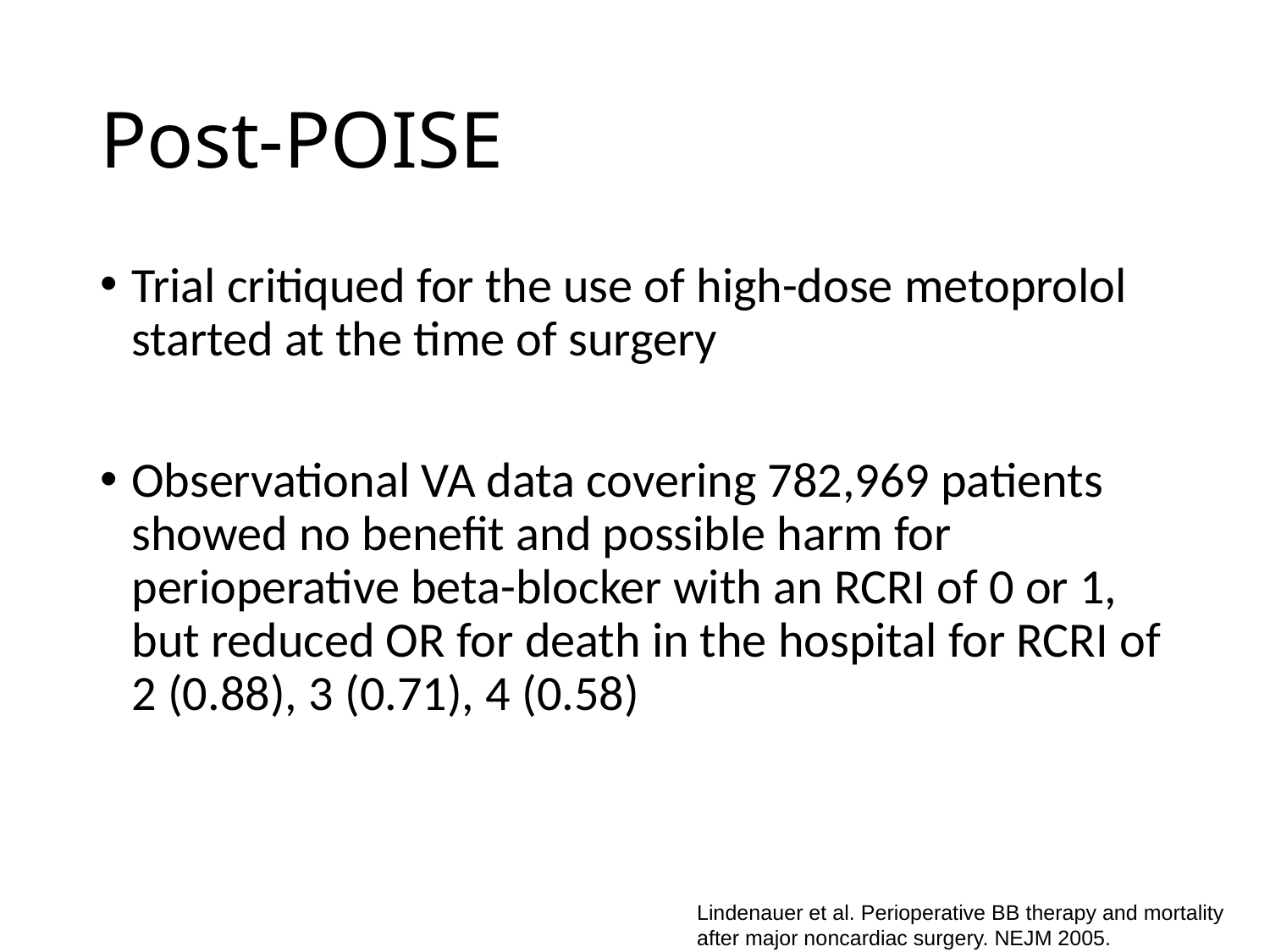

# Post-POISE
Trial critiqued for the use of high-dose metoprolol started at the time of surgery
Observational VA data covering 782,969 patients showed no benefit and possible harm for perioperative beta-blocker with an RCRI of 0 or 1, but reduced OR for death in the hospital for RCRI of 2 (0.88), 3 (0.71), 4 (0.58)
Lindenauer et al. Perioperative BB therapy and mortality after major noncardiac surgery. NEJM 2005.

## Slide 81
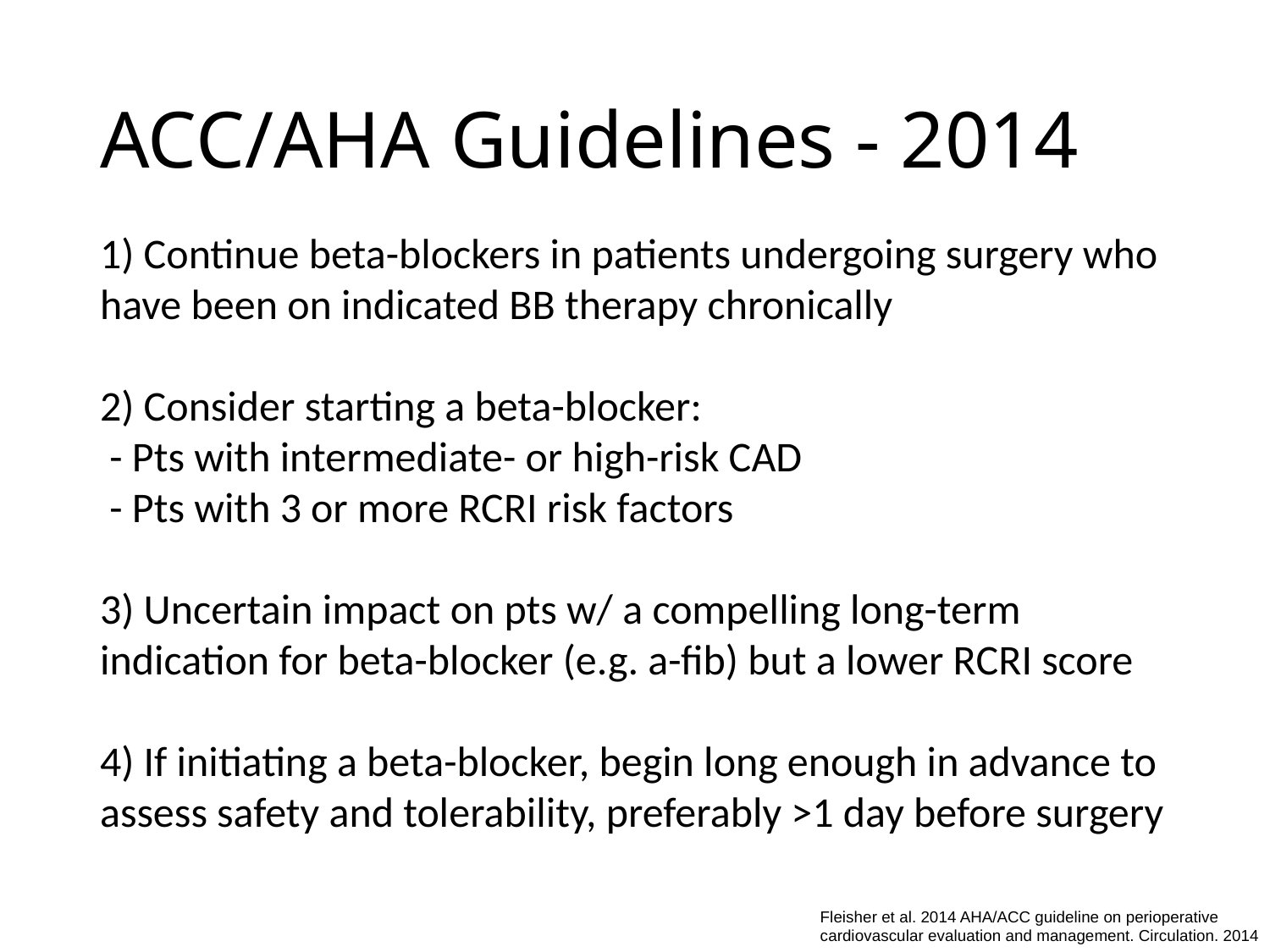

# ACC/AHA Guidelines - 2014
1) Continue beta-blockers in patients undergoing surgery who have been on indicated BB therapy chronically
2) Consider starting a beta-blocker:
 - Pts with intermediate- or high-risk CAD
 - Pts with 3 or more RCRI risk factors
3) Uncertain impact on pts w/ a compelling long-term indication for beta-blocker (e.g. a-fib) but a lower RCRI score
4) If initiating a beta-blocker, begin long enough in advance to assess safety and tolerability, preferably >1 day before surgery
Fleisher et al. 2014 AHA/ACC guideline on perioperative cardiovascular evaluation and management. Circulation. 2014

## Slide 82
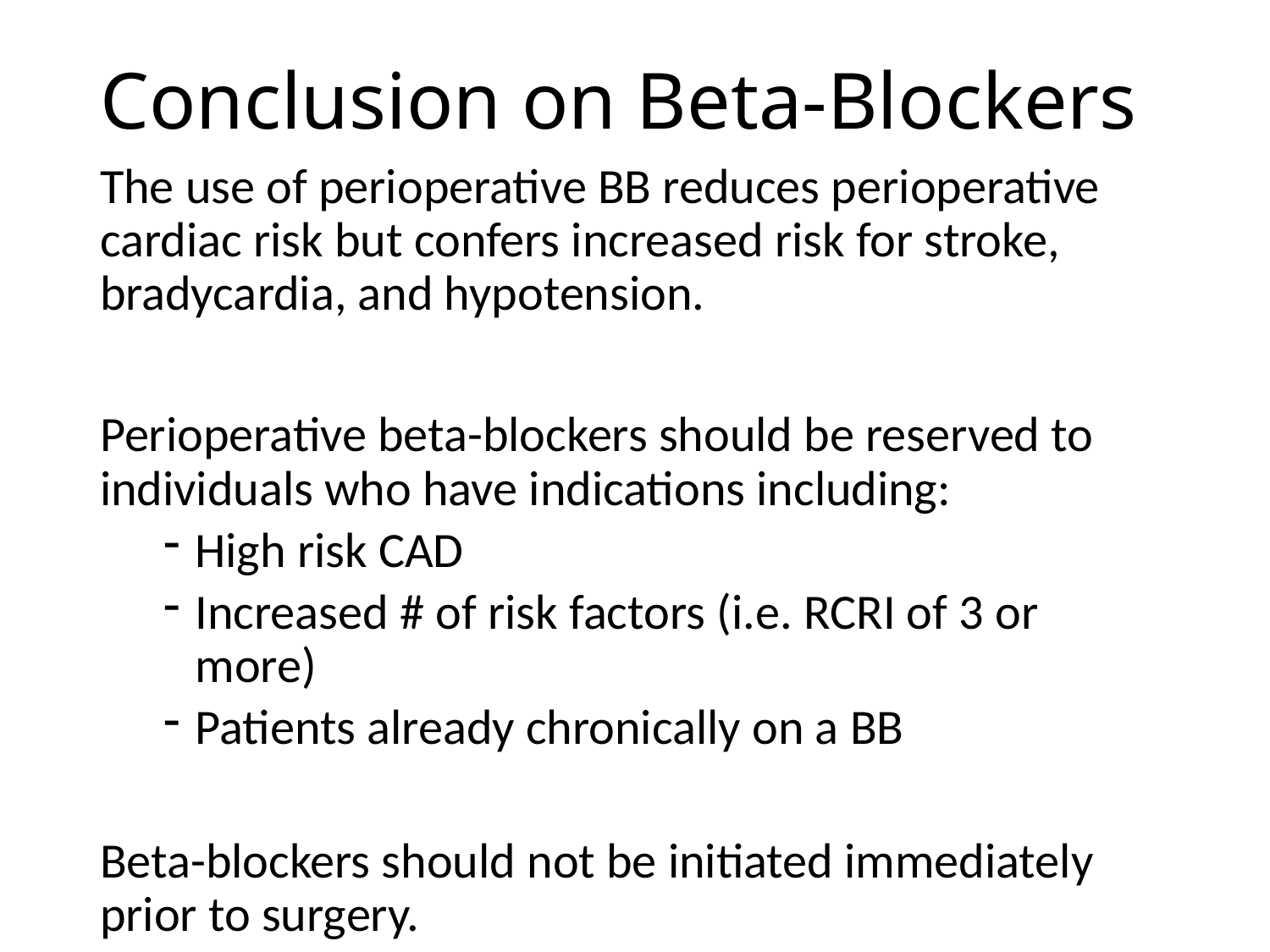

# Conclusion on Beta-Blockers
The use of perioperative BB reduces perioperative cardiac risk but confers increased risk for stroke, bradycardia, and hypotension.
Perioperative beta-blockers should be reserved to individuals who have indications including:
High risk CAD
Increased # of risk factors (i.e. RCRI of 3 or more)
Patients already chronically on a BB
Beta-blockers should not be initiated immediately prior to surgery.

## Slide 83
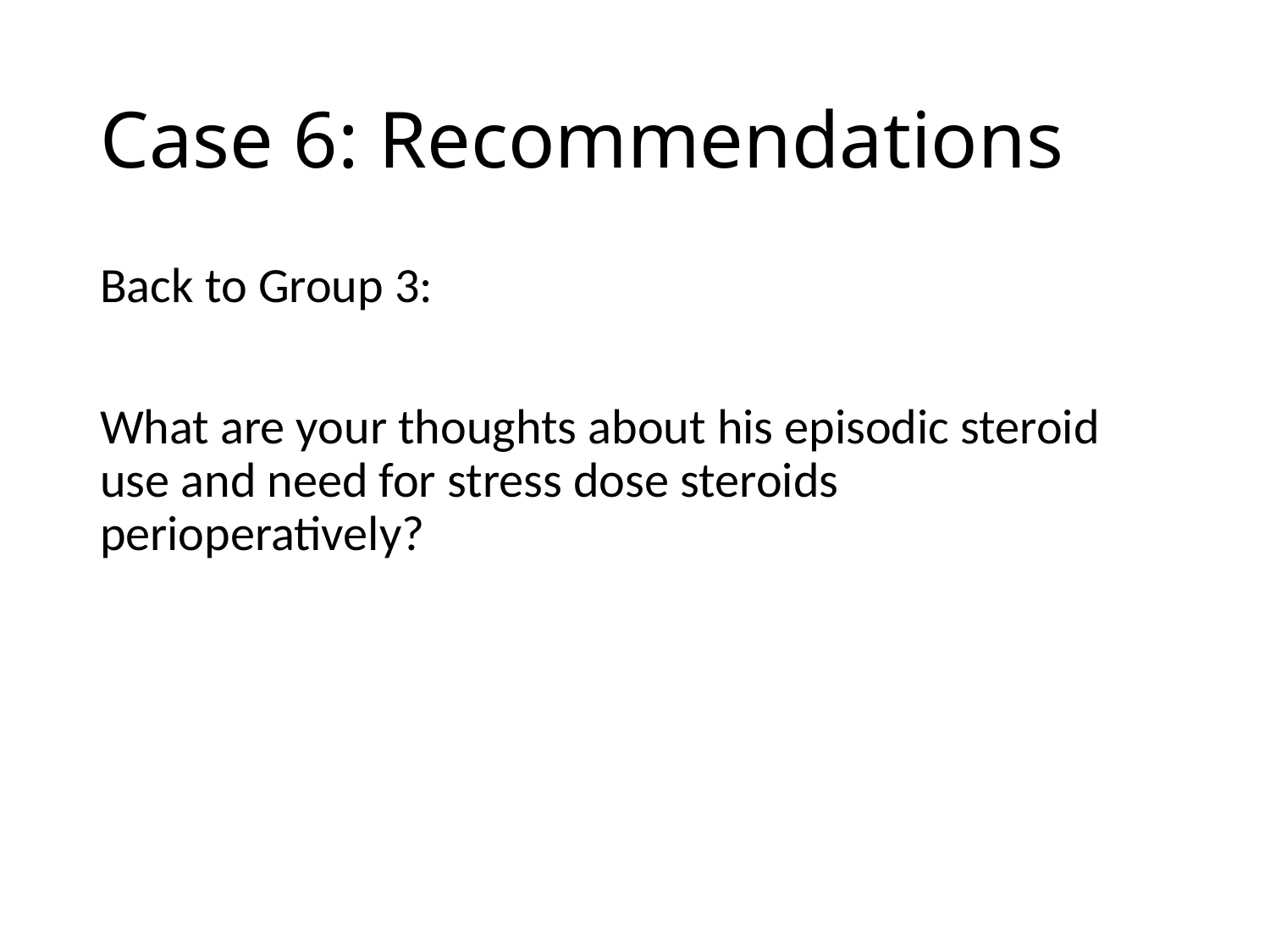

# Case 6: Recommendations
Back to Group 3:
What are your thoughts about his episodic steroid use and need for stress dose steroids perioperatively?

## Slide 84
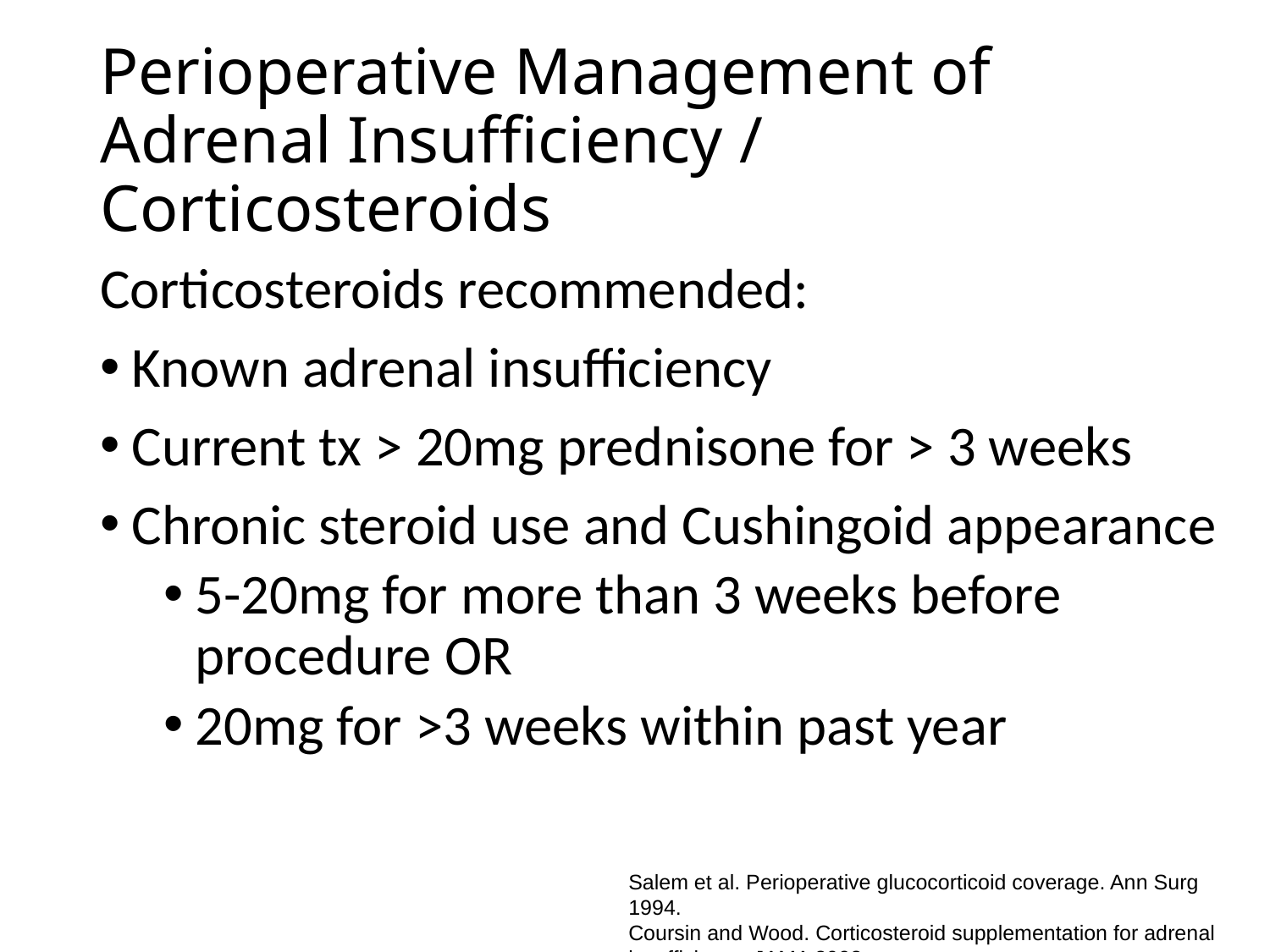

# Perioperative Management of Adrenal Insufficiency / Corticosteroids
Corticosteroids recommended:
Known adrenal insufficiency
Current tx > 20mg prednisone for > 3 weeks
Chronic steroid use and Cushingoid appearance
5-20mg for more than 3 weeks before procedure OR
20mg for >3 weeks within past year
Salem et al. Perioperative glucocorticoid coverage. Ann Surg 1994.
Coursin and Wood. Corticosteroid supplementation for adrenal insufficiency. JAMA 2002.

## Slide 85
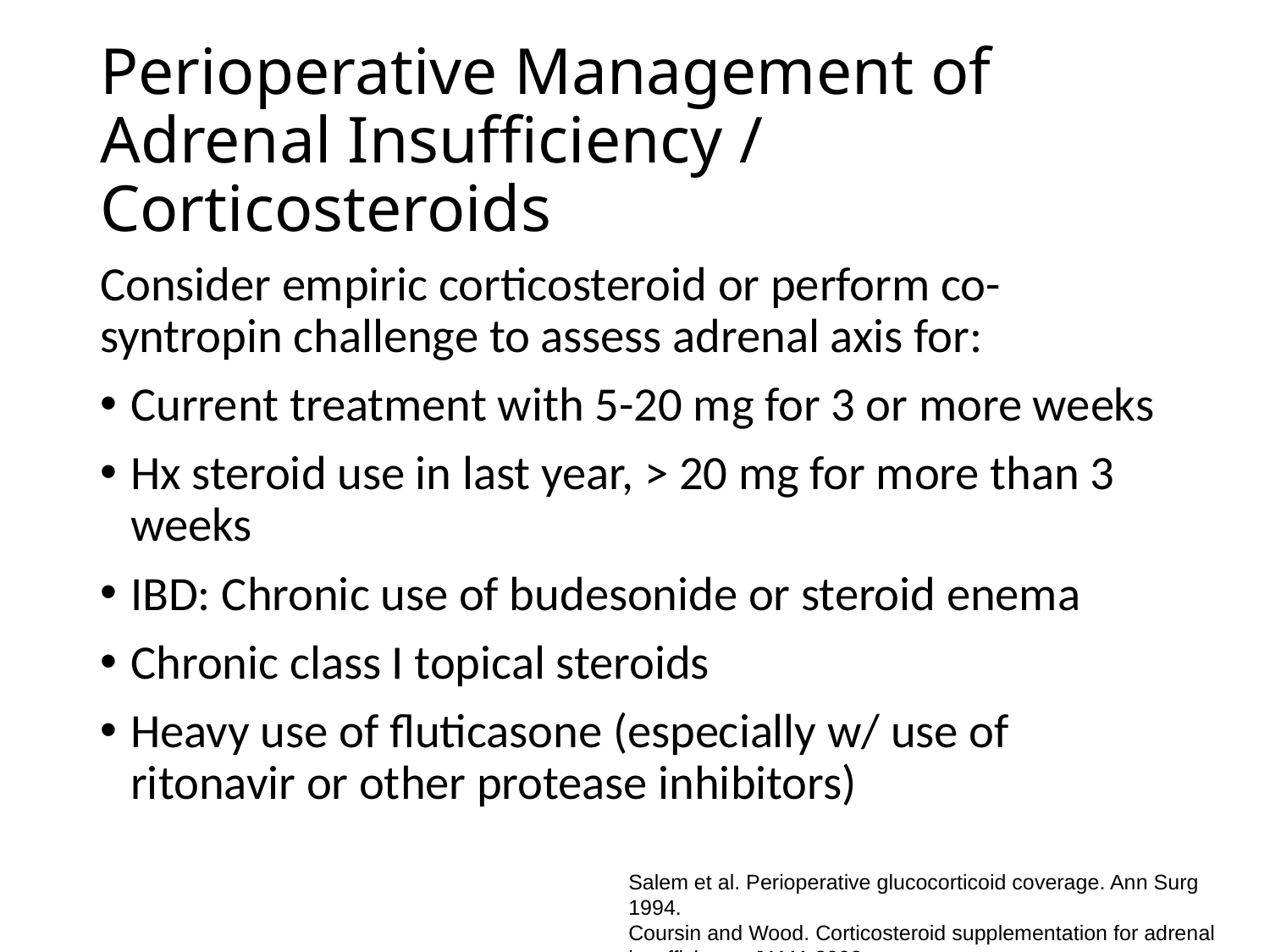

# Perioperative Management of Adrenal Insufficiency / Corticosteroids
Consider empiric corticosteroid or perform co-syntropin challenge to assess adrenal axis for:
Current treatment with 5-20 mg for 3 or more weeks
Hx steroid use in last year, > 20 mg for more than 3 weeks
IBD: Chronic use of budesonide or steroid enema
Chronic class I topical steroids
Heavy use of fluticasone (especially w/ use of ritonavir or other protease inhibitors)
Salem et al. Perioperative glucocorticoid coverage. Ann Surg 1994.
Coursin and Wood. Corticosteroid supplementation for adrenal insufficiency. JAMA 2002.

## Slide 86
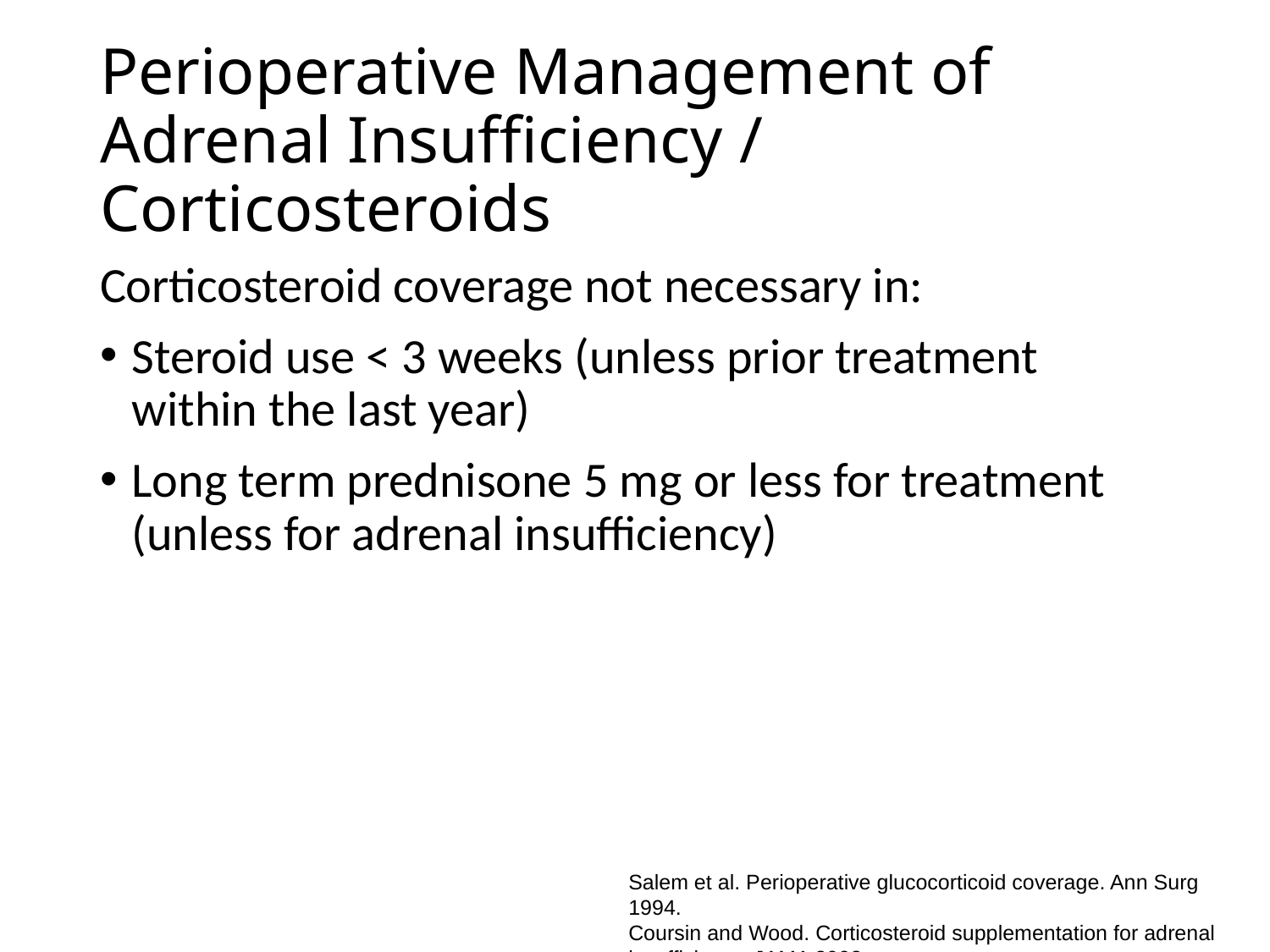

# Perioperative Management of Adrenal Insufficiency / Corticosteroids
Corticosteroid coverage not necessary in:
Steroid use < 3 weeks (unless prior treatment within the last year)
Long term prednisone 5 mg or less for treatment (unless for adrenal insufficiency)
Salem et al. Perioperative glucocorticoid coverage. Ann Surg 1994.
Coursin and Wood. Corticosteroid supplementation for adrenal insufficiency. JAMA 2002.

## Slide 87
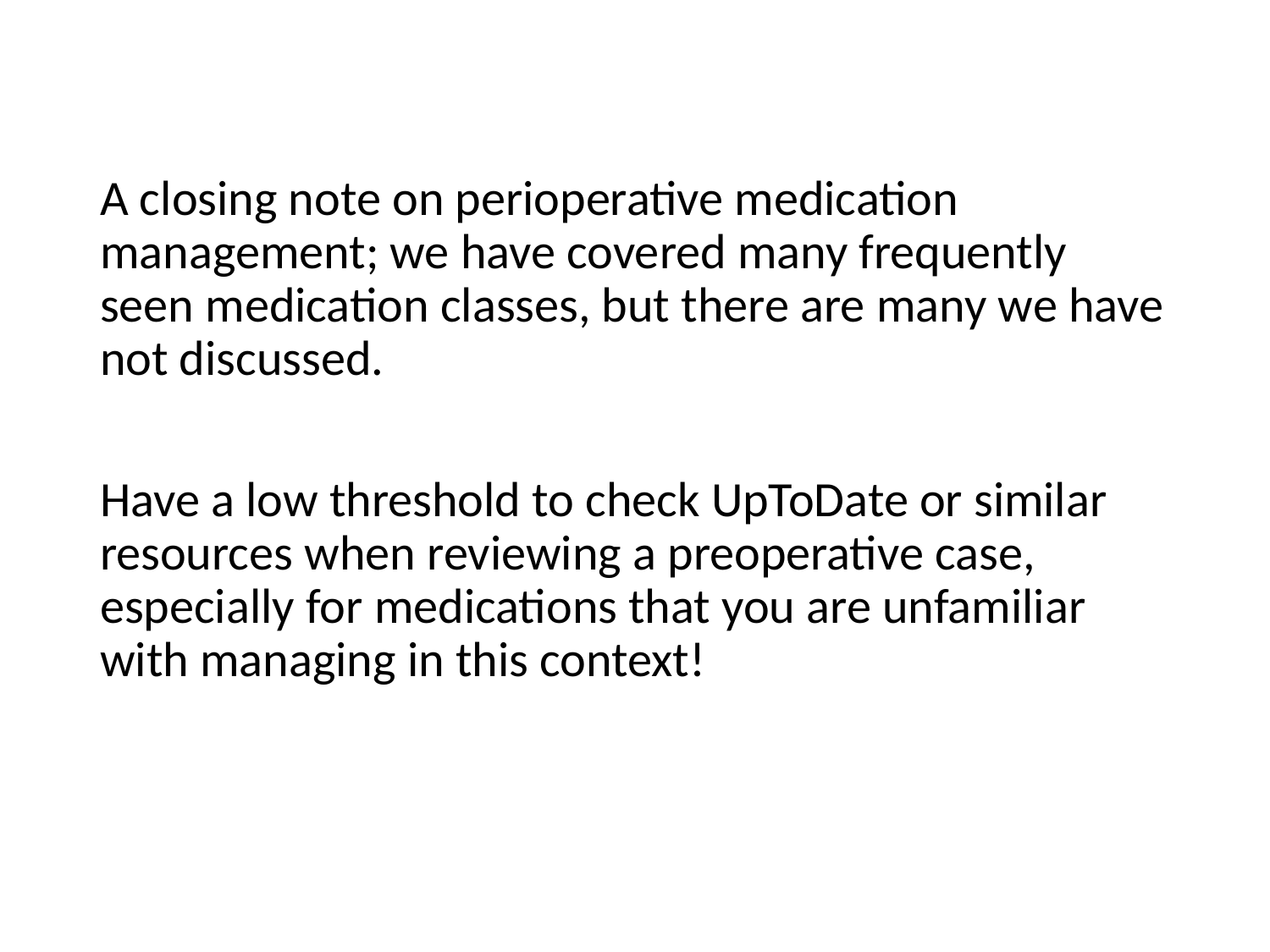

A closing note on perioperative medication management; we have covered many frequently seen medication classes, but there are many we have not discussed.
Have a low threshold to check UpToDate or similar resources when reviewing a preoperative case, especially for medications that you are unfamiliar with managing in this context!

## Slide 88
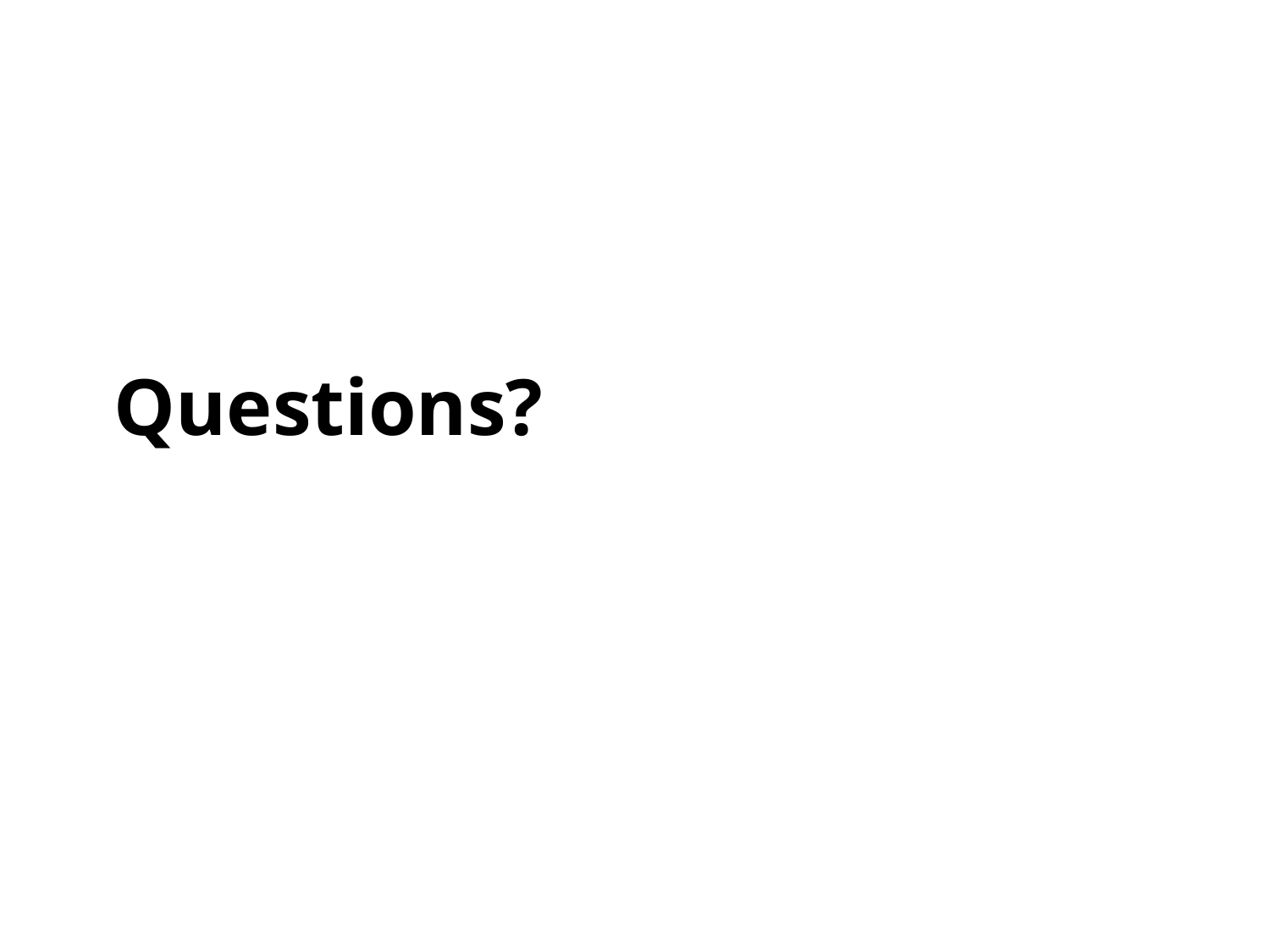

# Questions?

## Slide 89
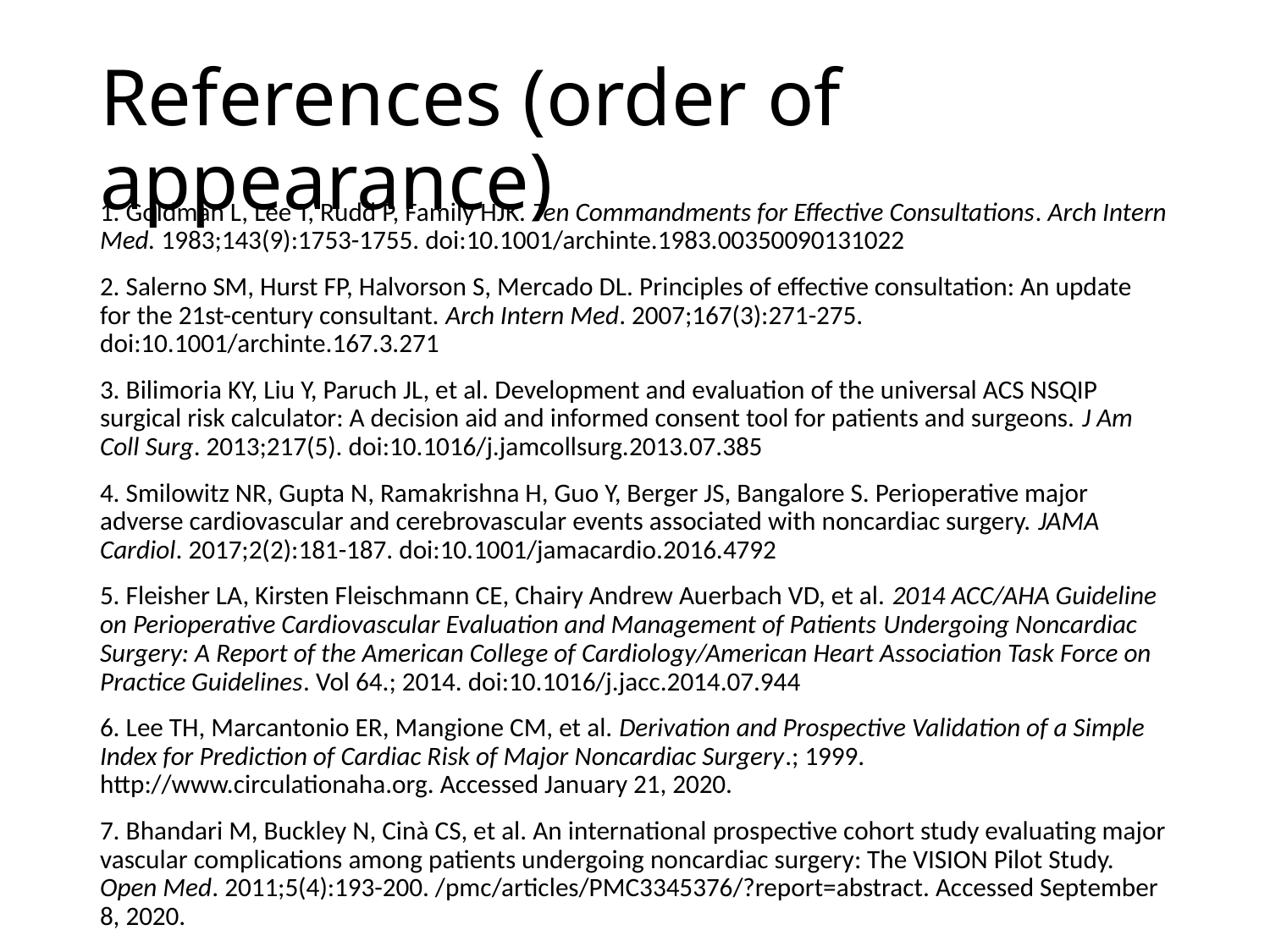

# References (order of appearance)
1. Goldman L, Lee T, Rudd P, Family HJK. Ten Commandments for Effective Consultations. Arch Intern Med. 1983;143(9):1753-1755. doi:10.1001/archinte.1983.00350090131022
2. Salerno SM, Hurst FP, Halvorson S, Mercado DL. Principles of effective consultation: An update for the 21st-century consultant. Arch Intern Med. 2007;167(3):271-275. doi:10.1001/archinte.167.3.271
3. Bilimoria KY, Liu Y, Paruch JL, et al. Development and evaluation of the universal ACS NSQIP surgical risk calculator: A decision aid and informed consent tool for patients and surgeons. J Am Coll Surg. 2013;217(5). doi:10.1016/j.jamcollsurg.2013.07.385
4. Smilowitz NR, Gupta N, Ramakrishna H, Guo Y, Berger JS, Bangalore S. Perioperative major adverse cardiovascular and cerebrovascular events associated with noncardiac surgery. JAMA Cardiol. 2017;2(2):181-187. doi:10.1001/jamacardio.2016.4792
5. Fleisher LA, Kirsten Fleischmann CE, Chairy Andrew Auerbach VD, et al. 2014 ACC/AHA Guideline on Perioperative Cardiovascular Evaluation and Management of Patients Undergoing Noncardiac Surgery: A Report of the American College of Cardiology/American Heart Association Task Force on Practice Guidelines. Vol 64.; 2014. doi:10.1016/j.jacc.2014.07.944
6. Lee TH, Marcantonio ER, Mangione CM, et al. Derivation and Prospective Validation of a Simple Index for Prediction of Cardiac Risk of Major Noncardiac Surgery.; 1999. http://www.circulationaha.org. Accessed January 21, 2020.
7. Bhandari M, Buckley N, Cinà CS, et al. An international prospective cohort study evaluating major vascular complications among patients undergoing noncardiac surgery: The VISION Pilot Study. Open Med. 2011;5(4):193-200. /pmc/articles/PMC3345376/?report=abstract. Accessed September 8, 2020.

## Slide 90
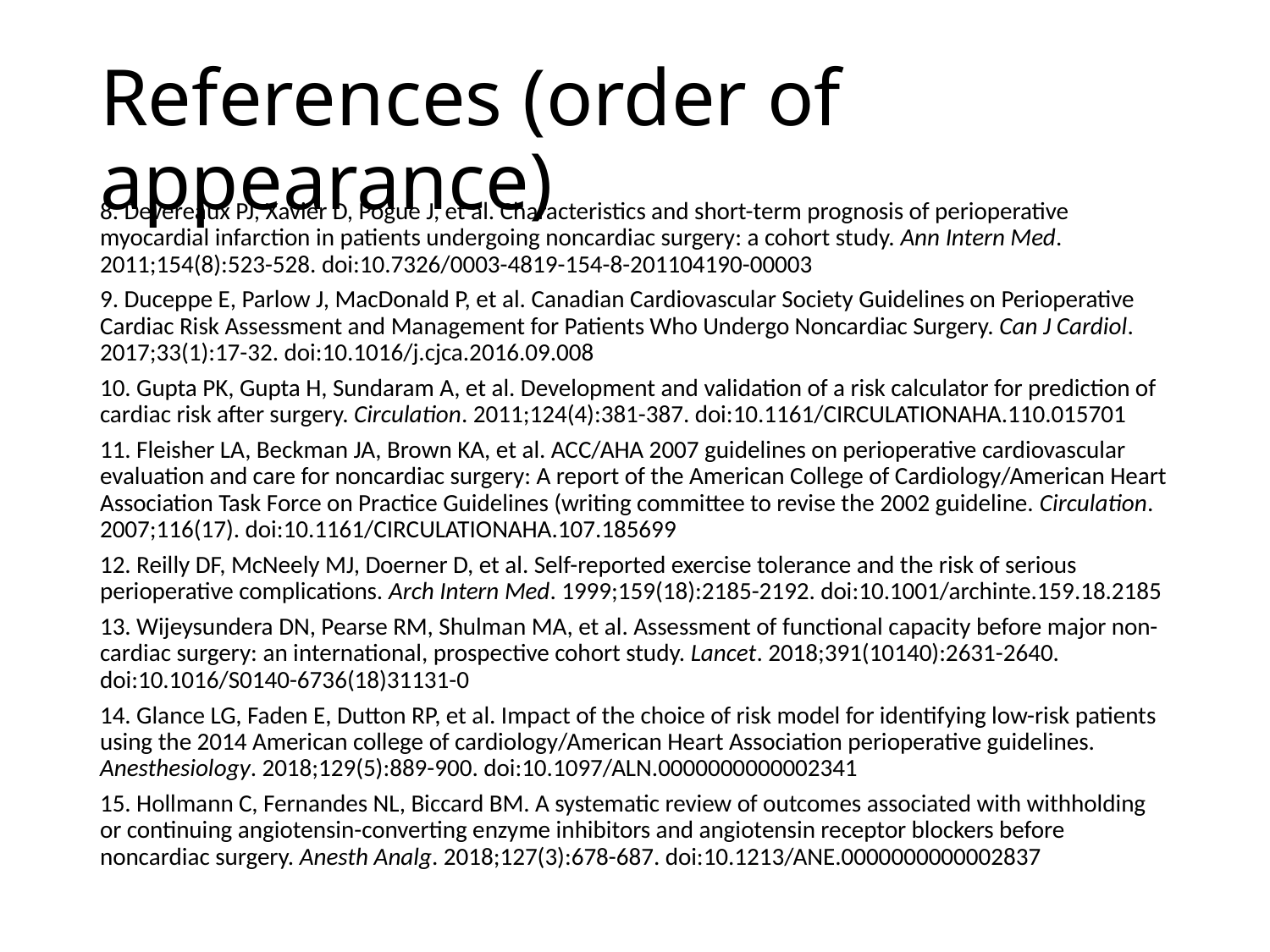

# References (order of appearance)
8. Devereaux PJ, Xavier D, Pogue J, et al. Characteristics and short-term prognosis of perioperative myocardial infarction in patients undergoing noncardiac surgery: a cohort study. Ann Intern Med. 2011;154(8):523-528. doi:10.7326/0003-4819-154-8-201104190-00003
9. Duceppe E, Parlow J, MacDonald P, et al. Canadian Cardiovascular Society Guidelines on Perioperative Cardiac Risk Assessment and Management for Patients Who Undergo Noncardiac Surgery. Can J Cardiol. 2017;33(1):17-32. doi:10.1016/j.cjca.2016.09.008
10. Gupta PK, Gupta H, Sundaram A, et al. Development and validation of a risk calculator for prediction of cardiac risk after surgery. Circulation. 2011;124(4):381-387. doi:10.1161/CIRCULATIONAHA.110.015701
11. Fleisher LA, Beckman JA, Brown KA, et al. ACC/AHA 2007 guidelines on perioperative cardiovascular evaluation and care for noncardiac surgery: A report of the American College of Cardiology/American Heart Association Task Force on Practice Guidelines (writing committee to revise the 2002 guideline. Circulation. 2007;116(17). doi:10.1161/CIRCULATIONAHA.107.185699
12. Reilly DF, McNeely MJ, Doerner D, et al. Self-reported exercise tolerance and the risk of serious perioperative complications. Arch Intern Med. 1999;159(18):2185-2192. doi:10.1001/archinte.159.18.2185
13. Wijeysundera DN, Pearse RM, Shulman MA, et al. Assessment of functional capacity before major non-cardiac surgery: an international, prospective cohort study. Lancet. 2018;391(10140):2631-2640. doi:10.1016/S0140-6736(18)31131-0
14. Glance LG, Faden E, Dutton RP, et al. Impact of the choice of risk model for identifying low-risk patients using the 2014 American college of cardiology/American Heart Association perioperative guidelines. Anesthesiology. 2018;129(5):889-900. doi:10.1097/ALN.0000000000002341
15. Hollmann C, Fernandes NL, Biccard BM. A systematic review of outcomes associated with withholding or continuing angiotensin-converting enzyme inhibitors and angiotensin receptor blockers before noncardiac surgery. Anesth Analg. 2018;127(3):678-687. doi:10.1213/ANE.0000000000002837

## Slide 91
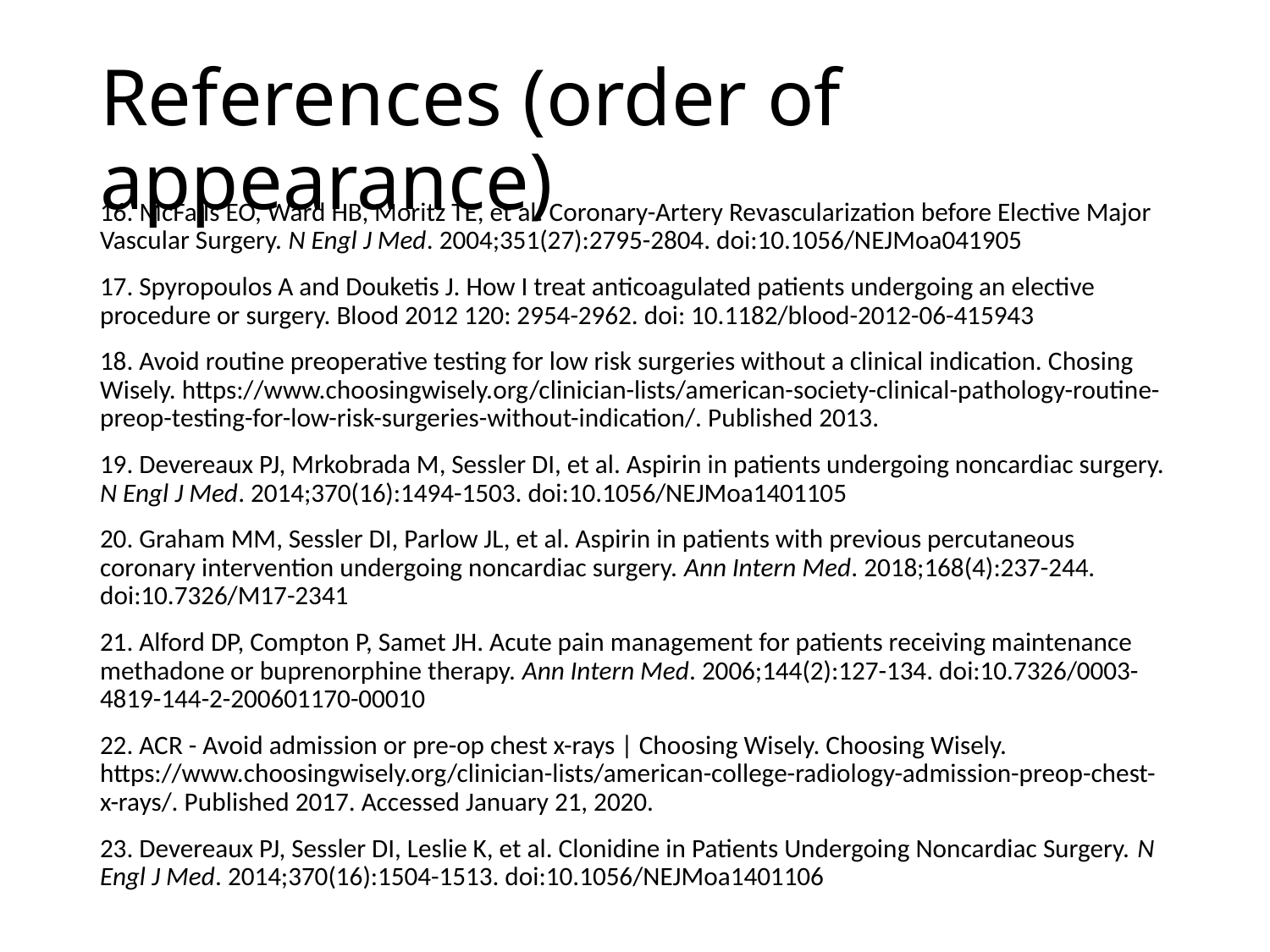

# References (order of appearance)
16. McFalls EO, Ward HB, Moritz TE, et al. Coronary-Artery Revascularization before Elective Major Vascular Surgery. N Engl J Med. 2004;351(27):2795-2804. doi:10.1056/NEJMoa041905
17. Spyropoulos A and Douketis J. How I treat anticoagulated patients undergoing an elective procedure or surgery. Blood 2012 120: 2954-2962. doi: 10.1182/blood-2012-06-415943
18. Avoid routine preoperative testing for low risk surgeries without a clinical indication. Chosing Wisely. https://www.choosingwisely.org/clinician-lists/american-society-clinical-pathology-routine-preop-testing-for-low-risk-surgeries-without-indication/. Published 2013.
19. Devereaux PJ, Mrkobrada M, Sessler DI, et al. Aspirin in patients undergoing noncardiac surgery. N Engl J Med. 2014;370(16):1494-1503. doi:10.1056/NEJMoa1401105
20. Graham MM, Sessler DI, Parlow JL, et al. Aspirin in patients with previous percutaneous coronary intervention undergoing noncardiac surgery. Ann Intern Med. 2018;168(4):237-244. doi:10.7326/M17-2341
21. Alford DP, Compton P, Samet JH. Acute pain management for patients receiving maintenance methadone or buprenorphine therapy. Ann Intern Med. 2006;144(2):127-134. doi:10.7326/0003-4819-144-2-200601170-00010
22. ACR - Avoid admission or pre-op chest x-rays | Choosing Wisely. Choosing Wisely. https://www.choosingwisely.org/clinician-lists/american-college-radiology-admission-preop-chest-x-rays/. Published 2017. Accessed January 21, 2020.
23. Devereaux PJ, Sessler DI, Leslie K, et al. Clonidine in Patients Undergoing Noncardiac Surgery. N Engl J Med. 2014;370(16):1504-1513. doi:10.1056/NEJMoa1401106

## Slide 92
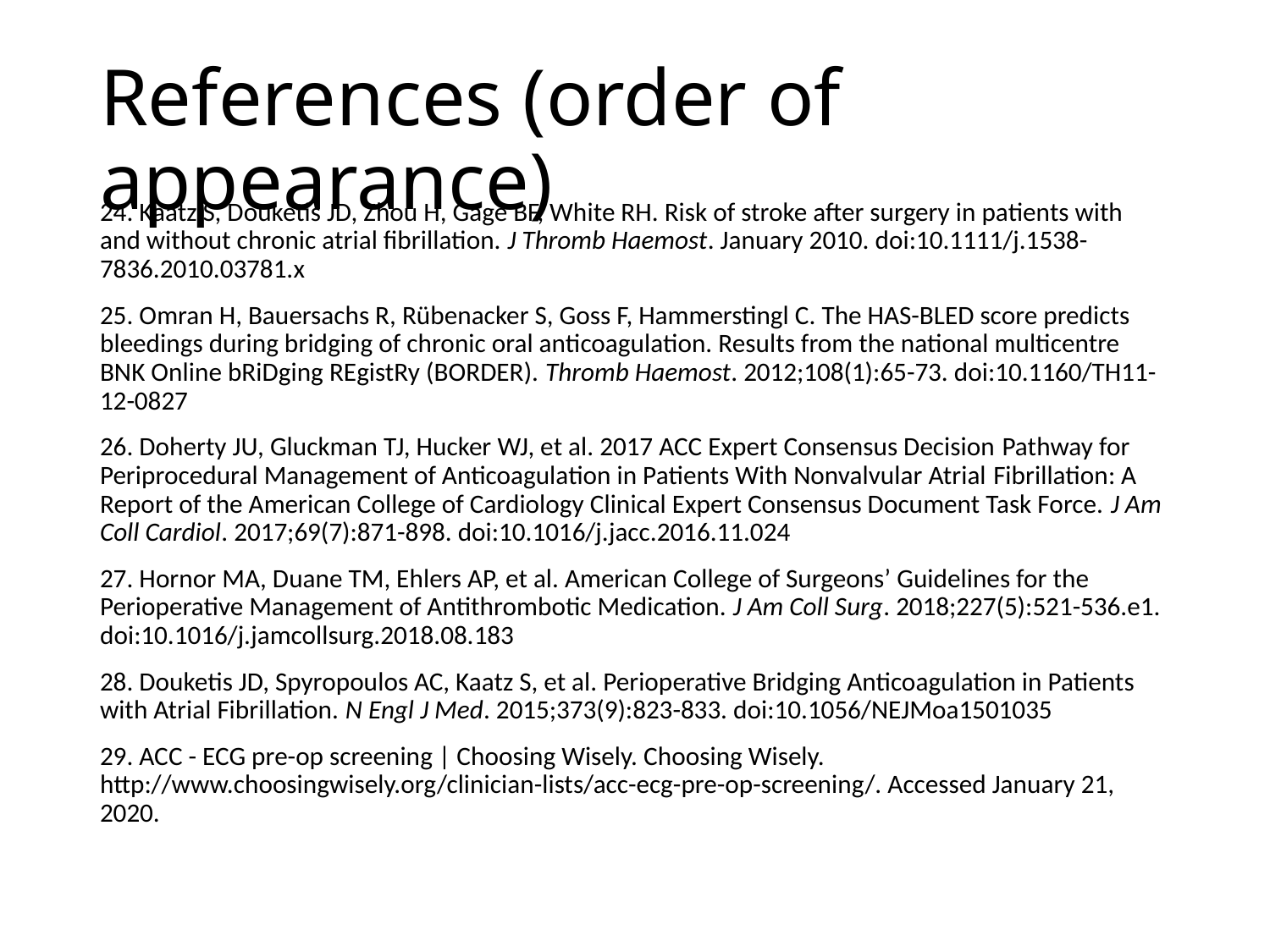

# References (order of appearance)
24. Kaatz S, Douketis JD, Zhou H, Gage BF, White RH. Risk of stroke after surgery in patients with and without chronic atrial fibrillation. J Thromb Haemost. January 2010. doi:10.1111/j.1538-7836.2010.03781.x
25. Omran H, Bauersachs R, Rübenacker S, Goss F, Hammerstingl C. The HAS-BLED score predicts bleedings during bridging of chronic oral anticoagulation. Results from the national multicentre BNK Online bRiDging REgistRy (BORDER). Thromb Haemost. 2012;108(1):65-73. doi:10.1160/TH11-12-0827
26. Doherty JU, Gluckman TJ, Hucker WJ, et al. 2017 ACC Expert Consensus Decision Pathway for Periprocedural Management of Anticoagulation in Patients With Nonvalvular Atrial Fibrillation: A Report of the American College of Cardiology Clinical Expert Consensus Document Task Force. J Am Coll Cardiol. 2017;69(7):871-898. doi:10.1016/j.jacc.2016.11.024
27. Hornor MA, Duane TM, Ehlers AP, et al. American College of Surgeons’ Guidelines for the Perioperative Management of Antithrombotic Medication. J Am Coll Surg. 2018;227(5):521-536.e1. doi:10.1016/j.jamcollsurg.2018.08.183
28. Douketis JD, Spyropoulos AC, Kaatz S, et al. Perioperative Bridging Anticoagulation in Patients with Atrial Fibrillation. N Engl J Med. 2015;373(9):823-833. doi:10.1056/NEJMoa1501035
29. ACC - ECG pre-op screening | Choosing Wisely. Choosing Wisely. http://www.choosingwisely.org/clinician-lists/acc-ecg-pre-op-screening/. Accessed January 21, 2020.

## Slide 93
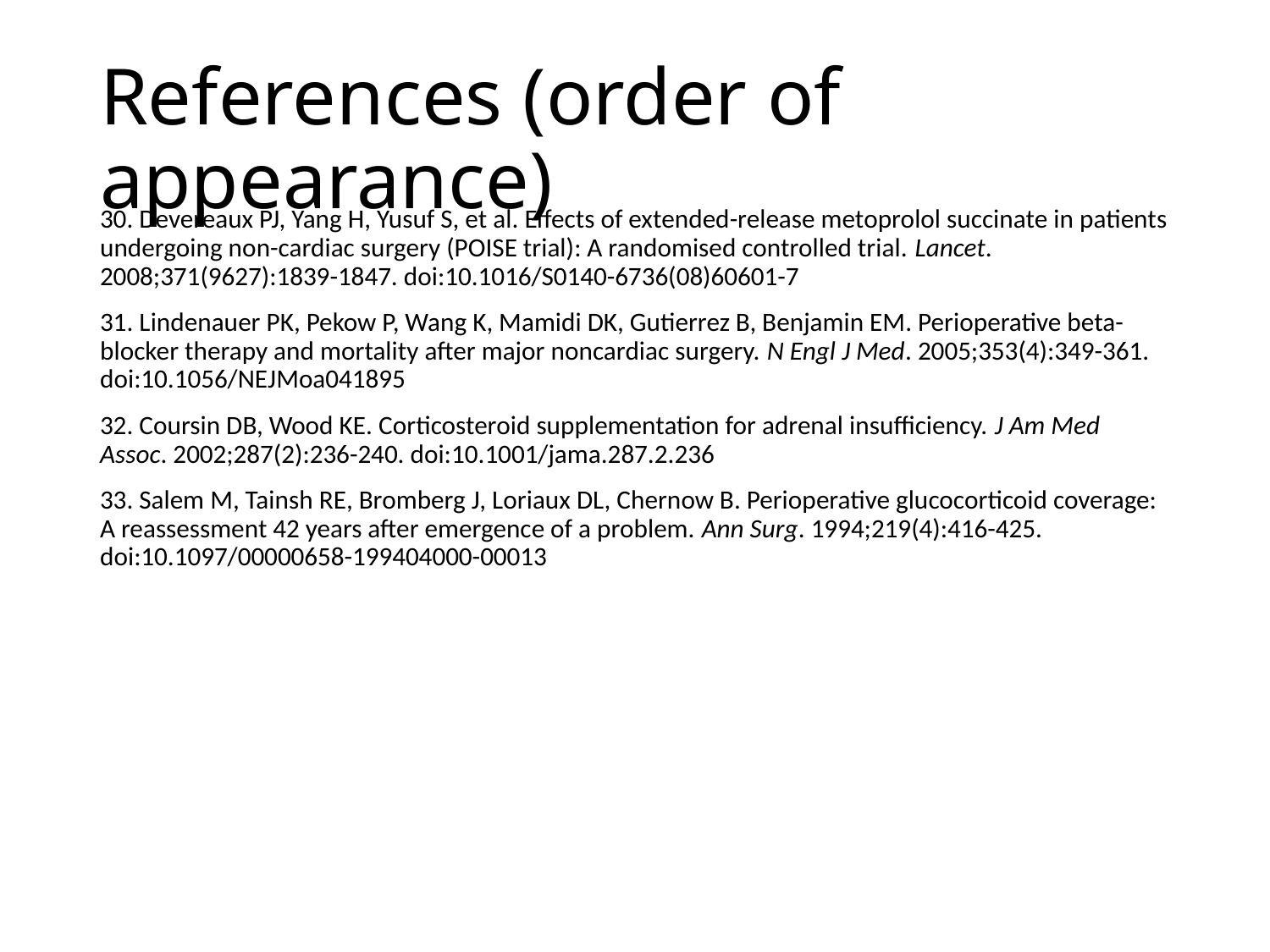

# References (order of appearance)
30. Devereaux PJ, Yang H, Yusuf S, et al. Effects of extended-release metoprolol succinate in patients undergoing non-cardiac surgery (POISE trial): A randomised controlled trial. Lancet. 2008;371(9627):1839-1847. doi:10.1016/S0140-6736(08)60601-7
31. Lindenauer PK, Pekow P, Wang K, Mamidi DK, Gutierrez B, Benjamin EM. Perioperative beta-blocker therapy and mortality after major noncardiac surgery. N Engl J Med. 2005;353(4):349-361. doi:10.1056/NEJMoa041895
32. Coursin DB, Wood KE. Corticosteroid supplementation for adrenal insufficiency. J Am Med Assoc. 2002;287(2):236-240. doi:10.1001/jama.287.2.236
33. Salem M, Tainsh RE, Bromberg J, Loriaux DL, Chernow B. Perioperative glucocorticoid coverage: A reassessment 42 years after emergence of a problem. Ann Surg. 1994;219(4):416-425. doi:10.1097/00000658-199404000-00013
