# Supplementary material for: Essential Principles of Preoperative Assessment in Internal Medicine: A Case-Based Teaching Session
Source: MedEdPORTAL. 2021 Aug 5;17:11178. doi: 10.15766/mep_2374-8265.11178 (PMC8339074; doi:10.15766/mep_2374-8265.11178)
Supplement: Supplementary file 1 — Preop Assessment and Management Slideshow.pptxCases 1-3.docxCases 4-6.docxPre- and Postassessment.docx [file mep_2374-8265.11178-s001.zip › B. Cases 1-3.docx]

**Case 1**

**Consult question**: “preoperative clearance”

**Consulting service**: orthopedics

**HPI**: 62-year-old man with known CAD (based on stress test ~5 years ago for dyspnea), HFpEF, and CKD presently hospitalized for right tibia fracture after syncope and fall. He had some nausea and vomiting yesterday after eating out at Yadavalli Café Fine Italian Food, and still felt unwell this morning, and as such did not eat or drink anything since yesterday at lunch. He took his medications as usual, was walking to the grocery store slowly when he became acutely lightheaded going up a small flight of stairs in the park, syncopized, and awoke 3 stairs down on concrete with a broken right leg. He is unsure about head strike, but denies any sore spots on his head.

At baseline, he ambulates with a cane without particular limitation. He specifically can walk on flat surfaces for 30 minutes without stopping, and can go up 2 flights of stairs before he has to take a break due to being winded. He denies any chest pain with exertion. His CHF has been well controlled, last admission 2 years ago; he reports being at his baseline weight, denies LE edema, orthopnea, or PND. He denies snoring. He has had one prior operation before in his 30’s, and denies any issues with anesthesia or post-operative care at that point.

**PMH**: CAD, HFpEF, CKD, HTN, BPH, prior appendectomy

**Meds**:

Furosemide 20mg qday

Lisinopril 40mg qday

Aspirin 81mg qday

Atorvastatin 20mg QHS

Tamsulosin 0.4mg QHS

Acetaminophen 500mg prn

**Allergies**: NKDA

**SH/FH**: prior smoker (40 pack year quit 5 years ago), social EtOH, no illicits; lives with wife, 2 children both in Boston; unemployed

**ROS**: as per HPI

**Exam**: afebrile, HR 55 (baseline), BP 140/75, sat 97% RA; 180 cm, 90kg

*General*: NAD, sitting in bed

*Cardiovascular*: brady but regular, no MRG, JVP 7cm

*Pulmonary*: clearing crackles @ L base

*Abdomen*: soft, NT/ND, no organomegaly

*Extremities*: tr pitting LE edema bilaterally up shin; slight deformity in R shin with swelling and ecchymosis midway up leg

Intact pulses and sensation in R distal LE

*Neurological*: A&Ox3, no pronator drift, grossly intact strength in all limbs sparing R LE

**Labs (notable only)**:

Hgb 11 (baseline), plt 150

Na 142, K 4.8, BUN 35, Cr 2.3 (baseline 1.8-2.1)

UA without pyuria

**Studies**:

EKG unchanged from prior, inf q waves, TWI across precordium, sinus rhythm @ 70bpm

CXR with mild cardiomegaly

Leg films with R non-displaced tibial fracture

**Planned operation**: CPT 27758, open reduction and internal fixation of tibial shaft fracture

**Case 2**

**Consult question**: “approval for OR”

**Consulting service**: ENT

**HPI**: 51-year-old man with HFrEF, diabetes, depression, and a recently diagnosed nasal carcinoma hospitalized prior to a planned excision of his nasal mass. He has progressive sinus symptoms over 6 months culminating in a difficult to stop nosebleed, and ENT evaluation discovered a friable 2cm mass in his right maxillary sinus, biopsy consistent with cancer.

At baseline, he ambulates without particular limitation. He specifically can walk on flat surfaces for 30 minutes without stopping, and can go up 2 flights of stairs before he has to take a break due to being winded. He denies any chest pain with exertion. He reports that over the last 3 weeks since his diagnosis he has been feeling much more dyspneic than baseline, however, with symptoms with minimal exertion. Over the last week has had orthopnea as well as occasional PND. He notes worsened LE edema from baseline. On questioning, his medication adherence has suffered lately due to new diagnosis – he has felt more depressed and has been skipping doses frequently. He has no prior operations, no family members with anesthesia reactions.

**PMH**: HFrEF (for 5 years, EF 30%, non-ischemic on cath, potentially cocaine related), depression, diabetes (10 years, diet controlled until last year), cocaine use disorder in remission, and nasal carcinoma

**Meds**:

Torsemide 40mg qday

Lisinopril 10mg qday

Metoprolol 100mg qday

Sertraline 100mg qday

Lorazepam 1mg TID prn (uses daily TID)

Metformin 1gm BID

Glipizide 5mg before dinner (largest meal)

Saline nasal sprays, BID-TID

**Allergies**: PCN (childhood, unknown)

**SH/FH**: never smoker, no EtOH, prior heavy cocaine use but sober x 4 years; works as bartender; in stable relationship with female partner

**ROS**: as per HPI; no weight loss recently, no nosebleeds since 3 weeks ago

**Exam**: afebrile, HR 90, BP 170/85, sat 91% RA; 190 cm, 100kg (dry weight 92kg)

*General*: mildly obese, sitting in bed, appears mildly dyspneic at rest

*Cardiovascular*: RRR, 2/6 HSM @ LLSB radiating to axillae, JVP 12cm @ 45 degrees

*Pulmonary*: bilateral crackles ½ up back

*Abdomen*: soft, NT, slight distention with possible fluid wave, no organomegaly

*Extremities*: 2+ pitting LE edema up to knees, trace edema up thighs

*Neurological*: A&Ox3, grossly intact strength in all limbs

*Psychiatry*: flat affect, denies SI/HI

**Labs (notable only)**:

Hgb 10 with MCV 79, plt 210

Na 138, K 4.1, BUN 35, Cr 1.6 (baseline 1.2)

**Studies**:

EKG NSR @ 80bpm with 1^st^ degree AV block, TWI diffusely across precordium (stable from prior)

MRI of head with 3 cm mass in right maxillary sinus with invasion into underlying cartilage, no lymphadenopathy

**Planned operation**: CPT 21034 - Excision of malignant tumor of maxilla or zygoma

**Case 3**

**Consult question**: “preop please”

**Consulting service**: orthopedics

**HPI**: 70-year-old man with prior CVA without residual weakness, diabetes on insulin, HTN, and severe OA presently hospitalized for an elective right knee replacement. He has had progressive worsening of bilateral knee pain over the last 2 years, with mild improvements after PT and steroid injections, but has now reached a point of severe limitation particularly on his right warranting joint replacement.

At baseline he walks with a rolling walker, slowly. He cannot do a flight of stairs without stopping due to pain in his right knee; he can walk for about 1 city block before stopping on flat surfaces. He denies any chest pain or dyspnea with exertion. He had a prior CEA which was complicated by delirium post-operatively.

**PMH**: CVA (7 years ago), DM (longstanding), severe OA in knees bilaterally (as above), HTN, carotid stenosis s/p R CEA 6 years prior

**Meds**:

Clopidogrel 75mg qday

Losartan 25mg qday

Amlodipine 5mg qday

Metformin 500mg BID

Insulin NPH 70/30 20 units BID before meals

Oxycodone 10mg TID prn (confirmed in online opioid monitoring program)

Senna 2 tabs BID prn

**Allergies**: ACE-I, cough; Keflex, severe rash

**SH/FH**: present smoker (2-3 cigarettes/day), 50 pack-year history; social EtOH, heavy use in 30’s and 40’s; no illicits; prior employment as factory laborer; lives on first floor with wife, no family in town

**ROS**: as per HPI

**Exam**: afebrile, HR 65, BP 120/70, sat 91% RA; 170 cm, 80kg

*General*: NAD, walking slowly around room

*Cardiovascular*: RRR, no MRG, JVP 6cm

*Pulmonary*: clear to auscultation bilaterally

*Abdomen*: soft, NT, ND

*Extremities*: no LE edema, slight R knee effusion

*Neurological*: A&Ox3, grossly intact strength in all limbs

**Labs (notable only)**:

Hgb 13, plt 180

Na 136, K 4.8, BUN 20, Cr 1.2 (baseline 1.1)

**Studies**:

EKG NSR @ 60bpm with lateral q waves, LVH by AVL criteria, all stable from prior

CXR with clear lungs, tortuous aorta, mild cardiomegaly

**Planned operation**: CPT 27447 - Arthroplasty, knee, condyle and plateau; medial AND lateral compartments with or without patella resurfacing (total knee arthroplasty)
