# Supplementary material for: Essential Principles of Preoperative Assessment in Internal Medicine: A Case-Based Teaching Session
Source: MedEdPORTAL. 2021 Aug 5;17:11178. doi: 10.15766/mep_2374-8265.11178 (PMC8339074; doi:10.15766/mep_2374-8265.11178)
Supplement: Supplementary file 1 — Preop Assessment and Management Slideshow.pptxCases 1-3.docxCases 4-6.docxPre- and Postassessment.docx [file mep_2374-8265.11178-s001.zip › C. Cases 4-6.docx]

**Case 4**

**Consult question**: “preoperative clearance”

**HPI**: 70 M hx of HTN, DM, HFpEF, tobacco use, opioid abuse disorder on buprenorphine, and osteoarthritis who presents to clinic for evaluation prior to undergoing right total knee arthroplasty. He has been having pain in the right knee for the past 5 years which has progressed and is now very substantial. A few years ago he was exercising regularly by hiking and riding a bicycle but his knee pain has become too severe. He is still able to walk unassisted and can walk 6 blocks to the grocery store and back and does not experience any shortness of breath or chest pain.

He has been a smoker for many years and you have made valiant efforts at smoking cessation but you were unsuccessful, however he did quit 2 months ago in anticipation of this surgery. He denies any recent orthopnea, PND, or lower extremity edema. He denies hematuria, urgency, dysuria, or hesitancy.

He also mentions that his surgeon wanted him to get a urinalysis checked while at this visit, “because it is a routine thing they do before surgery.”

**PMH**:

HFpEF: diagnosed 20 years ago after having been out of medical care for several years when he presented with mild SOB and LE edema. He had been on no medications up until that point and had resolution of symptoms upon control of his HTN and addition of diuretic. An exercise echo around that time was without signs of ischemia. His most recent TTE is from 2 years ago and showed an EF of 65%, normal wall systolic motion and grade I diastolic dysfunction.

HTN: diagnosed 20 years ago, no issues since.

DM: diagnosed 20 years ago, well controlled with metformin since.

Opioid abuse disorder: history of heroin intranasal and injection in the 1970’s. Quit for 20 years but then relapsed with intranasal use again 15 years ago. Has been on buprenorphine now for 10 years with success in sobriety; monitoring urine toxicology all negative (except buprenorphine)

**Meds:**

Metformin 1000 mg bid

Lisinopril 20 mg qd

Metoprolol succinate 25 mg qd

Furosemide 20 mg qd

Aspirin 81 mg qd

Chondroitin 1200 mg qd

Buprenorphine / Naloxone 8mg/2mg 1 tab bid

Acetaminophen 650 prn

**Allergies**: Shellfish, Peanuts

**SH/FH**: former (recent) smoker. Prior excesses alcohol, LSD, and marijuana use (quit 30 years ago). Retired road manager for a successful late 1970’s era rock band. Lives with wife. Has 3 adult children.

**ROS**: knee pain and otherwise negative.

**Exam**: 98.4F, HR 72, BP 118/67, 97% RA,

Weight 70 kg / height 5’11’’

*General*: Well appearing thin man

*Cardiovascular*: JVP 7 cm, S1S2 RRR, no S4

*Pulmonary*: CTAB

*Abdomen*: Soft NT/ND no rebound or guarding

*Extremities*: No LE edema; R knee moderate effusion, tenderness across joint line

*Neurological*: Antalgic gait

**Labs (3 months ago)**

Hgb 13.5

BUN 17 / Cr 0.85

HgbA1c 7.2

PSA 0.3

**Studies:**

R knee xray: tri-compartmental degenerative changes

EKG: NSR at 78, normal axis, LVH, anterior precordial Jpoint elevation, no Qwaves or TWI

**Planned procedure:** CPT 27447 - Right Total Knee Arthroplasty

**Case 5**

**Consult question**: “appropriateness for elective surgery”

**HPI:** 70 year old woman hx of HTN, DM, HFrEF (most recent EF 49%), and atrial fibrillation who presents for evaluation prior to a planned R total hip arthroplasty.

She has been having pain in the right hip for the past 5 years which has progressed and is now very substantial. A few years ago she was exercising regularly by hiking and riding a bicycle but her hip pain has become too severe. She is still able to walk unassisted and can walk 6 blocks to the grocery store and back and does not experience any shortness of breath or chest pain.

She has been in good health and has had routine medical follow up and good medication compliance for 20+ years. She denies any recent SOB, orthopnea, PND, or lower extremity edema. She is a lifelong non-smoker and denies recent cough or wheezing.

She also mentions that her surgeon wanted him to get a chest Xray checked while at this visit, “because it is a routine thing they do before surgery.”

**PMHx**:

HFrEF: A cath at diagnosis showed clean coronaries. Her most recent TTE is from 1 year ago and showed an EF of 49%, normal wall systolic motion and normal valves.

Afib: diagnosed 35 years ago at time of CHF presentation. She has been on warfarin for years with good follow up in the anticoagulation clinic and INR usually at goal between 2-3.

HTN: diagnosed 35 years ago, no issues since.

DM: diagnosed 20 years ago, well controlled

with metformin since.

**Meds**:

Metformin 1000 mg bid

Lisinopril 20 mg qd

Clonidine 0.2mg TID

Metoprolol succinate 25 mg qd

Furosemide 20 mg qd

Warfarin 3 mg qhs

Chondroitin 1200 mg qd

Acetaminophen 650 prn

**Allergies**: Amoxicillin

**SH/FH**: Life long never smoker and no drug use. Occasional moderate social alcohol. She is a semi-retired nun and has a number of siblings and nieces and nephews in the area. She is very active within the church and the community.

**ROS**: R hip pain. Typically with long periods of sitting or long walks and otherwise negative.

**Exam**: 98.4F, HR 72, BP 118/67, 97% RA,

Weight 60 130 lbs / height 5’7’’

*General:* Well appearing woman.

*Cardiovascular:* JVP 7 cm, S1S2 irregularly irregular no murmurs or gallops.

*Pulmonary:* CTAB

*Abdomen:* Soft NT/ND no rebound or guarding

*Extremities:* No LE edema.

R hip pain with internal rotation.

*Neurological*: Antalgic gait

**Labs**

Hb 13.5

BUN 17 / Cr 0.85

HbA1c 7.2

25-hydroxy Vitamin D: 37

R hip xray: degenerative changes of the acetabulum and femoral head with a small bone spur noted.

EKG: Afib at 72 bpm

**Planned operation:** CPT 27130 - Right Total Hip Arthroplasty

**Case 6**

**Consult question:** “preoperative clearance”

**HPI**: 65 M hx of HTN, HFpEF, IDDM, TIA, COPD who presents to clinic for evaluation prior to undergoing a R total knee arthroplasty.

He has been having pain in the right knee for the past 5 years which has progressed and is now very substantial. A few years ago he was exercising regularly by hiking and riding a bicycle but his knee pain has become too severe. He is still able to walk unassisted and can walk 6 blocks to the grocery store and back and does not experience any shortness of breath or chest pain.

He has been a smoker for many years, and while he has reduced his smoking, has yet to successfully stop. He denies any recent orthopnea, PND, or lower extremity edema.

He also mentions that his surgeon wanted him to get an EKG checked while at this visit and also something about starting a type of blocker medication.

**PMHx**:

HFpEF: dx 20 years ago after having been out of medical care for several years when he presented with mild SOB and LE edema and difficulty with speech. He had been on no medications up until that point and had resolution of symptoms upon control of his HTN and addition of diuretic. An exercise echo around that time was without signs of ischemia. His most recent TTE is from 2 years ago and showed an EF of 65%, normal wall systolic motion and grade I diastolic dysfunction.

TIA: p/w transient aphasia and right arm weakness which resolved after 30 minutes. MRI was normal. Started on aspirin and statin at that time.

HTN: diagnosed 20 years ago, no issues since.

DM: diagnosed 20 years ago, was initially on metformin and glipizide, but was initiated on insulin ~ 5 years ago.

COPD: diagnosed on PFT’s 15 years ago. Generally pretty mild/inactive but did have one hospital admission which was 4 months ago when he was admitted to the ICU (but was not intubated) and required a 3-week long taper of prednisone afterwards (60->20 mg then stopped).

**Meds**:

Metformin 1000 mg bid

Atorvastatin 80mg QHS

Insulin glargine 20 units qhs

Lisinopril 20 mg qd

Furosemide 20 mg qd

Aspirin 81 mg qd

Tiotropium 2 puffs qd

Albuterol MDI PRN

Chondroitin 1200 mg qd

**Allergies**: Shellfish, Peanuts

**SH/FH**: active smoker, 1 ppd. No EtOH or illicits presently. Employed as CPA. Lives with wife. Has 2 adult children.

**ROS**: knee pain and otherwise negative.

**Exam**: 98.4F, HR 72, BP 118/67, 97% RA,

Weight 70 kg / height 5’11’’

*General*: Well appearing thin man

*Cardiovascular*: JVP 7 cm, S1S2 RRR, no S4

*Pulmonary*: CTAB

*Abdomen*: Soft NT/ND no rebound or guarding

*Extremities*: No LE edema; R knee moderate effusion, tenderness across joint line

*Neurological*: Antalgic gait

**Labs**

Hgb 13.5

BUN 17 / Cr 0.85

HgbA1c 7.2

**Studies:**

R knee xray: tri-compartmental degenerative changes

EKG: NSR at 78, normal axis, LVH, anterior precordial J point elevation, no Qwaves or TWI

**Planned procedure:** CPT 24447 - Right Total Knee Arthroplasty
