# Supplementary material for: Essential Principles of Preoperative Assessment in Internal Medicine: A Case-Based Teaching Session
Source: MedEdPORTAL. 2021 Aug 5;17:11178. doi: 10.15766/mep_2374-8265.11178 (PMC8339074; doi:10.15766/mep_2374-8265.11178)
Supplement: Supplementary file 1 — Preop Assessment and Management Slideshow.pptxCases 1-3.docxCases 4-6.docxPre- and Postassessment.docx [file mep_2374-8265.11178-s001.zip › D. Pre- and Postassessment.docx]

**Pre-Teaching Survey**

1. **How would you describe your ability to assess a patient prior to an operation and provide medical advice to a surgical team on appropriate preoperative planning?**

1 – none 2 – minimal 3 – moderate 4 – robust 5 – excellent

1. **Have you done any rotations featuring preoperative assessments thus far? (inpatient or outpatient, can include subspecialty rotations)**

Yes No

1. **List the names of up to three tools used to calculate perioperative cardiovascular risk:**

*RCRI Score, Gupta AKA MICA Calculator, NSQIP Surgical Risk Calculator [remove before printing]*

1. **Circle any medications below that are typically held before operations.**

Lisinopril Clonidine Clonazepam Metformin HCTZ

Furosemide Sitagliptin (Januvia) Metoprolol

1. **Which patients warrant an enoxaparin bridge to warfarin post-operatively (circle all that apply)?**

Recurrent PE, 7 months prior A-fib, CHADS2Vasc 2 A-fib, CHADS2Vasc 5

1. **Per guidelines, which patients could potentially have initiation of beta-blockers prior to operation? Assume all are NOT on beta-blockade prior and lack any contraindications – circle all that apply.**

Emergent AAA repair, known CAD+HFrEF IDDM undergoing hip replacement in 2 weeks

CAD+CKD+prior CVA, undergoing knee replacement in 4 weeks Emergent AAA repair, known CKD+CVA

1. **Circle any conditions that should postpone an elective operation to resect a gastric cancer:**

Pneumonia decompensated CHF hemoglobin of 8 atrial fibrillation, HR 150

**Post-Teaching Survey**

1. **List the names of up to three tools used to assess perioperative cardiovascular risk:**

*RCRI Score, Gupta AKA MICA Calculator, NSQIP Surgical Risk Calculator [remove before printing]*

1. **Circle any medications below that are typically held before operations.**

Lisinopril Clonidine Clonazepam Metformin HCTZ

Furosemide Sitagliptin (Januvia) Metoprolol

1. **Which patients warrant an enoxaparin bridge to warfarin post-operatively (circle all that apply)?**

Recurrent PE, 7 months prior A-fib, CHADS2Vasc 2 A-fib, CHADS2Vasc 5

1. **Per guidelines, which patients could potentially have initiation of beta-blockers prior to operation? Assume all are NOT on beta-blockade prior and lack any contraindications – circle all that apply.**

Emergent AAA repair, known CAD+HFrEF IDDM undergoing hip replacement in 2 weeks

CAD+CKD+prior CVA, undergoing knee replacement in 4 weeks Emergent AAA repair, known CKD+CVA

1. **Circle any conditions that should postpone an elective operation to resect a gastric cancer:**

Pneumonia decompensated CHF hemoglobin of 8 atrial fibrillation, HR 150

1. **How would you rate this session in terms of learning new material about perioperative medicine?**

1 – unhelpful, I learned nothing

2 – ok, I learned a few new things

3 – decent, I learned a fair amount

4 – great, it was mostly new material

5 – excellent, mostly new material and it makes sense to me

1. **How would you rate this session in terms of using a mixed lecture and case-based approach instead of a purely lecture-based approach?**

1 – much worse 2 – somewhat worse 3 – neutral 4 – better 5 – much better

1. **Please give us any recommendations for improvement; the more specific, the more useful!:**
