# Supplementary material for: Essential Principles of Preoperative Assessment in Internal Medicine: A Case-Based Teaching Session
Source: MedEdPORTAL. 2021 Aug 5;17:11178. doi: 10.15766/mep_2374-8265.11178 (PMC8339074; doi:10.15766/mep_2374-8265.11178)
Supplement: Supplementary file 1 — Preop Assessment and Management Slideshow.pptxCases 1-3.docxCases 4-6.docxPre- and Postassessment.docx [file mep_2374-8265.11178-s001.zip › Educational_Summary_Report_11178.pdf]

MedEdPORTAL® The AAMC Journal of Teaching and Learning Resources

Original Publication

Open Access

Essential Principles of Preoperative Assessment in Internal Medicine: A Case-Based Teaching Session

James C. Hudspeth, MD*, Michael Schwartz, MD, Patrick Fleming, MD, Thomas Ostrander, MD, Mara Eyllon, PhD

*Corresponding author: james.hudspeth@bmc.org

Abstract

Introduction: Preoperative assessment is a core competency for internal medicine residents, but one with limited educational resources available presently. Ideally, residencies would provide an introduction to this topic prior to their residents performing preoperative assessments in clinic or during internal medicine consultation rotations. **Methods:** We developed a 120-minute case-based teaching session on preoperative assessment for PGY 2 residents where they reviewed a series of cases, applied preoperative risk calculators, and made recommendations on medication management using the same online tools they employ while working clinically. Interspersed lecture sections reviewed guiding principles, detailed key trials, and explored nuances of the topic. We performed pre- and posttests of knowledge and also obtained learner feedback. **Results:** Thirty-three out of 40 participants completed the pre- and posttests. The session was rated highly ($M = 4.0$ out of 5) and was viewed as preferable to a lecture-based approach ($M = 4.4$ out of 5); mean participant knowledge improved from 11.7 to 17.5 ($p < .001$) out of 22 points possible. The most consistently offered feedback was to give more time for the session than the 120 minutes allotted. **Discussion:** A teaching session mixing lecture with review of composite cases and application of preoperative assessment tools with immediate feedback improved knowledge and was viewed as enjoyable and preferable to lecture alone by participants. We recommend providing more time for the teaching by increasing the session length from 120 minutes to 140 minutes.

Keywords
Preoperative Assessment, Internal Medicine, Perioperative Management, Case-Based Learning

Educational Objectives

By the end of this activity, learners will be able to:

1. Demonstrate the use of popular preoperative cardiac risk assessment tools.
2. Provide recommendations on the perioperative management of common medications.
3. Provide an evidence-based approach to reduce perioperative cardiac risk in typical preoperative situations.

Introduction

Consultation and perioperative medicine are important components of the practice of internal medicine, and the Accreditation Council for Graduate Medical Education mandates that internal medicine residency programs prepare their residents to be competent at “acting in a consultative role to other physicians.”[1] The perioperative period brings a range of risks for patients, with increasing risk for those who are older or have multiple medical comorbidities, and the medical complexity of surgical patients is generally rising over time.[2] While the surgical and anesthesia teams perform perioperative risk assessment and management for every patient undergoing surgery, general internal medicine consult services in both inpatient and outpatient settings play an important role in many United States hospitals for patients with higher medical complexity. Internists and family medicine physicians who provide primary care should be familiar with preoperative assessment for their primary care patients; internists offering inpatient care may be asked to provide both preoperative assessment and postoperative support for perioperative patients depending on the structure of clinical services at their institution. Thus, it is important that internal medicine residents have adequate training in these areas.

Citation:
Hudspeth JC, Schwartz M, Fleming P, Ostrander T, Eyllon M. Essential principles of preoperative assessment in internal medicine: a case-based teaching session. *MedEdPORTAL.* 2021;17:11178.
https://doi.org/10.15766/mep_2374-8265.11178

Copyright © 2021 Hudspeth et al. This is an open-access publication distributed under the terms of the Creative Commons Attribution license.

While most United States residents participate in medicine consult rotations during their training, their clinical experience and subsequent learning depend on case mix and volume, which are challenging to predict and standardize. Given this, it is vital that training programs offer a curriculum that covers the core elements of perioperative care. To our knowledge, only two open-access teaching materials have been published that address this topic. The first was written in 2006 and is significantly out of date.[3] The latter, a more recent publication from the Mayo Clinic group, details learning objectives and entrustable professional activities from a perioperative education curriculum but does not provide any of the teaching materials from that curriculum.[4] Several Curbsiders podcasts review key topics in perioperative medicine, but they are not designed to be a comprehensive introduction to the topic.[5] There is a Society for Hospital Medicine curriculum that provides a thorough review of perioperative medicine over 14 modules lasting 2 hours each; however, the curriculum is not open access and is too long to be readily used for resident education before an initial consult rotation.[6]

Here, we describe a case-based teaching session for PGY 2 internal medicine residents that discusses the components of an effective consultation as well as key concepts in the preoperative assessment of surgical patients, with a particular focus on cardiac risk assessment and medication management. This session uses an experiential, case-based approach targeted at internal medicine residents prior to their first rotation performing preoperative assessment.

Methods

At Boston Medical Center, the internal medicine consultation service performs a significant number of preoperative assessments on inpatients and is staffed by PGY 3 internal medicine residents. We developed a teaching session on preoperative medicine assessment for all PGY 2 internal medicine residents ($N = 47$). This session occurred as part of their monthly educational half day during a clinic week and was delivered to this cohort in May and June of 2019. Participation in the teaching was mandatory for residents who were not on vacation, but filling out the pre- and posttests was an optional research activity with Boston University Institutional Review Board approval.

We designed the session to be divided into two parts and delivered it over 120 minutes, including a 10-minute break. Required materials included a display for PowerPoint slides (Appendix A) as well as printed cases (Appendices B & C) that were handed out to the residents at the appropriate points. The session presumed that the facilitators had experience in perioperative medicine and that the learners had some internal medicine knowledge (the session likely could be provided to PGY 1 residents or fourth-year medical students with greater explanation).

Our teaching session aimed to provide residents with hands-on experience using the common tools in preoperative assessment, including preoperative cardiac risk calculators, a surgical risk calculator, and UpToDate as a resource for the perioperative management of medications (other point-of-care knowledge resources could be substituted for UpToDate). We also aimed to give residents an overview of the evidence base behind common perioperative situations. Appendix A, a PowerPoint slideshow (with extensive notes to allow easy use by facilitators), formed the backbone of the teaching session. It opened with essential concepts for providing high-quality consultations, then reviewed in an interactive fashion the American College of Cardiology/American Heart Association 2014 algorithm for preoperative cardiac risk assessment, the three most commonly used risk calculators, and some of the nuances pertaining to their use.

After the initial discussion, we divided the trainees into three subgroups, then provided each subgroup with a printed copy of one of three different cases from Appendix B. The three subgroups took between 10 and 15 minutes to review their respective cases, calculate cardiac risk using the calculators previously discussed, and provide recommendations for perioperative management with a focus on medications and whether a patient could proceed to operation. After this, the subgroups came back together, and each presented its recommendations to the other groups over 3-4 minutes, as though they were presenting consult recommendations to a consulting service. The PowerPoint included the recommended answers for each case and provided further evidence for these decisions.

We gave the trainees a short break, after which the PowerPoint continued with a discussion of preoperative anticoagulation. The three groups separated again to each review a different case from Appendix C, with a slightly shorter period this time (10 minutes), and finally reconvened to present their cases to the other teams, again with the PowerPoint providing the correct answers and evidence behind different decisions.

The workflow of the session is summarized as a time line in Figure 1.

Copyright © 2021 Hudspeth et al. This is an open-access publication distributed under the terms of the Creative Commons Attribution license.

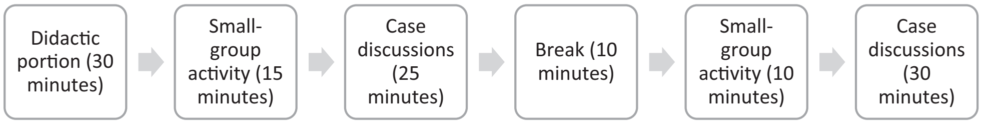

Figure 1. Session time line.

Trainees who opted into the evaluation portion filled out a 5-minute pretest (Appendix D; this questionnaire has been edited for clarity based on the peer review) discussed further below. After completing the session, the trainees filled out a posttest on the back of the same paper to ensure linked scores. This posttest included the same factual questions and rated the trainees' experiences in the session. The anonymous pre- and posttest scores as well as subjective ratings were abstracted into an Excel spreadsheet for statistical analysis.

The pretest survey consisted of seven questions. The first question, about the trainees' ability to assess a patient prior to an operation, was rated on a 5-point Likert-scale (1 = *none,* 5 = *excellent*). The second question asked whether they had previous experience with preoperative planning in prior residency rotations. The next five questions were knowledge questions about preoperative management, developed by the presenters to be straightforward with a clearly accurate response based on present guidelines; we reviewed these with two separate educators at our institution for input on clarity and clinical accuracy and incorporated their feedback.

The posttest included the same five knowledge questions, as well as two new questions about the trainees' impression of the session rated on 5-point Likert scales (1 = *unhelpful,* 5 = *excellent,* and 1 = *much worse,* 5 = *much better,* respectively) and one open-ended question regarding areas for improvement.

To assess improvements in knowledge, we used the data from knowledge questions at pretest and posttest, with a maximum possible score of 22 points across five questions. The data were nonnormally distributed, so we performed a nonparametric sign test to compare participants' knowledge scores before and after the session. In addition, we looked at participants' mean self-assessed ability to provide preoperative planning at pretest and their overall rating of the exercise, as well as their rating of the case-based method compared to a traditional lecture.

Results

A total of 33 participants completed both the pre- and posttests out of 40 PGY 2 residents who attended the sessions. Before participating in the session, participants on average reported that their ability to provide medical advice to a surgical team was minimal to moderate ($M = 2.4$, $SD = .50$), and 54% reported completing a prior rotation featuring preoperative assessment. At posttest, participants rated the session highly ($M = 4.0$, $SD = .68$, which corresponds to "great"). Additionally, participants overall preferred the case-based session compared to a lecture-style section (Table).

In terms of learning, participants' knowledge scores improved significantly ($p < .001$). At pretest, the mean score was 11.7 ($SD = 3.52$), and at posttest, the mean knowledge score was 17.5 ($SD = 2.44$). According to the sign test, 30 participants' scores improved, two remained the same, and only one declined after the session.

Eleven participants provided feedback on areas for improvement. All of these comments related to time, and 10 of the 11 suggested more time for the session as a whole given the scope of the topic.

Discussion

We developed a teaching session incorporating cases, application of the actual tools used in preoperative assessment, and lecture to provide our PGY 2 residents with skills and knowledge prior to their medicine consultation rotations in their PGY 3 year. This session received positive reviews from participants, who felt it was preferable to the typical lecture-based format, and demonstrated improved knowledge of fundamental topics in preoperative assessment and management.

The predominant feedback we received focused on the time allotted for the session being too little. The overall topic of perioperative medicine is quite large, and we worked to cover topics that we thought essential, while briefly discussing further nuances. We would recommend using a longer period of time,

Table. Self-Ratings of Pretest Ability and Posttest Perceptions of Exercise and Case-Based Delivery ($N = 33$)

Item	*M*	*SD*
Self-described ability (pretest)[a]	2.4	.50
Rating of exercise (posttest)[b]	4.0	.68
Rating of case-based approach vs. lecture (posttest)[c]	4.4	.70

[a]Rated on a 5-point scale (1 = *none,* 5 = *excellent*).
[b]Rated on a 5-point scale (1 = *unhelpful,* 5 = *excellent*).
[c]Rated on a 5-point scale (1 = *much worse,* 5 = *much better*).

Copyright © 2021 Hudspeth et al. This is an open-access publication distributed under the terms of the Creative Commons Attribution license.

MedEdPORTAL® The AAMC Journal of Teaching and Learning Resources

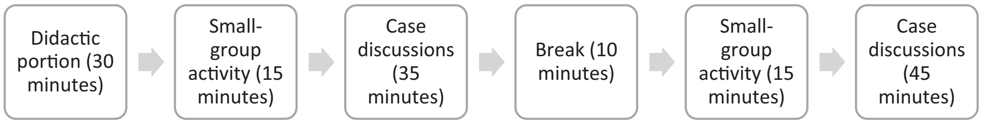

Figure 2. Longer session time line.

expanding from 120 minutes to 140, with a plan to spend 70 minutes on the first portion, take a 10-minute break, and then spend 60 minutes on the second section (or do the second half on a subsequent day); a time line summarizing this longer session approach is shown in Figure 2.

Alternatively, the session could be broken into three sections, each taking 50-60 minutes, taught over three separate sessions or over about 180 minutes (in which case, we would suggest doing the first portion through Appendix A, slide 23; the second session starting with the first set of cases through slide 45; and the final session starting with the second set of cases through the end of the slideshow).

We otherwise found that the educational flow of the session went well and was generally well regarded by the residents who experienced it. We developed the session in concert with our internal medicine consult service group and found it important to ensure that the recommendations for the teaching paralleled the practice of the group our residents worked with clinically. Four residents missed this session due to vacation; we provided them with the slideshow to work through independently, but we did not assess its impact due to their small number.

Our learning module is broadly generalizable in the United States for those seeking teaching materials about preoperative assessment for internal medicine residents, and the approaches listed are concordant with present guidelines. Some institutions may have different resources for medication assessment (e.g., DynaMed instead of UpToDate) or specific preoperative risk calculators that they prefer. We recommend that the teaching resources be reviewed and updated if an institution has specific protocols for the topics addressed.

Our primary limitation is that we did not assess for changes in behavior, and so, it is uncertain whether our participants will appropriately apply these skills in practice. That said, the goal of our session is not to make learners independent practitioners in preoperative assessment and management but to prepare residents for a supervised experience in clinic or on a consult rotation, such that they begin with a better appreciation of the approach to take and of the key studies underlying that approach. A secondary limitation is that this resource will become outdated over time as further studies are published; as a result, faculty employing it should be familiar with perioperative medicine from the internal medicine vantage and should review the slideshow prior to each use to ensure that the materials remain accurate. Additionally, as we did not run this session over multiple years, the residents exposed were all from one class, and different classes may have differences in knowledge acquisition from the session.

Our success using a case-based teaching session for a specific facet of internal medicine residency training raises the possibility of similar approaches to help prime residents before novel clinical experiences that require new knowledge or skills, such as a subspecialty rotation or night float.

Appendices

A. Preop Assessment and Management Slideshow.pptx

B. Cases 1-3.docx

C. Cases 4-6.docx

D. Pre- and Postassessment.docx

All appendices are peer reviewed as integral parts of the Original Publication.

James C. Hudspeth, MD: Assistant Professor, Department of Medicine, Boston University School of Medicine; ORCID: https://orcid.org/0000-0001-6431-4162

Michael Schwartz, MD: Assistant Professor, Department of Medicine, Boston University School of Medicine

Patrick Fleming, MD: Assistant Professor, Department of Medicine, Boston University School of Medicine

Thomas Ostrander, MD: Assistant Professor, Department of Medicine, Boston University School of Medicine

Mara Eyllon, PhD: Postdoctoral Researcher, Department of Medicine, Boston Medical Center

Disclosures
None to report.

Copyright © 2021 Hudspeth et al. This is an open-access publication distributed under the terms of the Creative Commons Attribution license.

Funding/Support
None to report.

Ethical Approval
The Boston University Institutional Review Board approved this study.

References

1. *ACGME Program Requirements for Graduate Medical Education in Internal Medicine*. Accreditation Council for Graduate Medical Education; 2020.
2. Moridzadeh RS, Sanaiha Y, Madrigal J, Antonios J, Benharash P, Baril DT. Nationwide comparison of the medical complexity of patients by surgical specialty. *J Vasc Surg*. 2021;73(2):683-688.E2. https://doi.org/10.1016/j.jvs.2020.05.072
3. OLeary K. Case-based curriculum for general medicine consultation. *MedEdPORTAL*. 2006;2:213. https://doi.org/10.15766/mep_2374-8265.213
4. Raslau D, Kasten MJ, Kebede E, Mohabbat A, Ratrout B, Mikhail M. Developing a comprehensive perioperative education curriculum for internal medicine residency training. *J Educ Perioper Med*. 2017;19(3):E608. https://doi.org/10.46374/volxix-issue3-raslau
5. Perioperative collection. Curbsiders. Accessed April 17, 2021. https://thecurbsiders.com/tag/perioperative
6. SHM Consults Core Curriculum. Society for Hospital Medicine. June 19, 2017. Accessed April 17, 2021. https://www.shmlearningportal.org/content/shm-consults-core-curriculum

Received: September 23, 2020
Accepted: May 26, 2021
Published: August 5, 2021

Copyright © 2021 Hudspeth et al. This is an open-access publication distributed under the terms of the Creative Commons Attribution license.
